# A high-quality chromosomal genome assembly of *Diospyros oleifera* Cheng

**DOI:** 10.1093/gigascience/giz164

**Published:** 2020-01-16

**Authors:** Yujing Suo, Peng Sun, Huihui Cheng, Weijuan Han, Songfeng Diao, Huawei Li, Yini Mai, Xing Zhao, Fangdong Li, Jianmin Fu

**Affiliations:** 1 Key Laboratory of Non-timber Forest Germplasm Enhancement & Utilization of State Administration of Forestry and Grassland, No. 3 Weiwu Road, Jinshui District, Zhengzhou 450003, China; 2 Non-timber Forest Research and Development Center, Chinese Academy of Forestry, No. 3 Weiwu Road, Jinshui District, Zhengzhou 450003, China; 3 National Innovation Alliance of Persimmon Industry, No. 3 Weiwu Road, Jinshui District, Zhengzhou 450003, China; 4 Novogene Bioinformatics Institute, Zone A10 Jiuxianqiao North Road, Chaoyang District, Beijing 100083, China

**Keywords:** *Diospyros oleifera*, chromosome-level genome assembly, Hi-C assembly, tannin synthase genes, gene expansion, whole-genome duplication

## Abstract

**Background:**

*Diospyros oleifera* Cheng, of the family Ebenaceae, is an economically important tree. Phylogenetic analyses indicate that *D. oleifera* is closely related to *Diospyros kaki* Thunb. and could be used as a model plant for studies of *D. kaki*. Therefore, development of genomic resources of *D. oleifera* will facilitate auxiliary assembly of the hexaploid persimmon genome and elucidate the molecular mechanisms of important traits.

**Findings:**

The *D. oleifera* genome was assembled with 443.6 Gb of raw reads using the Pacific Bioscience Sequel and Illumina HiSeq X Ten platforms. The final draft genome was ∼812.3 Mb and had a high level of continuity with N50 of 3.36 Mb. Fifteen scaffolds corresponding to the 15 chromosomes were assembled to a final size of 721.5 Mb using 332 scaffolds, accounting for 88.81% of the genome. Repeat sequences accounted for 54.8% of the genome. By *de novo* sequencing and analysis of homology with other plant species, 30,530 protein-coding genes with an average transcript size of 7,105.40 bp were annotated; of these, 28,580 protein-coding genes (93.61%) had conserved functional motifs or terms. In addition, 171 candidate genes involved in tannin synthesis and deastringency in persimmon were identified; of these chalcone synthase (*CHS*) genes were expanded in the *D. oleifera* genome compared with *Diospyros lotus, Camellia sinensis*, and *Vitis vinifera*. Moreover, 186 positively selected genes were identified, including chalcone isomerase (*CHI*) gene, a key enzyme in the flavonoid-anthocyanin pathway. Phylogenetic tree analysis indicated that the split of *D. oleifera* and *D. lotus* likely occurred 9.0 million years ago. In addition to the ancient γ event, a second whole-genome duplication event occurred in *D. oleifera* and *D. lotus*.

**Conclusions:**

We generated a high-quality chromosome-level draft genome for *D. oleifera*, which will facilitate assembly of the hexaploid persimmon genome and further studies of major economic traits in the genus *Diospyros*.

## Data Description

### Background

**Figure 1:**
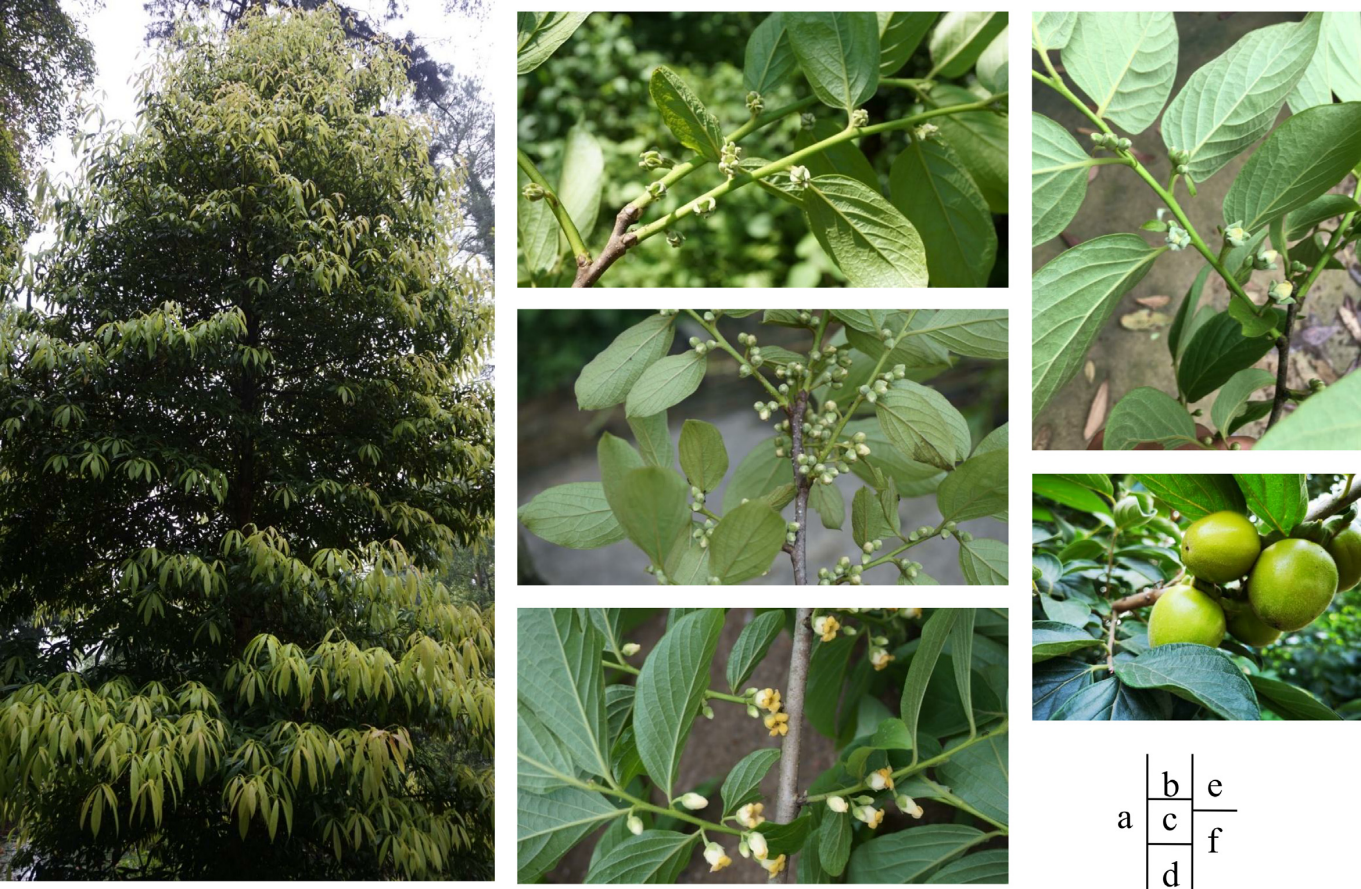
Images of*D. oleifera*. Mature tree (a), gynoecious type (b), androecious type (c), andromonoecious type (d), monoecious type (e), fruit (f).

*Diospyros* is the largest genus in the family Ebenaceae, comprising >500 species, of which the ebony and fruit have considerable economic value. The ebony of >20 species of *Diospyros* (including *Diospyros reticulata* from Africa and *Diospyros ebenum* and *Diospyros ferrea* from Asia) is used commercially for arts, crafts, and decorative building materials. In addition, *Diospyros kaki, Diospyros oleifera*, and *Diospyros lotus* are important species for fruit production; indeed, *D. kaki* is one of the most widely distributed fruit trees worldwide. However, most *D. kaki* cultivars are hexaploid (2n = 6× = 90) or nonaploid (2n = 9× = 135) and their progenitor, origin, and polyploidization mechanisms are unclear, which has hampered molecular breeding. *D. oleifera* is diploid (2n = 2× = 30) and its fruit contains large quantities of tannins, important raw materials for the production of persimmon paint (Fig. 1). *D. oleifera* is also frequently selected as stock for grafting of persimmon (*D. kaki*). Phylogenetic analyses based on the chloroplast genome and protein-coding, intergenic, and intron sequences have indicated that *D. oleifera* is closely related to *D. kaki* and could be used as a model plant for studies of *D. kaki* [1]. Therefore, analysis of the genome of *D. oleifera* will contribute to auxiliary assembly of the hexaploid persimmon genome.

The cultivars of hexaploid *D. kaki* are classified into 4 groups on the basis of the mode of astringency loss by the fruits: pollination-constant non-astringent (PCNA), pollination-variant non-astringent, pollination-constant astringent, and pollination-variant astringent [2]. PCNA is the most desirable type because the fruits are edible without any postharvest treatment. Owing to the complexity of the hexaploid *D. kaki* genome and the lack of genomic sequence information, the natural deastringency mechanism of China-PCNA *D. kaki* is still unclear. Therefore, this study, which identifies tannin synthesis–related genes based on genomic sequence information in *D. oleifera*, will be helpful for subsequent studies on natural deastringency mechanisms of China-PCNA *D. kaki*.

The sexuality of *Diospyros spp*. is diverse. For example, diploid *D. lotus* is dioecious, including gynoecious (bearing only female flowers) and androecious (bearing only male flowers) types, whereas both diploid *D. oleifera* and hexaploid *D. kaki* contain gynoecious, androecious, monoecious (bearing both female and male flowers), polygamomonoecious (bearing female, male, and hermaphroditic flowers), and andromonoecious (bearing male and hermaphroditic flowers) types. The sexuality of *D. oleifera* and *D. kaki* is also labile. For example, gynoecious *D. oleifera* and *D. kaki* trees may occasionally bear male flowers [3]. The mechanism underlying such sexual diversity and lability in diploid *D. oleifera*, in contrast to the dioecy of diploid *D. lotus* deserves further investigation; greater understanding of this mechanism will also help to uncover the complicated mechanism of sexual diversity and lability in *D. kaki*. The genomic sequence information of *D. oleifera* obtained in the present study will be valuable for studies on sexuality in *D. oleifera* and other *Diospyros spp*.

Here, we performed a high-quality chromosome-level reference genome assembly for *D. oleifera* (NCBI:txid227308) using the long reads generated by the Pacific Biosciences (PacBio) DNA sequencing platform and Hi-C data. The high quality (in terms of completeness and continuity) of the genome will facilitate both assembly of the hexaploid persimmon genome and further studies of major economic traits in the genus *Diospyros*.

### Genomic DNA extraction, library construction, sequencing, and genome size estimation

Genomic DNA was extracted from fresh leaves of *D. oleifera* using a DNAsecure Plant Kit (Tiangen Biotech, Beijing, China). A short-read genomic library was prepared using the TruSeq DNA PCR-Free LT Library Kit (Illumina, San Diego, CA, USA). Five paired-end genomic sequence libraries with a gradient insert size of 250–450 bp were constructed and sequenced on the Illumina HiSeq X Ten platform. A total of 104.02 Gb of raw sequence data (119.78-fold coverage of the *D. oleifera* genome) were used for genome assembly (Supplementary Table S1).

At least 10 μg of sheared DNA was required to generate the 40-kb insert library. Preparation of the single-molecule real-time (SMRT) cell template involved DNA concentration, damage repair, end repair, ligation of hairpin adapters, and template purification. Subsequently, the genome was sequenced on the PacBio Sequel platform (Pacific Biosciences, Menlo Park, CA, USA). A total of 99.76 Gb of raw sequence data (114.88-fold coverage of the *D. oleifera* genome) were used for genome assembly (Supplementary Table S1).

To produce a 10X genome library, ∼1 ng of input DNA (50 kb length) was used for the GEM reaction during PCR, and 16-bp barcodes were introduced into droplets. Next, the droplets were fractured following purification of the intermediate DNA library. The library comprised 109.88 Gb (126.53-fold coverage of the *D. oleifera* genome) and was sequenced using 150-bp paired-end reads on the Illumina HiSeq X platform (Supplementary Table S1).

One Dovetail Hi-C library was prepared as described previously [4]. Briefly, for each library, chromatin was fixed in place in the nucleus using formaldehyde and then extracted. Fixed chromatin was digested with DpnII, the 5′ overhangs were filled using biotinylated nucleotides, and free blunt ends were ligated. After ligation, crosslinks were reversed, and DNA was separated from protein. Purified DNA was treated to remove biotin outside of the ligated fragments, sheared to a mean fragment size of ∼350 bp, and used to create sequence libraries with NEBNext Ultra enzymes (New England Biolabs, Ipswich, MA, USA) and Illumina-compatible adapters. Biotin-containing fragments were isolated using streptavidin beads before PCR enrichment of the libraries; the libraries were next sequenced on the Illumina HiSeq PE150 platform. A total of 98.24 Gb of reads was produced for the libraries. Together, these Dovetail Hi-C library reads provided 113.12-fold physical coverage of the genome (Supplementary Table S1).

High-quality paired-end reads from *D. oleifera* were used to generate 17-mer frequency information by *k*-mer analysis [5]. The 17-mer distribution was dependent on the characteristics of the genome and followed a Poisson distribution (Supplementary Fig. S1). We estimated the genome to be 868.41 Mb in size with a heterozygosity of 1.08% (Supplementary Table S2).

### *De novo* assembly of *D. oleifera*

**Figure 2:**
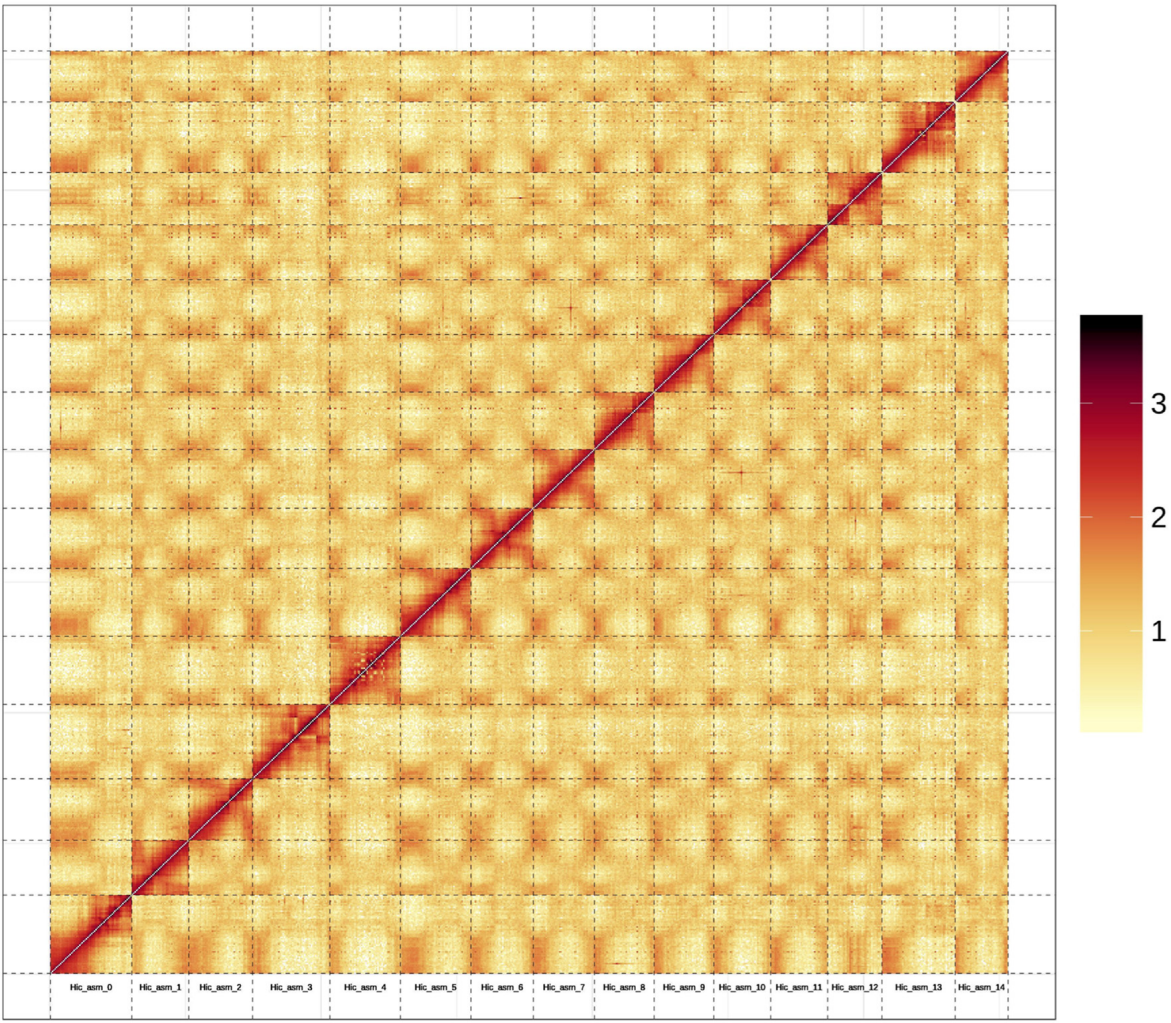
Hi-C interaction heat map for *D. oleifera* genome showing interactions among 15 chromosomes.

**Figure 3:**
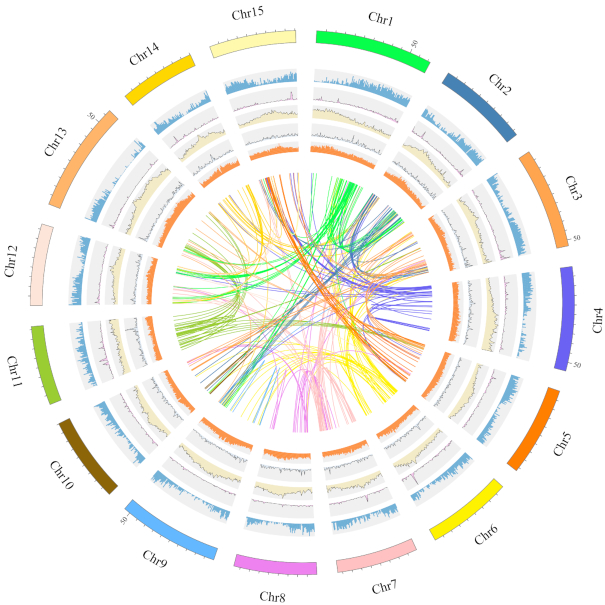
*D. oleifera* genome features. Tracks from outside to inside are as follows: the distribution of gene density, LINE retrotransposons density, LTR retrotransposons density, DNA transposons density, GC density, and syntenic blocks.

*De novo* assembly of the long reads generated by SMRT sequencing was performed using FALCON [6, 7] v. 0.3 (Falcon, RRID:SCR_016089). Briefly, we first selected the longest coverage of subreads as seeds for error correction. Next, the data were filtered and assembled (length_cutoff_pr = 4000, max_diff = 100, and max_cov = 100). A total of 2,986 contigs was assembled with a total length of 806.74 Mb (accounting for ∼92.9% of the estimated genome), an N50 of 2.92 Mb, and a longest contig of 14.72 Mb (Table 1). The primary contigs (p-contigs) were polished using Quiver [8] by aligning SMRT reads, which produced a genome of 812.37 Mb and an N50 of 2.94 Mb. Finally, Pilon [9] v. 1.22 (Pilon, RRID:SCR_014731) was used to perform the second round of error correction with the short paired-end reads generated by the Illumina HiSeq platform, resulting in a genome of 811.09 Mb and a longest contig of 14.81 Mb (Table 1). For the scaffolding step, Long Ranger (v. 2.1.2) [10] was applied to build scaffolds using the 10X data. FragScaff [11] (v. 1–1) was used to build superscaffolds from the barcoded sequencing reads. The final assembly contained 2,812 scaffolds and had a total length of 812.32 Mb, representing ∼93.54% of the genome estimated by *k*-mer analysis. The sizes of the longest contig and scaffold were 14.82 and 17.43 Mb, respectively, and the N50s were 2.94 and 3.36 Mb, respectively (Table 1). Subsequently, the Hi-C sequencing data were aligned to the assembled scaffolds by BWA-mem [12] (v. 0.7.8), and the scaffolds were clustered onto chromosomes with LACHESIS (LACHESIS, RRID:SCR_017644) [13]. Among the 2,812 scaffolds, 332 were grouped into the 15 chromosomes, with maximum and minimum lengths of 61.45 and 40.21 Mb, respectively (Fig. 2). The final genome was 721.45 Mb and the N50 was 33.5 Mb, accounting for 88.81% of the total genome (Supplementary Table S3, Fig. 3). The continuity and integrity of the assembly for *D. oleifera* is significantly better than that of the published *D. lotus* genome, which final genome was 945.63 Mb with contigs N50 of 0.65 Mb, and 746.09 Mb (78.9%) was assembled into the 15 pseudomolecules [14].

**Table 1:** Summary of the *D. oleifera* genome assembly using PacBio long-read, Illumina reads, and 10X genomics data

Program	Sample ID	Length		No.	
		Contig (bp)	Scaffold (bp)	Contig	Scaffold
Falcon	Total	806,744,914		2,986	
	N50	2,916,360		72	
Quiver	Total	812,369,941		2,986	
	N50	2,938,972		72	
Pilon	Total	811,094,501		2,986	
	N50	2,937,127		72	
10X	Total	811,094,501	812,323,628	2,986	2,812
	Maximum	14,814,786	17,432,797		
	No. ≥ 2,000			2,803	2,629
	N50	2,937,127	3,359,874	71	62
	N60	2,314,962	2,662,781	103	89
	N70	1,622,862	1,911,995	144	125
	N80	790,034	1,007,083	214	182
	N90	196,816	257,477	421	333

### Assessment of the assembled genome

To estimate the quality of the assembled genome, the short reads were mapped back to the consensus genome using BWA (BWA, RRID:SCR_010910); the overall mapping rate was 98.19%, suggesting that the assembly contained comprehensive genomic information (Supplementary Table S4). The completeness of gene regions was assessed using Core Eukaryotic Gene Mapping Approach (CEGMA, RRID:SCR_015055) [15] and BUSCO (BUSCO, RRID:SCR_015008) [16] to evaluate the completeness of the assembled genome. The CEGMA assessment showed that 232 (93.55%) of 248 core eukaryotic genes were covered (Supplementary Table S5). In contrast, 89.4% of complete BUSCOs were detected and 6.6% were missing (Supplementary Table S6), indicating that the assembled genome had a high level of completeness.

### Repetitive elements identification of *D. oleifera*

The *D. oleifera* genome was subjected to annotation of repetitive sequences—transposable elements (TEs) and tandem repeats. RepeatMasker [17, 18] v. 4.0.5 (RepeatMasker, RRID:SCR_012954) was used to detect TEs in a repeat library derived from a known repeat library (Repbase, v. 15.02) and a *de novo* repeat library generated using RepeatModeler [4] v. 1.0.5 (RepeatModeler, RRID:SCR_015027), RepeatScout [19] v. 1.0.5 (RepeatScout, RRID:SCR_014653), Piler v. 1.0 (PILER, RRID:SCR_017333), and LTR_FINDER [20] v. 1.0.7 (LTR_Finder, RRID:SCR_015247). RepeatProteinMask [18] (v. 4.0.5) was used to detect TEs in the *D. oleifera* genome by comparison with a TE database. Tandem repeats were identified using Tandem Repeats Finder [21] (v. 4.0.7).

Repetitive sequences comprised 54.8% of the *D. oleifera* genome, among which TEs accounted for 53.03%. The most frequently detected TEs were long terminal repeat (LTR) retrotransposons (46.73%), followed by DNA TEs (4.17%). Of the LTRs, 26.63% and 14.40% were Ty3/Gypsy and Ty1/Copia, respectively (Table 2).

**Table 2:** Classification of repetitive elements in *D. oleifera* genome

**Program**	**Repeat size (bp)**	**% of genome**
TRF	79,886,467	9.83
Repeatmasker	408,623,327	50.3
Proteinmask	22,154,795	2.73
Total	445,187,963	54.8
**Transposon elements**	**Transposon element length (bp)**	**% in genome**
DNA	33,844,732	4.17
LINE	13,187,364	1.62
SINE	74,819	0.01
LTR		
Total	379,582,766	46.73
Gypsy	216,328,284	26.63
Copia	116,970,626	14.40
Other	46,283,856	5.70
Unknown type	10,513,280	1.29
Total	430,778,122	53.03

### Genomic RNA extraction, library construction, sequencing

For RNA sequencing (RNA-seq), we collected different tissues of *D. oleifera* from the same plant used for genome sequencing, including material from leaf, root, seed, stem, and fruit. Total RNAs were extracted using TRIzol® Reagent (Thermo Fisher Scientific, Waltham, Massachusetts, USA) according to the manufacturer's instructions. RNA-seq was performed using an Illumina platform.

### Annotation of protein-coding genes

*De novo*, homolog-based, and RNA-seq–based predictions were used to annotate the protein-coding genes in the *D. oleifera* genome. The following *ab initio* gene prediction software packages were used to predict genes: Augustus [22, 23] v. 3.0.2 (Augustus, RRID:SCR_008417), Genescan [24] v. 1.0 (GENSCAN, RRID:SCR_012902), Geneid [25] (v. 1.4), GlimmerHMM [26] v. 3.0.2 (GlimmerHMM, RRID:SCR_002654), and SNAP [27] (SNAP, RRID:SCR_007936; 2013-11-29). The protein sequences of 7 species (including *Arabidopsis thaliana* and *Daucus carota*) were downloaded from Ensembl or the NCBI databases. Homologous sequences were aligned against the *D. oleifera* genome using TBLASTN [28] (v. 2.2.26, E-value ≤ 1E-05; TBLASTN, RRID:SCR_011822). Genewise [29] (v. 2.2.0) was used to predict gene models based on the aligned sequences. The RNA-seq data were assembled into the unique sequences of transcripts by mapping the RNA-seq data to the *D. oleifera* genome using TopHat [30] v. 2.0.8 (TopHat, RRID:SCR_013035) and Cufflinks [31, 32] v. 2.1.1 (Cufflinks, RRID:SCR_014597) for transcript assembly. Alternatively, Trinity [33] v. 2.1.1 (Trinity, RRID:SCR_013048) was used to assemble the RNA-seq data, and the gene structures were improved using PASA [34, 35] software (r20140417; PASA, RRID:SCR_014656). A weighted and non-redundant gene set was generated by merging all of the gene models predicted by the above 3 approaches with EVM v. 1.1.1 (EVM, RRID:SCR_014659) [36]. PASA was applied to adjust the gene models generated by EVM. The final reference gene set contained 30,530 protein-coding genes with mean transcript size of 7,105.4 bp, an average coding sequence size of 1,080.74 bp, and a mean number of exons per gene of 4.62 (Supplementary Table S7). The number of annotated genes in this genome is less than that in the *D. lotus* genome (40,532 genes).

### Functional annotation

Functional annotation of protein-coding genes was performed according to the best BLAST hit by BLASTP (v. 2.2.28, E-value ≤ 1E−05; BLASTP, RRID:SCR_001010) searching of the SwissProt, TrEMBL [37], and NCBI non-redundant (NR) protein databases. Motifs and domains were annotated by searching the Pfam, PRINTS, PROSITE, ProDom, and SMART InterPro (v. 29.0) databases using InterProScan [38] v. 4.8 (InterProScan, RRID:SCR_005829). The Gene Ontology term for each gene was annotated by Blast2GO (Blast2GO, RRID:SCR_005828) [39]. Additionally, the gene sets were mapped to KEGG [40] (v. 53) pathways to identify the best match classification for each gene (BLASTp E-value ≤ 1E−05). Finally, 28,580 protein-coding genes (93.61% of total 30,530 genes) had conserved functional motifs or functional terms—92.03% (28,098), 84.16% (25,695), and 71.21% (21,739) of the genes in NR, InterPro, and KEGG, respectively (Supplementary Table S8).

### Annotation of non-coding RNAs

Transfer RNA (tRNA) genes were predicted using tRNAscan-SE software [41] v. 1.4 (tRNAscan-SE, RRID:SCR_010835) with the default parameters. Ribosomal RNAs (rRNAs) were annotated based on their level of homology with the rRNAs of several species of higher plants (not shown) using BLASTN with an E-value of 1e−5. The microRNA (miRNA) and small nuclear RNA (snRNA) fragments were identified by searching the Rfam database (v. 11.0) using INFERNAL [42, 43] v. 1.1 (Infernal, RRID:SCR_011809) software. Finally, 564 miRNAs, 507 tRNAs, 2,207 rRNAs, and 803 snRNAs were identified, which had average lengths of 114.69, 74.82, 161.40, and 111.54 bp, respectively (Supplementary Table S9).

### Identification of tannin synthase genes in *D. oleifera*

Given the importance of tannin production in *D. oleifera*, we identified genes within the tannin biosynthesis pathway, which include the chorismic acid pathway, phenylpropane metabolic pathway, flavonoid-anthocyanin pathway, and proanthocyanidin-specific pathway (Supplementary Fig. S1). All of the synthase genes involved in the 4 pathways, as well as several closely related transcription factors (TFs) including WD40 and WIP-ZF, were identified by aligning to reference genes downloaded from NCBI [44] or The Arabidopsis Information Resource [45] using Blastp (E-value < 1E−5, identity ≥ 50%, and coverage ≥ 50%). A Pfam HMMER search was used to filter genes that did not contain the corresponding domain. TFs including MYB, MYC, and WRKY were identified and classified into different families using the iTAK pipeline (v. 1.7) [46]. As a result, 171 genes and 380 TFs were identified, of which 13, 59, and 21 genes were involved in the phenylpropane metabolic pathway, flavonoid-anthocyanin pathway, and proanthocyanidin-specific pathway, respectively. We also detected 18 genes encoding transport proteins such as glutathione S-transferase (GST) and multi-drug and toxic compound extrusion transporter (MATE), which were closely related to transmembrane transport of tannin. We identified the key genes of acetaldehyde metabolism, such as *ADH* (10), *ALDH* (19), and *PDC* (5), which were related to deastringency in persimmon (Supplementary Table S10). The tannin synthase genes and TFs identified in this study will provide a basis for molecular breeding of persimmon tannins.

### Gene family cluster, phylogenetic tree construction, and divergence time estimation

Eleven other sequenced plant species were used to investigate the evolution of *D. oleifera*, including 8 asterids (*D. lotus, Primula veris, Rhododendron delavayi, Camellia sinensis, Actinidia chinensis, Daucus carota, Coffea canephora*, and *Solanum lycopersicum*) and 3 rosids plants (*A. thaliana, Vitis vinifera*, and *Cucumis melo*). Gene families were generated by Orthofinder [47, 48] (v. 2.3.1). First, nucleotide and protein data of 10 species were downloaded from Ensembl (Release 70) and NCBI. Before executing an “all against all” BLASTP (E-value ≤ 1E−07) program, the longest transcript was selected from alternatively spliced transcripts of 1 gene, and genes with ≤50 amino acids were removed. The alignments with high-scoring segment pairs were conjoined for each gene pair by SOLAR (V0.0.19) [49]. After clustering, 19,722 gene families were detected in *D. oleifera* and 11 other species, of which 5,599 gene families and 221 single-copy orthologs were shared by 12 species. Among the 5 Ericales species (*D. oleifera, D. lotus, A. chinensis, R. delavayi*, and *C. sinensis*), 177 gene families consisting of 312 genes were unique to *D. oleifera* (Supplementary Fig. S3). GO enrichment analysis of these genes indicated that 98 genes had conserved functional terms that were significantly enriched in GO terms of zinc ion binding, proteolysis, and nutrient reservoir activity. In addition, 4 and 1 of these genes were involved in the carbohydrate metabolic process and aldehyde metabolic process, respectively, which may play roles in the carbohydrate accumulation and deastringency of fruit in *D. oleifera*.

**Figure 4:**
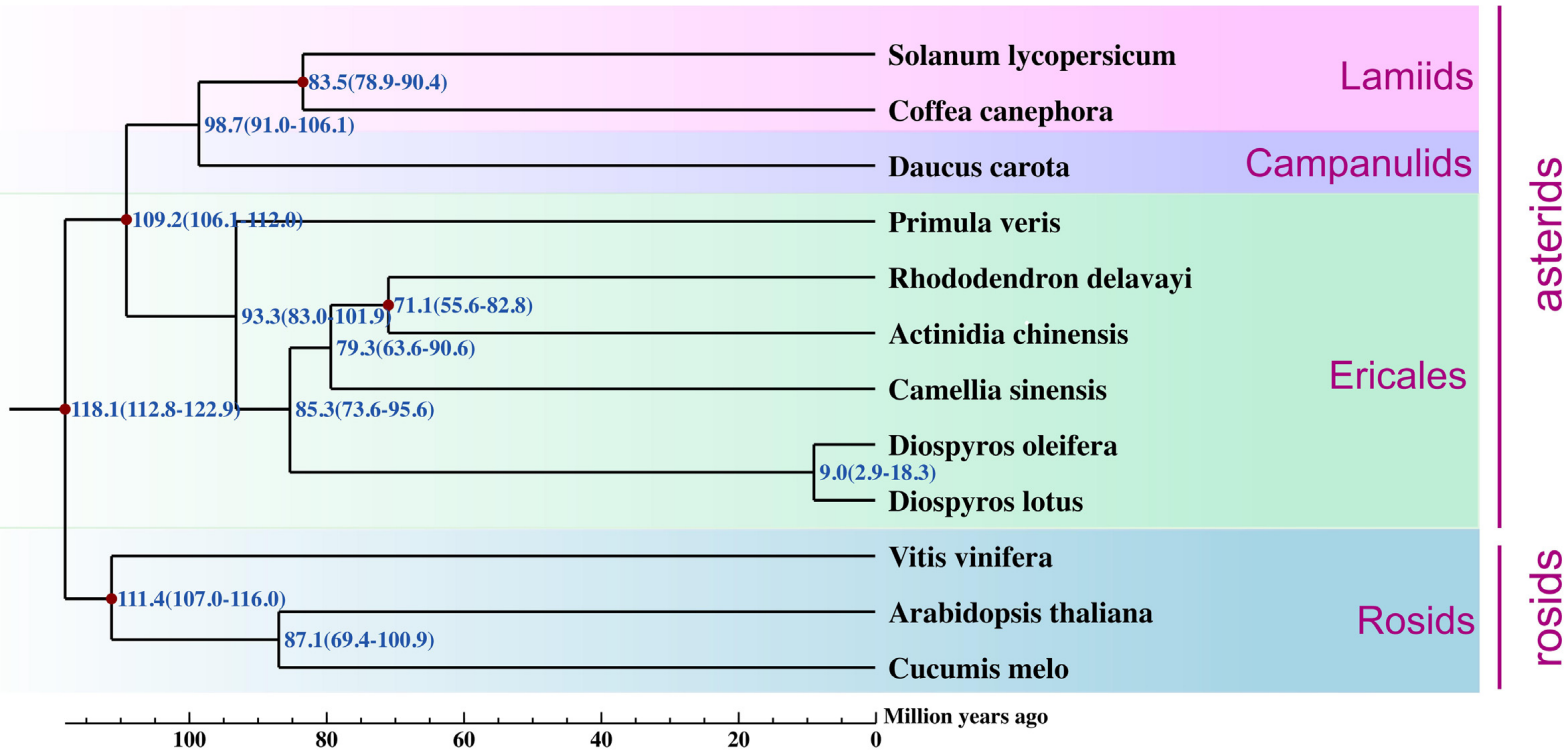
The phylogenetic relationships and divergence time estimation of *D. oleifera* with other plant species. Numbers outside the brackets indicate divergence time, and the condifence ranges are shown in parentheses.

A phylogenetic tree of the 12 plant species was constructed using Orthofinder (OrthoFinder, RRID:SCR_017118) based on a phylogenetic tree constructed by FastME [50] (v. 2.1.5). Gene trees were inferred for each orthogroup by aligning the sequences using mafft-linsi and inferring a maximum likelihood tree from this alignment using FastTree (FastTree, RRID:SCR_015501). DLCpar was used to reconcile these gene trees with the known species tree. Then, the mcmctree program of PAML [51, 52] v. 4.5 (PAML, RRID:SCR_014932) was applied to estimate divergence time among 12 species using the 221 shared single-copy orthologs with main parameters burn-in = 100 000, sample-number = 100 000, and sample-frequency = 2. 4 calibration points were selected from the TimeTree website [53] as normal priors to restrain the age of the nodes. The phylogenetic tree confirmed the grouping of Angiospermae. The split of *D. oleifera* and *D. lotus* was estimated at 9.0 million years ago (Fig. 4).

### Expansion and contraction of gene families

We determined the expansion and contraction of the gene families by comparing the cluster size differences between the ancestor and each species using the CAFÉ program [54]. For parameter Settings: Gene families with size significantly changed for species/branch: viterbi p < 0.05, and the others are the default parameters. A random birth and death model was used to study changes in gene families along each lineage of the phylogenetic tree. A probabilistic graphical model was introduced to calculate the probability of transitions in gene family size from parent to child nodes in the phylogeny. Using conditional likelihoods as the test statistics, we calculated the corresponding *P*-value for each lineage; a *P*-value of 0.05 was used to identify families that were significantly expanded and contracted. Compared with the common ancestor of *D. oleifera* and *D. lotus*, 175 gene families (1,896 genes) have expanded in *D. oleifera* (Supplementary Fig. S4); these were enriched in several KEGG pathways including ubiquitin-mediated proteolysis, ABC transporters, and carbon fixation in photosynthetic organisms. By contrast, 333 gene families (1,021 genes) were contracted in *D. oleifera*; these were enriched in several KEGG pathways including plant−pathogen interaction, phenylpropanoid biosynthesis, and cyanoamino acid metabolism.

In addition, the reference sequences of tannin synthase genes identified in *D. oleifera* were used for a BLAST query to identify homologous genes in *D. lotus, C. sinensis*, and *V. vinifera*. A sequence with similarity greater than the cut-off (50%) and coverage greater than the cut-off (50%) was selected as a preliminary candidate gene, followed by searching for the domain using hmmsearch. When the query was identical with that in the subject, the candidate gene was retained. A Fisher exact test (*P*-value ≤ 0.05) was performed on the number of individual genes related to tannin synthesis in the genomes of the 4 species to see whether the corresponding gene had expanded or contracted. Compared with *D. lotus, C. sinensis*,and*V. vinifera*, chalcone synthase (*CHS*) genes had expanded in the *D. oleifera* genome (11 genes in *D. oleifera*, 7 genes in *D. lotus*, 3 genes in *C. sinensis*, and 1 gene in *V. vinifera; P-*value = 0.0089) (Supplementary Table S11). CHS is the first key enzyme in the flavonoid-anthocyanin pathway; expansion of the *CHS* gene may be related to the abundant tannin production in *D. oleifera*. In addition, the expression of the*CHS* gene in different tissues of *D. oleifera* was analyzed using transcriptome data. The result showed that the expression of *CHS* genes was spatiotemporal specific, with 3 genes highly expressed in leaves, 6 in roots, and 1 in seeds (Supplementary Fig. S5). A contraction of laccase (*LAC*) genes that were responsible for the polymerization of persimmon tannin monomers [55, 56] was observed in *D. oleifera* compared with *V. vinifera* (21 *LAC* genes in *D. oleifera* and 53 in *V. vinifera*). This phenomenon may explain the difference of tannin types, which were defined according to the polymerization level of tannin monomers between *D. oleifera* and *V. vinifera*.

### Positively selected genes in *D. oleifera*

To understand the evolution of *D. oleifera*, positive selection analysis was performed to study the adaptive evolution of genes. The coding sequence (CDS) alignments of 789 single-copy gene families in *D. oleifera, D. lotus, A. chinensis, P. veris, R. delavayi*, and *S. lycopersicum* were generated using MUSCLE (MUSCLE, RRID:SCR_011812). Gblocks (Gblocks, RRID:SCR_015945) [57] was applied to filter poorly aligned positions and divergent regions of the CDS alignments. With *D. oleifera* as the foreground branch, positive selection sites were detected on the basis of branch-site models [58] of PAML [51] using the CDS alignments. *P*-values were computed using the χ^2^ statistic and adjusted by false discovery rate method. Finally, 186 genes were positively selected in *D. oleifera* (Supplementary Table S12). Among them, chalcone isomerase (*CHI*) gene, a key enzyme in the flavonoid-anthocyanin pathway, was found to be positively selected (ID: evm.model.original_scaffold_909.101). The positive selection of the *CHI* gene may be one of the reasons why *D. oleifera* is different from other species in producing abundant tannin.

### Whole-genome duplication and macrosynteny analysis

**Figure 5:**
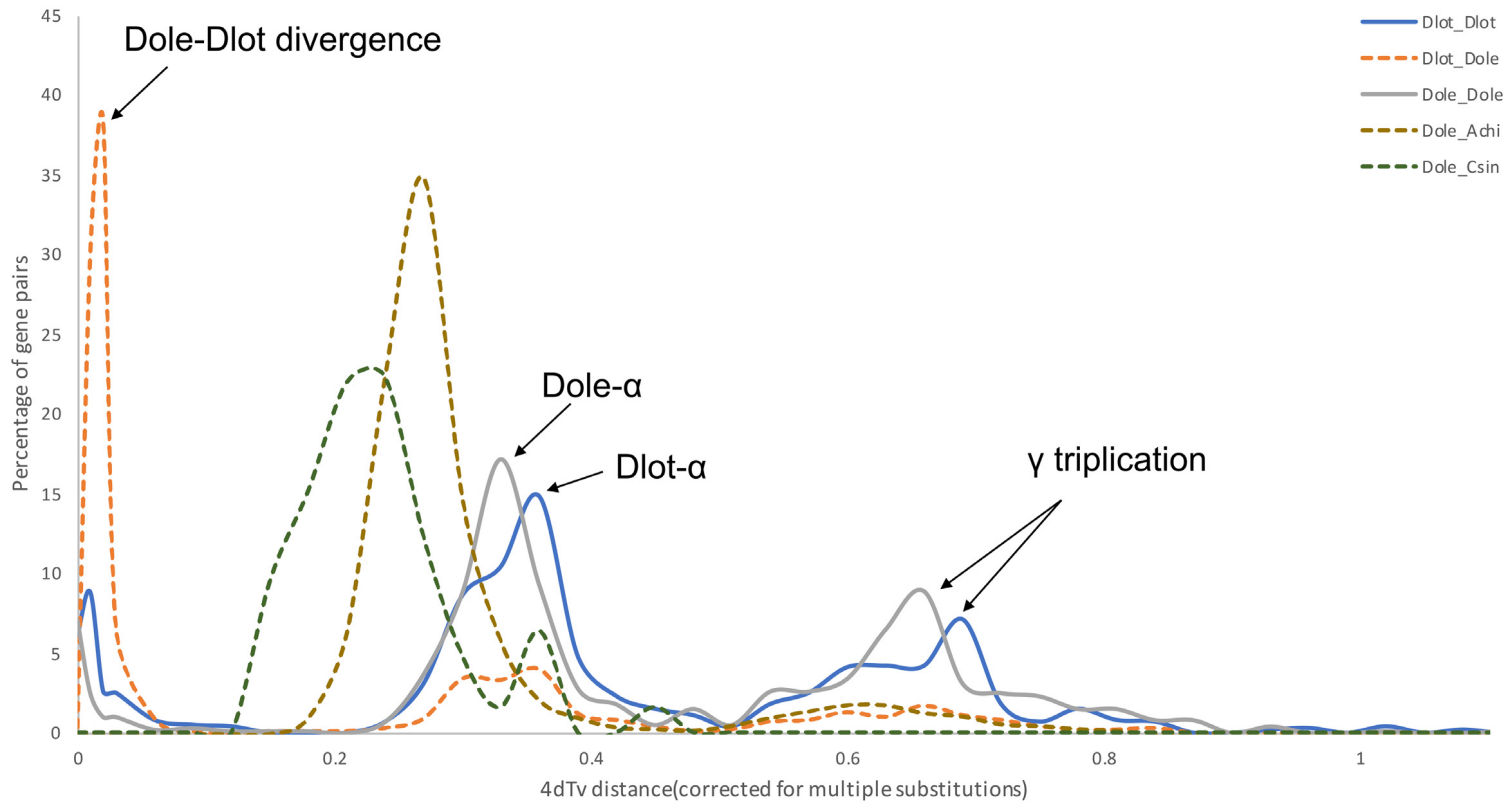
Whole-genome duplication analysis of *D. oleifera* genome.

We used BLASTP (E-value < 1E−5) to perform homolog and paralog searches with *D. oleifera* and other genomes (*A. chinensis, C. canephora, C. sinensis*), and MCScanX (s = 5, e = 1e−5) [59] was used to detect syntenic blocks. Then, transversion substitutions at 4-fold degenerate sites (4dtv) rates for all syntenic genes were calculated to identify putative whole-genome duplication (WGD) or species split events in *D. oleifera*. In addition to the ancient WGD event that occurred in all dicot species, γ event (all core eudicots share an ancient WGD, 4dtv = 0.66), a second WGD event occurred in *D. oleifera* and *D. lotus* (4dtv = 0.33/0.36) that might have contributed to the divergence of Ebenaceae with *A. chinensis* and *C. sinensis* (Fig. 5). We obtained 431 syntenic blocks between *D. oleifera* and *D. lotus*. On the whole, except for the translocation of some loci, the sequence of genes between *D. oleifera* and *D. lotus* was relatively conservative (Supplementary Fig. S6). However, compared with the *D. oleifera* genome, the *D. lotus* genome lacked some regions on each chromosome, which may have been lost in the process of anchoring contigs to 15 pseudo-chromosomes using genetic maps. This result further demonstrated the integrity and accuracy of the *D. oleifera* genome assembly.

## Conclusions

We generated a high-quality chromosome-level draft genome of *D. oleifera* based on long reads generated by the third-generation PacBio Sequel sequencing platform. The final draft genome was ∼812.3 Mb, slightly smaller than the 868.4 Mb estimated by *k*-mer analysis. The Hi-C data were combined with the assembled draft genome to generate chromosome-length scaffolds. As a result, 15 scaffolds corresponding to the 15 chromosomes were assembled; these comprised 721.5 Mb and 332 scaffolds, with an N50 of 33.5 Mb, and accounted for 88.81% of the genome. A total of 30,530 protein-coding genes were predicted, and 93.61% (28,580 genes) of all protein-coding genes were annotated. Also, repeat sequences accounted for 54.8% of the genome, and 564 miRNAs, 507 tRNAs, 2207 rRNAs, and 803 snRNAs were identified. In addition, 171 candidate genes involved in tannin synthesis and deastringency were identified; of these, *CHS* genes were expanded in the *D. oleifera* genome compared with *D. lotus, C. sinensis*, and *V. vinifera*. Moreover, 186 positively selected genes were identified, including *CHI* gene, a key enzyme in the flavonoid-anthocyanin pathway. The divergence time between *D. oleifera* and *D. lotus* was estimated at 9.0 million years ago, and 2 WGD events occurred in the *D. oleifera* genome. The high-quality chromosomal genome assembly of *D. oleifera* will facilitate both research on the major economic traits in the genus *Diospyros* and assembly of the hexaploid persimmon genome.

## Editor's Note

Please also note that another genome assembly of *Diospyros oleifera* has been published during the data curation and acceptance of this paper [60].

## Availability of Supporting Data and Materials

Raw sequencing data are available in the NCBI Sequence Read Archive (Accessions: PRJNA532832), and assemblies, annotations, alignments, expression data, and BUSCO/CEGMA results are available from the *GigaScience* database GigaDB [61].

## Additional Files

Fig. S1. *k*-mer distribution of the *D. oleifera* genome.

Fig. S2. Tannin synthesis genes and the deastringency process in *Diospyros*.

Fig. S3. Venn diagram of gene family clusters of 5 Ericales species.

Fig. S4. Gene family expansion and contraction analysis of 12 species.

Fig. S5. Expression of *CHS* genes in different tissues of *D. oleifera*.

Fig. S6. Macrosynteny analysis between the *D. oleifera* genome and the *D. lotus* genome. a: gene density; b: LINE transposon density; c: LTR transposon density; d: DNA transposon density; and e: GC density (density is calculated in units of 500 kb).

Table S1. Sequencing data size by various sequencing platforms.

Table S2. Estimation of *D. oleifera* genome size by *k*-mer analysis.

Table S3. Chromosome lengths using HiC reads.

Table S4. Mapping rate of reads to *D. oleifera* genome assembly.

Table S5. CEGMA assessment of the *D. oleifera* genome.

Table S6. BUSCO notation assessment of the *D. oleifera* genome.

Table S7. Gene annotation of the *D. oleifera* genome via 3 methods.

Table S8. *D. oleifera* genome gene annotation statistics using different databases.

Table S9. *D. oleifera* genome non-coding RNA annotation statistics using different databases.

Table S10. Genes involved in tannin synthesis and the deastringency process in *D. oleifera*.

Table S11. Expansion and contraction of tannin synthase genes in *D. oleifera*.

Table S12. Positively selected genes in *D. oleifera*.

giz164_GIGA-D-19-00174_Original_Submission

giz164_GIGA-D-19-00174_Revision_1

giz164_GIGA-D-19-00174_Revision_2

giz164_Response_to_Reviewer_Comments_Original_Submission

giz164_Response_to_Reviewer_Comments_Revision_1

giz164_Reviewer_1_Report_Original_Submission Robert VanBuren -- 6/13/2019 Reviewed

giz164_Reviewer_2_Report_Original_Submission Manuel Spannagl -- 6/14/2019 Reviewed

giz164_Reviewer_2_Report_Revision_1 Manuel Spannagl -- 9/26/2019 Reviewed

giz164_Reviewer_2_Report_Revision_2 Manuel Spannagl -- 12/5/2019 Reviewed

giz164_Supplemental_Figures_and_Tables

## Abbreviations

4dtv: 4-fold degenerate sites; BWA: Burrows-Wheeler Aligner; BLAST: Basic Local Alignment Search Tool; bp: base pairs; BUSCO: Benchmarking Universal Single-Copy Orthologs; BWA: Burrows-Wheeler Aligner; CDS: coding sequence; CEGMA: Core Eukaryotic Gene Mapping Approach; EVM: EVidenceModeler; Gb; gigabase pairs; GC: guanine-cytosine; HMM: hidden Markov model; kb: kilobase pairs; KEGG: Kyoto Encyclopedia of Genes and Genomes; LINE: long interspersed nuclear element; LTR: long terminal repeat; Mb: megabase pairs; miRNA: microRNA; NCBI: National Center for Biotechnology Information; PacBio: Pacific Biosciences; PAML: Phylogenetic Analysis by Maximum Likelihood; PASA: Program to Assemble Spliced Alignments; PCNA: pollination-constant non-astringent; RNA-seq: RNA sequencing; rRNA: ribosomal RNA; SINE: short interspersed nuclear element; SMRT: single-molecule real-time; SNAP: Semi-HMM-based Nucleic Acid Parser; snRNA: small nuclear RNA; SOLAR: Sorting Out Local Alignment Results; TE: transposable element; TF: transcription factor; tRNA: transfer RNA; WGD: whole-genome duplication.

## Competing Interests

The authors declare that they have no competing interests.

## Funding

This work was supported by the National Key R & D Program of China (2018YFD1000606) and the Fundamental Research Funds for the Central Non-profit Research Institution of CAF (CAFYBB2017ZA005 and CAFYBB2017ZA004-3).

## Authors' Contributions

J.M.F. and F.D.L. conceived the project. W.J.H., H.W.L., and S.F.D. collected the samples, Y.J.S., P.S., and Y.N.M. conducted genome assembly and data analysis, X.Z. provided intellectual insights, and Y.J.S., P.S., and H.H.C. wrote the manuscript. All authors read and wrote part of the manuscript.
