# Supplementary material for: A high-quality chromosomal genome assembly of Diospyros oleifera Cheng
Source: Gigascience. 2020 Jan 16;9(1):giz164. doi: 10.1093/gigascience/giz164 (PMC6964648; doi:10.1093/gigascience/giz164)
Supplement: giz164_GIGA-D-19-00174_Original_Submission [file giz164_giga-d-19-00174_original_submission.pdf]

GigaScience

Genome sequence of Diospyros oleifera: the first chromosome-level draft Ebenaceae genome

--Manuscript Draft--

**Manuscript Number:**	GIGA-D-19-00174
**Full Title:**	Genome sequence of Diospyros oleifera: the first chromosome-level draft Ebenaceae genome
**Article Type:**	Data Note
**Funding Information:**	Fundamental Research Funds for the Central Non-profit Research Institution of CAF (CAFYBB2017ZA005) — Dr. Peng Sun<br>Fundamental Research Funds for the Central Non-profit Research Institution of CAF (CAFYBB2017ZA004-3) — Dr. Fangdong Li<br>National Key R &D Program of China (2018YFD1000606) — Dr. Jianmin Fu
**Abstract:**	Background<br><br>Diospyros oleifera, a member of the genus Diospyros of the family Ebenaceae, is an economically important tree. Phylogenetic analyses have indicated that D. oleifera is closely related to D. kaki, and could be used as a model plant for research on D. kaki. Therefore, development of the genomic resources of D. oleifera will facilitate auxiliary assembly of the hexaploid persimmon genome and provide insight into the mechanisms of sex determination.<br><br>Findings<br><br>The D. oleifera genome was assembled into a total of 443.6 Gb of raw reads using the Pacific Bioscience Sequel and Illumina HiSeq X Ten platforms. The final draft genome was approximately 812.3 Mb and had a high level of continuity with the 3.36 Mb N50. By using the Hi-C data and the draft genome assembly, fifteen scaffolds corresponding to the 15 chromosomes were assembled to a final size of 721.5 Mb using 332 scaffolds, accounting for 88.81% of the genome. The identified repeat sequences accounted for 54.8% of the genome. By de novo sequencing and analysis of homology with other plant species, 30,539 protein-coding genes with an average transcript size of 1,080.9 bp were annotated, of which 28,146 protein-coding genes (92.2%) had conserved functional motifs or functional terms. In addition, candidate genes involved in the synthesis of tannin and sex determination were identified.<br><br>Conclusions<br><br>We generated a high-quality chromosome-level draft genome for D. oleifera, which is the first reference genome of a member of the family Ebenaceae. This genome will facilitate the assembly of the hexaploid persimmon genome.
**Corresponding Author:**	Yujing Suo<br><br>CHINA
**Corresponding Author Secondary Information:**	
**Corresponding Author's Institution:**	
**Corresponding Author's Secondary Institution:**	
**First Author:**	Yujing Suo
**First Author Secondary Information:**	

Powered by Editorial Manager® and ProduXion Manager® from Aries Systems Corporation

Order of Authors:	Yujing Suo<br>Peng Sun<br>Weijuan Han<br>Songfeng Diao<br>Huawei Li<br>Yini Mai<br>Huihui Cheng<br>Xing Zhao<br>Fangdong Li<br>Jianmin Fu
Order of Authors Secondary Information:	
Additional Information:	
Question	Response
Are you submitting this manuscript to a special series or article collection?	No
**Experimental design and statistics**<br><br>Full details of the experimental design and statistical methods used should be given in the Methods section, as detailed in our Minimum Standards Reporting Checklist. Information essential to interpreting the data presented should be made available in the figure legends.<br><br>Have you included all the information requested in your manuscript?	Yes
**Resources**<br><br>A description of all resources used, including antibodies, cell lines, animals and software tools, with enough information to allow them to be uniquely identified, should be included in the Methods section. Authors are strongly encouraged to cite Research Resource Identifiers (RRIDs) for antibodies, model organisms and tools, where possible.<br><br>Have you included the information requested as detailed in our Minimum Standards Reporting Checklist?	Yes

**Availability of data and materials**<br><br>All datasets and code on which the conclusions of the paper rely must be either included in your submission or deposited in publicly available repositories (where available and ethically appropriate), referencing such data using a unique identifier in the references and in the "Availability of Data and Materials" section of your manuscript.<br><br>Have you have met the above requirement as detailed in our Minimum Standards Reporting Checklist?	Yes

Powered by Editorial Manager® and ProduXion Manager® from Aries Systems Corporation

Manuscript

Click here to download Manuscript Genome sequence of Diospyros oleifera.doc

Genome sequence of *Diospyros oleifera*: the first chromosome-level draft Ebenaceae genome

Yujing Suo[1,2,3,†], Peng Sun[1,2,3,†], Weijuan Han[1,2,3], Songfeng Diao[1,2,3], Huawei Li[1,2,3], Yini Mai[1,2,3], Huihui Cheng[4], Xing Zhao[4], Fangdong Li[1,2,3,*] and Jianmin Fu[1,2,3,*]

[1]Key Laboratory of Non-timber Forest Germplasm Enhancement & Utilization of State Administration of Forestry and Grassland, No.3 Weiwu Road, Jinshui District, Zhengzhou, 450003, China; [2]Non-timber Forest Research and Development Center, Chinese Academy of Forestry, No.3 Weiwu Road, Jinshui District, Zhengzhou, 450003, China; [3]National Innovation Alliance of Persimmon Industry, No.3 Weiwu Road, Jinshui District, Zhengzhou, 450003, China; [4]Novogene Bioinformatics Institute, Beijing, 100083, China

* Correspondence address. Fangdong Li, Non-timber Forest Research and Development Center, Chinese Academy of Forestry, No.3 Weiwu Road, Jinshui District, Zhengzhou, 450003, E-mail: lifangdong66@163.com; Jianmin Fu, Non-timber Forest Research and Development Center, Chinese Academy of Forestry, No.3 Weiwu Road, Jinshui District, Zhengzhou, 450003, E-mail: fjm371@163.com

[†]Contributed equally to this work.

Abstract

Background: *Diospyros oleifera*, a member of the genus *Diospyros* of the family Ebenaceae, is an economically important tree. Phylogenetic analyses have indicated that *D. oleifera* is closely related to *D. kaki*, and could be used as a model plant for research on *D. kaki*. Therefore, development of the genomic resources of *D. oleifera* will facilitate auxiliary assembly of the hexaploid persimmon genome and provide insight into the mechanisms of sex determination. **Findings:** The *D. oleifera* genome was assembled into a total of 443.6 Gb of raw reads using the Pacific Bioscience Sequel and Illumina HiSeq X Ten platforms. The final draft genome was approximately 812.3 Mb and had a high level of continuity with the 3.36 Mb N50. By using the Hi-C data and the draft genome assembly, fifteen scaffolds corresponding to the 15 chromosomes were assembled to a final size of 721.5 Mb using 332 scaffolds, accounting for 88.81% of the genome. The identified repeat sequences accounted for 54.8% of the genome. By *de novo* sequencing and analysis of homology with other plant species, 30,539 protein-coding genes with an average transcript size of 1,080.9 bp were annotated, of which 28,146 protein-coding genes (92.2%) had conserved functional motifs or functional terms. In addition, candidate genes involved in the synthesis of tannin and sex

determination were identified. **Conclusions:** We generated a high-quality chromosome-level draft genome for *D. oleifera*, which is the first reference genome of a member of the family Ebenaceae. This genome will facilitate the assembly of the hexaploid persimmon genome.

Keywords: *Diospyros oleifera*; chromosome-level genome assembly; Hi-C assembly; Tannin synthesis; sex determination genes

Data Description

Background

Diospyros, the largest genus in the family Ebenaceae, comprising more than 500 species, of which the ebony and fruit have considerable economic value. The ebony of more than 20 species of *Diospyros* (including *D. reticulata* from Africa, *D. ebenum* and *D. ferrea* from Asia) is used commercially for arts, crafts, and decorative building materials. In addition, *D. kaki*, *D. oleifera*, and *D. lotus* are important species for fruit production; indeed, *D. kaki* is one of the most widely distributed fruit trees worldwide. However, most *D. kaki* cultivars are hexaploid (2n=6x=90) or nonaploid (2n=9x=135) and its progenitor, origin, and polyploidization mechanisms are unclear, which hampers molecular breeding. *D. oleifera* is diploid (2n=2x=30) and its fruit contains large quantities of tannins, important raw materials for the production of persimmon paint (Fig. 1). Also, *D. oleifera* is frequently selected as stock for grafting of persimmon (*D. kaki*). Phylogenetic analyses based on the chloroplast genome and protein-coding, intergenic, and intron sequences have indicated that *D. oleifera* is closely related to *D. kaki* and could be used as a model plant for research on *D. kaki* [1]. Therefore, analysis of the genome of *D. oleifera* will contribute to auxiliary assembly of the hexaploid persimmon genome.

Similar to persimmon, *D. oleifera* has gynoecious, androecious, monoecious and andromonoecious sex types, making it ideal for studying the mechanism of sex determination of

persimmon. The mechanism of sex determination of *D. lotus* has been investigated; using segregating F1 sibling trees (female (XX) and male (XY) progenies), two key genes (*MeGI* and *OGI*) were identified. *OGI* is required for the formation of male flowers and encodes a small RNA that represses the expression of *MeGI* [2]. However, this mechanism of sex determination cannot explain the monoecious sex type of other *Diospyros* species. Therefore, development of the genomic resources of *D. oleifera* could provide insight into the mechanisms of sex determination of members of the genus *Diospyros*.

No genome sequence with chromosomal assembly of *Diospyros* has been reported. Here, we constructed a high-quality chromosome-level reference genome assembly for *D. oleifera* using the long reads generated by the Pacific Biosciences (PacBio) DNA sequencing platform and Hi-C data. This is the first chromosome-level reference genome of a member of the family Ebenaceae. The high quality (in terms of completeness and continuity) of the genome will facilitate both assembly of the hexaploid persimmon genome and further studies on the mechanisms of sex determination in the genus *Diospyros*.

Genomic DNA extraction, library construction, sequencing, and genome size estimation

Genomic DNA was extracted from fresh leaves of *D. oleifera* using a DNAsecure Plant Kit (Tiangen Biotech, Beijing, China). A short-read genomic library was prepared using the TruSeq DNA PCR-Free LT Library Kit (Illumina, San Diego, CA, USA). Five paired-end genomic sequence libraries with a gradient insert size of 250 to 450 bp were constructed and sequenced on the Illumina HiSeq X Ten platform. A total of 104.02 Gb of raw sequence data (119.78-fold coverage of the *D. oleifera* genome) were used for genome assembly (Supplementary Table S1).

At least 10 μg of sheared DNA was required to generate the 40 kb insert library. Preparation of the SMRT cell template involved DNA concentration, damage repair, end repair, ligation of hairpin adapters, and template purification. Subsequently, the genome was sequenced on the PacBio Sequel platform (Pacific Biosciences, Menlo Park, CA, USA). A total of 99.76 Gb of raw

sequence data (114.88-fold coverage of the *D. oleifera* genome) were used for genome assembly (Supplementary Table S1).

To produce a 10X genome library, about 1 ng of input DNA (50 kb length) was used for the GEM reaction during PCR, and 16 bp barcodes were introduced into droplets. Next, the droplets were fractured following purification of the intermediate DNA library. The library comprised 109.88 Gb (126.53-fold coverage of the *D. oleifera* genome) and was sequenced using 150 bp paired-end reads on the Illumina HiSeq X platform (Supplementary Table S1).

One Dovetail Hi-C library was prepared as described previously [3]. Briefly, for each library, chromatin was fixed in place in the nucleus using formaldehyde and then extracted. Fixed chromatin was digested with *Dpn*II, the 5′ overhangs were filled using biotinylated nucleotides, and free blunt ends were ligated. After ligation, crosslinks were reversed, and DNA was separated from protein. Purified DNA was treated to remove biotin outside of the ligated fragments, sheared to a mean fragment size of about 350 bp, and used to create sequence libraries with NEBNext Ultra enzymes (New England Biolabs, Ipswich, MA, USA) and Illumina-compatible adapters. Biotin-containing fragments were isolated using streptavidin beads before PCR enrichment of the libraries; the libraries were next sequenced on the Illumina HiSeq PE150 platform. A total of 98.24 Gb of reads was produced for the libraries. Together, these Dovetail Hi-C library reads provided 113.12-fold physical coverage of the genome (Supplementary Table S1).

High-quality paired-end reads from *D. oleifera* were used to generate 17-mer frequency information by k-mer analysis [4]. The 17-mer distribution was dependent on the characteristics of the genome and followed a Poisson distribution (Supplementary Fig. S1). We estimated the genome to be 868.41 Mb in size with a heterozygosity of 1.08% (Supplementary Table S2).

De novo* assembly of *D. oleifera

De novo assembly of the long reads generated by SMRT sequencing was performed using FALCON [5] (https://github.com/PacificBiosciences/FALCON/). Briefly, we first selected the

longest coverage of subreads as seeds for error correction. Next, the data were filtered and assembled (length_cutoff_pr = 4000, max_diff = 100, and max_cov = 100). A total of 2,986 contigs was assembled with a total length of 806.74 Mb (accounting for about 92.9% of the estimated genome), an N50 of 2.92 Mb, and a longest contig of 14.72 Mb (Table 1). The primary contigs (p-contigs) were polished using Quiver [6] by aligning SMRT reads, which produced a genome of 812.37 Mb and an N50 of 2.94 Mb. Finally, Pilon [7] was used to perform the second round of error correction with the short paired-end reads generated by the Illumina HiSeq platform, resulting in a genome of 811.09 Mb and a longest contig of 14.81 Mb (Table 1). For the scaffolding step, Long Ranger (v. 2.1.2, https://support.10xgenomics.com/genome-exome/software/pipelines/latest/installation) was applied to build scaffolds using the 10X data. FragScaff [8] (v. 1-1) was used to build superscaffolds from the barcoded sequencing reads. The final assembly contained 2,812 scaffolds and had a total length of 812.32 Mb, representing approximately 93.54% of the genome estimated by k-mer analysis. The sizes of the longest contig and scaffold were 14.82 and 17.43 Mb, respectively, and the N50s were 2.94 and 3.36 Mb, respectively (Table 1). Subsequently, the Hi-C sequencing data were aligned to the assembled scaffolds by BWA-mem [9], and the scaffolds were clustered onto chromosomes with LACHESIS (http://shendurelab.github.io/LACHESIS/). Among the 2,812 scaffolds, 332 were grouped into the 15 chromosomes (Fig. 2). The final genome was 721.45 Mb and the N50 was 33.5 Mb, accounting for 88.81% of the total genome (Supplementary Table S3, Fig. 3).

Assessment of the assembled genome

To estimate the quality of the assembled genome, the short reads were mapped back to the consensus genome using BWA; the overall mapping rate was 98.19%, suggesting that the assembly contained comprehensive genomic information (Supplementary Table S4). The completeness of gene regions was assessed using Core Eukaryotic Gene Mapping Approach (CEGMA) [10] and Benchmarking Universal Single-Copy Orthologs (BUSCO) [11] to evaluate the completeness of the assembled

genome. The CEGMA assessment showed that 215 (93.55%) of 248 core eukaryotic genes were covered (Supplementary Table S5). In contrast, 89.4% of complete BUSCOs were detected and 6.6% were missing (Supplementary Table S6), indicating that the assembled genome had a high level of completeness.

Repetitive elements identification of *D. oleifera*

The *D. oleifera* genome was subjected to annotation of repetitive sequences—transposable elements (TEs) and tandem repeats. RepeatMasker [12,13] (v. 3.3.0) was used to detect TEs in a repeat library derived from a known repeat library (Repbase, v. 15.02) and a *de novo* repeat library generated using RepeatModeler [3] (v. 1.0.5), RepeatScout [14], Piler, and LTR_FINDER [15]. RepeatProteinMask [13] was used to detect TEs in the *D. oleifera* genome by comparison with a TE database. Tandem repeats were identified using Tandem Repeats Finder [16].

Repetitive sequences comprised 54.8% of the *D. oleifera* genome, among which TEs accounted for 53.03%. The most frequently detected TEs were long terminal repeat (LTR) retrotransposons (46.73%), followed by DNA TEs (4.17%). Of the LTRs, 26.63% and 14.40% were Ty3/Gypsy and Ty1/Copia, respectively (Table 2).

Annotation of protein-coding genes

De novo, homolog-based, and RNA sequencing (RNA-seq)-based predictions were employed to annotate the protein-coding genes in the *D. oleifera* genome. The following *ab initio* gene prediction software packages were used to predict genes: Augustus [17, 18] (v. 3.0.2), Genescan [19] (v. 1.0), Geneid [20], GlimmerHMM [21] (v. 3.0.2), and SNAP [22]. The protein sequences of seven homologous species (including *Arabidopsis thaliana* and *Daucus carota*) were downloaded from Ensembl or the National Center for Biotechnology Information (NCBI) databases. Homologous sequences were aligned against the repeat-masked *D. oleifera* genome using TBLASTN [23] (E-value ≤ 1E-05). Genewise [24] (v. 2.2.0) was employed to predict gene models based on the aligned sequences. The RNA-seq data were assembled into the unique sequences of transcripts by mapping

the RNA-seq data to the *D. oleifera* genome using TopHat [25] (v. 2.0.8) and Cufflinks [26] (v. 2.1.1) (http://cufflinks.cbcb.umd.edu/) for transcript assembly. Alternatively, Trinity [27] was used to assemble the RNA-seq data, and the gene structures were improved using PASA [28] software (http://pasapipeline.github.io/). A weighted and non-redundant gene set was generated by merging all of the gene models predicted by the above three approaches with EVidenceModeler (EVM) [29]. PASA was applied to adjust the gene models generated by EVM. The final reference gene set contained 30,539 protein-coding genes with an average transcript size of 1,080.95 bp (Supplementary Table S7).

Functional annotation

Functional annotation of protein-coding genes was performed according to the best BLAST hit by BLASTP (E-value ≤ 1E-05) searching of the SwissProt, TrEMBL [30], and NCBI non-redundant (NR) protein databases. Motifs and domains were annotated by searching the Pfam, PRINTS, PROSITE, ProDom, and SMART InterPro (v. 29.0) databases using InterProScan [31] (v. 4.8). The Gene Ontology [32] term for each gene was obtained from the corresponding InterPro description. Additionally, the gene sets were mapped to Kyoto Encyclopedia of Genes and Genomes (KEGG) [33] (v. 53) pathways to identify the best match classification for each gene (BLASTp E-value ≤ 1E-05). Finally, 28,146 protein-coding genes (92.2%) had conserved functional motifs or functional terms—92% (28,098), 78.7% (24,032), and 72.5% (22,135) of the genes in NR, InterPro, and KEGG, respectively (Table 3).

Annotation of non-coding RNAs

tRNA genes were predicted using tRNAscan-SE software [34] with the default parameters. rRNAs were annotated based on their level of homology with the rRNAs of several species of higher plants (not shown) using BLASTN with an E-value of 1e-5. The miRNA and snRNA fragments were identified by searching the Rfam database (v. 11.0) using INFERNAL [35,36] software. Finally, 564

miRNAs, 507 tRNAs, 2,207 rRNAs, and 803 snRNAs were identified, which had average lengths of 114.69, 74.82, 161.40, and 111.54 bp, respectively (Supplementary Table S8).

Identification of tannin synthase genes and sex determination genes in *D. oleifera*

Given the importance of tannin production in *D. oleifera*, we further indentified genes within the tannin biosynthesis pathway, which including chorismic acid pathway, phenylpropane metabolic pathway, and flavonoid synthesis pathway (Supplementary Fig. S1). All of the synthase genes involved in the three pathways, as well as several closely related transcription factors including WD40 and WIP-ZF were identified by aligning to reference genes downloaded in NCBI (https://www.ncbi.nlm.nih.gov) or TAIR (https://www.arabidopsis.org/index.jsp) using Blastp (E-value<1e-5, identity≥50%, and coverage≥50%). A Pfam HMMER search was used to filter genes that don't contain the corresponding domain. Transcription factors (TFs) including MYB, MYC and WRKY were identified and classified into different families using the iTAK pipeline (http://bioinfo.bti.cornell.edu/cgi-bin/itak/index.cgi) . 16, 13, and 80 genes that are involved in chorismic acid pathway, phenylpropane metabolic pathway, and flavonoid synthesis pathway were identified, such as: genes encoding the Flavanone 3-hydroxylase (F3H), Anthocyanidin reductase (ANR) and Anthocyanidin synthase (ANS). Also, we detected 18 genes encoding transport proteins such as Glutathione S-transferase (GST) and Multi-drug and toxic compound extrusion transporter (MATE), which were closely related to transmembrane transport of tannin. Besides, we identified the key genes of acetaldehyde metabolism, such as *ADH*(10), *ALDH*(19), and *PDC*(5), which were related to the deastringency of persimmon (Supplementary Table S9). The tannin synthetic gene identified in this study provides the basis for molecular breeding of persimmon tannins.

It has been reported that *MeGI* and *OGI* were the key sex determinant genes in *D. lotus* [2], and *SyGI* was a type-C cytokinin response regulator as a potential sex determinant gene in the genus *Actinidia* [37]. To detect the sex determinant genes in *D. oleifera,* the genome sequences was

aligned with reference genes obtained from the NCBI (https://www.ncbi.nlm.nih.gov) or a kiwifruit database (ftp://bioinfo.bti.cornell.edu/pub/kiwifruit) using BLASTp (E-value < 1e-5, identity ≥ 30%, and coverage ≥ 60%). A Pfam HMMER search was performed to filter genes that did not contain the corresponding domain. As a result, two candidate *MeGI* homologous genes (evm.model.fragScaff_scaffold_41.132, located at Chr4; and evm.model.original_scaffold_1194.14, located at original_scaffold_1194) and one candidate *SyGI* homologous gene (evm.model.original_scaffold_171.540, located at Chr7) were identified in the *D. oleifera* genome. The functions of these genes should be the subject of further studies.

Phylogenetic tree construction and divergence time estimation

10 other sequenced plant species were used to investigate the evolution of *D.oleifera*, including 7 asterids plants (*P.veris, R.delavayi, C.sinensis, A.chinensis, D.carota, C.canephora, S.lycopersicum*) and 3 rosids plants (*A.thaliana, V.vinifera, C.melo*). Gene families were generated by OrthoMCL [38] (http://orthomcl.org/orthomcl/). First, nucleotide and protein data of 10 species were downloaded from Ensembl (Release 70) and NCBI. Before an "all against all" BLASTP (E-value ≤ 1E-07) program, the longest transcript was selected from alternative splicing transcripts belonging to one gene, and genes with ≤ 50 amino acids were removed. The alignments with high-scoring segment pairs (HSPs) were conjoined for each gene pair by solar [39]. To identify homologous gene-pairs, more than 30% coverage of the aligned regions in both homologous genes was required. Finally, the alignments were clustered into gene families using OrthoMCL with 1.5 inflation index. After clustering, 23,924 gene families and 414 single-copy orthologs were detected across *D.oleifera* and 10 other species. Then, the 414 shared single-copy orthologs were utilized to construct the phylogenetic tree. Protein sequences of these orthologs were aligned by MUSCLE [40]. Using the protein alignments, the phylogenetic tree was constructed by the ML (maximum likelihood) TREE algorithm in RAxML software [41, 42] (version 7.2.3). Then mcmctree program of

PAML (http://abacus.gene.ucl.ac.uk/software/paml.html) was applied to estimate divergence time among 14 species with main parameters burn-in=100,000, sample-number=100,000, and sample-frequency=2. 4 calibration points were selected from TimeTree website (http://www.timetree.org) as normal priors to restrain the age of the nodes. The phylogenetic tree confirmed the grouping of Angiospermae. The split of *D.oleifera* was estimated at 76.9 Mya (Fig.4).

Whole-genome duplication analysis.

We used BLASTP (E-value < 1e-5) to do homolog or paralog search with the *D.oleifera* and other genomes (*A.chinensis*, *C.canephora*, *C.sinensis*) and MCScanX [43] was used to detect syntenic blocks. Then calculate 4dtv (transversion substitutions at fourfold degenerate sites) rates for all syntenic genes to identify putative whole genome duplication or species split events in *D.oleifera*. Besides the ancient whole-genome duplication (WGD) events occurred in all dicots species: γ event (all core eudicots share an ancient WGD, 4dtv = 0.6), a second WGD event occurred in *D.oleifera* which might contribute to the divergence of *D.oleifera* and *C. sinensis* (Supplementary Fig. S2).

Conclusions

We generated a high-quality chromosome-level draft genome of *D. oleifera* based on long reads generated by the third-generation PacBio Sequel sequencing platform. The final draft genome was approximately 812.3 Mb, slightly smaller than the 868.4 Mb estimated by k-mer analysis. The Hi-C data were combined with the assembled draft genome to generate chromosome-length scaffolds. As a result, 15 scaffolds corresponding to the 15 chromosomes were assembled; these comprised 721.5 Mb and 332 scaffolds, with an N50 of 33.5 Mb, and accounted for 88.81% of the genome. In addition, 30,539 protein-coding genes with an average transcript size of 1,080.9 bp were predicted, and 92.2% (28,146 genes) of all protein-coding genes were annotated, 109 of which were related to the synthesis of tannin, 3 were related to sex determination. Also, repeat sequences accounted

for 54.8% of the genome, and 564 miRNAs, 507 tRNAs, 2,207 rRNAs, and 803 snRNAs were identified. The divergence time of *D. oleifera* was estimated at 76.9 Mya, and two WGD events occurred in *D.oleifera* genome. To our knowledge, our chromosome-level genome assembly of *D. oleifera* is the first reference genome of a member of the family Ebenaceae. The assembled genome will facilitate both research on the mechanisms of sex determination in the genus *Diospyros* and assembly of the hexaploid persimmon genome.

Availability of supporting data

The data set supporting the results of this article is available in the NCBI Sequence Read Archive [Accessions: PRJNA532832].

Additional files

Fig. S1: Tannins sythnase genes and deastringency process in *Diospyros*.

Fig. S2: Whole-genome duplication analysis of *D. oleifera* genome. Dol (*D.oleifera),* Ach *(A.chinensis)*, Cca (*C.canephora),* Csi*(C.sinensis*).

Table S1: The sequencing data size by various sequencing platform.

Table S2: Estimation of *D.oleifera* genome size by K-mer analysis.

Table S3: Chromosomes length using HiC reads.

Table S4: Mapping rate of reads to *D.oleifera* genome assembly.

Table S5: CEGMA assessment of *D.oleifera* genome.

Table S6: BUSCO notation assessment of *D.oleifera* genome.

Table S7: Gene annotation of *D.oleifera* genome via three methods.

Table S8: *D.oleifera* genome ncRNA annotation statistics used different databases.

Table S9: Genes involved in tannins sythnase genes and deastringency process in *D. oleifera*.

Abbreviations

BWA: Burrows-Wheeler Aligner; BLAST: Basic Local Alignment Search Tool; BUSCO: Benchmarking Universal Single-Copy Orthologs; NCBI: National Center for Biotechnology Information; PacBio: Pacific Biosciences; RNA-seq: RNA-sequencing; SMRT sequencing: single molecule real time sequencing; TE: transposable element; WGD: whole-genome duplication.

Competing interests

The authors declare that they have no competing interests.

Funding

This work was supported by the National Key R &D Program of China (2018YFD1000606), and the Fundamental Research Funds for the Central Non-profit Research Institution of CAF (CAFYBB2017ZA005 and CAFYBB2017ZA004-3)

Author contributions

J.M.F. and F.D.L. conceived the project. W.J.H., H.W.L. and S.F.D. collected the samples, Y.J.S., P.S. and Y.N.M. conducted genome assembly and data analysis, X.Z. provided intellectual insights, Y.J.S., P.S. and H.H.C. wrote the paper. All authors read and wrote part of the manuscript.

References

1. Fu JM, Liu HM, Hu JJ, et al. Five Complete Chloroplast Genome Sequences from *Diospyros*: Genome Organization and Comparative Analysis. PLOS ONE, 2016, 11(7):e0159566-.
2. Akagi T, Henry IM, Tao R, et al. A Y-chromosome-encoded small RNA acts as a sex determinant in persimmons. Science, 2014, 346(6209):646-650.

3. Lieberman-Aiden E, et al. Comprehensive mapping of long-range interactions reveals folding principles of the human genome. Science, 2009, 326: 289–293.
4. Liu, B. et al. Estimation of genomic characteristics by analyzing k-mer frequency in de novo genome projects. arXiv: Genomics, 2013.
5. Chin CS, Peluso P, Sedlazeck FJ, et al. Phased diploid genome assembly with single-molecule real-time sequencing. Nature Methods, 2016.
6. Chin CS, Alexander DH, Marks P, et al. Nonhybrid, finished microbial genome assemblies from long-read SMRT sequencing data. Nature Methods, 2013, 10(6):563.
7. Walker BJ, Abeel T, Shea T, et al. Pilon: An Integrated Tool for Comprehensive Microbial Variant Detection and Genome Assembly Improvement. PLOS ONE, 2014, 9.
8. Adey A, Kitzman JO, Burton JN, et al. In vitro, long-range sequence information for de novo genome assembly via transposase contiguity. Genome Research, 2014, 24(12):2041-2049.
9. Li H, and Durbin R. Fast and accurate short read alignment with Burrows-Wheeler transform. Bioinformatics, 2009.
10. Parra G, Bradnam K, Korf I. CEGMA: a pipeline to accurately annotate core genes in eukaryotic genomes. Bioinformatics, 2007, 23(9):1061-1067.
11. Simao FA, Waterhouse RM, Ioannidis P, et al. BUSCO: assessing genome assembly and annotation completeness with single-copy orthologs. Bioinformatics, 2015:btv351.
12. Smit AF, Hubley R, Green P. RepeatMasker at <http://www.repeatmasker.org/RepeatModeler.html>.
13. Chen, N. Using RepeatMasker to identify repetitive elements in genomic sequences. *Curr Protoc* Bioinformatics, 2004, Chapter 4, Unit 4.
14. Price AL, Jones NC, Pevzner PA . De novo identification of repeat families in large genomes. Bioinformatics, 2005, 21(Suppl 1):i351-i358.
15. Xu Z, Wang H. LTR_FINDER: an efficient tool for the prediction of full-length LTR

retrotransposons. Nucleic Acids Research, 2007, 35(Web Server):W265-W268.

16. Benson G. Tandem repeats finder: a program to analyze DNA sequences. Nucleic Acids Res. 1999, 27, 573-580.

17. Stanke M, Waack S. Gene prediction with a hidden Markov model and a new intron submodel. Bioinformatics, 2003, 19, ii215–25.

18. Stanke M, Sch?Ffmann O, Morgenstern B, et al. Gene prediction in eukaryotes with a generalized hidden Markov model that uses hints from external sources. Bmc Bioinformatics, 2006, 7(1):62-0.

19. Salamov, A. A. Ab initio gene finding in Drosophila genomic DNA. Genome Res, 2000, 10, 516–522.

20. Parra G, Blanco E, Guigó R. GeneID in Drosophila. Genome Research, 2000, 10(4):511.

21. Majoros WH, Pertea M , Salzberg SL . TigrScan and GlimmerHMM: two open source ab initio eukaryotic gene-finders. Bioinformatics, 2004, 20(16):2878-2879.

22. Korf I. Gene finding in novel genomes. BMC Bioinformatics, 2004, 5, 59.

23. Altschul SF, et al. Gapped BLAST and PSI-BLAST: a new generation of protein database search programs. Nucleic Acids Res, 1997, 25, 3389-402.

24. Birney E, Clamp M, Durbin R. Genewise and genomewise. Genome Res, 2004, 14, 988–995.

25. Trapnell C, Pachter L, Salzberg SL. TopHat: Discovering splice junctions with RNA-Seq. Bioinformatics, 2009, 25, 1105-11.

26. Trapnell C, Williams BA, Pertea G, et al. Transcript assembly and quantification by RNA-Seq reveals unannotated transcripts and isoform switching during cell differentiation. Nature Biotechnology, 2010, 28(5):511-515.

27. Grabherr MG, Haas BJ, Yassour M, et al. Full-length transcriptome assembly from RNA-Seq data without a reference genome. Nature Biotechnology, 2011, 29(7):644-652.

28. Haas BJ, Delcher AL, Mount SM, et al. Improving the Arabidopsis genome annotation using

maximal transcript alignment assemblies. Nucleic Acids Research, 2003, 31(19):5654-5666.

29. Haas BJ, Salzberg SL, Zhu W, et al. Automated eukaryotic gene structure annotation using EVidenceModeler and the Program to Assemble Spliced Alignments. Genome biology, 2008, 9(1).

30. Bairoch A, Apweiler R. The SWISS-PROT protein sequence database and its supplement TrEMBL in 2000. Nucleic Acids Res, 2000, 28, 45-48.

31. Mulder N, Apweiler R. InterPro and InterProScan: tools for protein sequence classification and comparison. Methods Mol Biol, 2007, 396, 59-70.

32. Ashburner M, Ball CA, Blake JA, et al. Gene ontology: tool for the unification of biology. The Gene Ontology Consortium. Nat Genet, 2000, 25, 25-29.

33. Kanehisa M, Goto S. KEGG: kyoto encyclopedia of genes and genomes. Nucleic Acids Res, 2000, 28, 27-30.

34. Lowe TM, Eddy SR. tRNAscan-SE: A Program for Improved Detection of Transfer RNA Genes in Genomic Sequence. Nucleic Acids Res, 1997, 25, 955-964.

35. Nawrocki EP, Kolbe DL, Eddy SR. Infernal 1.0: inference of RNA alignments. Bioinformatics, 2009, 25(10):1335.

36. Nawrocki EP, Eddy SR. Infernal 1.1: 100-fold faster RNA homology searches. Bioinformatics, 2013, 29(22):2933-2935.

37. Akagi T, Henry IM , Ohtani H, et al. A Y-encoded suppressor of feminization arose via lineage-specific duplication of a cytokinin response regulator in kiwifruit. The Plant Cell, 2018:tpc.00787.2017.

38. Li, L. OrthoMCL: Identification of Ortholog Groups for Eukaryotic Genomes. Genome Research, 2003, 13(9):2178-2189.

39. Yu XJ, Zheng HK, Wang J, et al. Detecting lineage-specific adaptive evolution of brain-expressed genes in human using rhesus macaque as outgroup. Genomics, 2006, 88(6):0-751.

40. Edgar RC. MUSCLE: multiple sequence alignment with high accuracy and high throughput. Nucleic Acids Res, 2004, 32: 1792-1797.

41. Stamatakis A. RAxML-VI-HPC: maximum likelihood-based phylogenetic analyses with thousands of taxa and mixed models. Bioinformatics, 2006, 22: 2688-2690.

42. Stamatakis A, Hoover P, Rougemont J. A Rapid Bootstrap Algorithm for the RAxML Web Servers. Systematic Biology, 2017, 57(5):758-771.

43. Wang Y, Tang H, Debarry JD, et al. MCScanX: a toolkit for detection and evolutionary analysis of gene synteny and collinearity. Nucleic Acids Research, 2012, 40(7):e49-e49.

 Click here to download Table Tables.xlsx

Table 1 Summary of the *D.oleifera* genome assembly using Pacbio long-read, Illumina reads and 10X genomics data

**Program**	**SampleID**	**Length**		**Number**	
		**Contig(bp)**	**Scaffold(bp)**	**Contig**	**Scaffold**
**Falcon**	Total	806,744,914	-	2,986	-
	N50	2,916,360	-	72	-
**Quiver**	Total	812369941	-	2,986	-
	N50	2,938,972	-	72	-
**Pilon**	Total	811,094,501	-	2986	-
	N50	2,937,127	-	72	-
**10X**	Total	811,094,501	812,323,628	2,986	2,812
	Max	14,814,786	17,432,797	-	-
	Number>=2000	-	-	2,803	2,629
	N50	2,937,127	3,359,874	71	62
	N60	2,314,962	2,662,781	103	89
	N70	1,622,862	1,911,995	144	125
	N80	790,034	1,007,083	214	182
	N90	196,816	257,477	421	333

Table 2 Classification of repetitive elements in *D.oleifera* genome

total repeative elements

Program	Repeat Size(bp)	% of genome
Trf	79,886,467	9.83
Repeatmasker	408,623,327	50.3
Proteinmask	22,154,795	2.73
Total	445,187,963	54.8

Transposons elements

Type		Transposons Elementss Length(bp)	% in Genome
DNA		33,844,732	4.17
LINE		13,187,364	1.62
SINE		74,819	0.01
LTR	total	379,582,766	46.73
	Gypsy	216,328,284	26.63
	Copia	116,970,626	14.40
	other	46,283,856	5.70
Unknown		10,513,280	1.29
Total		430,778,122	53.03

Table 3 *D.oleifera* genome gene annotation statistics used different databases

**Database**		**Annotated Number**	**Annotated Percent(%)**
Swissprot		22,135	72.5
NR		28,098	92
KEGG		21,739	71.2
InterPro	All	24,032	78.7
	GO	15,725	51.5
	Pfam	22,172	72.6
Annotated		28,146	92.2
Total		30,539	-

Figure

Click here to download Figure Fig. 1.pdf

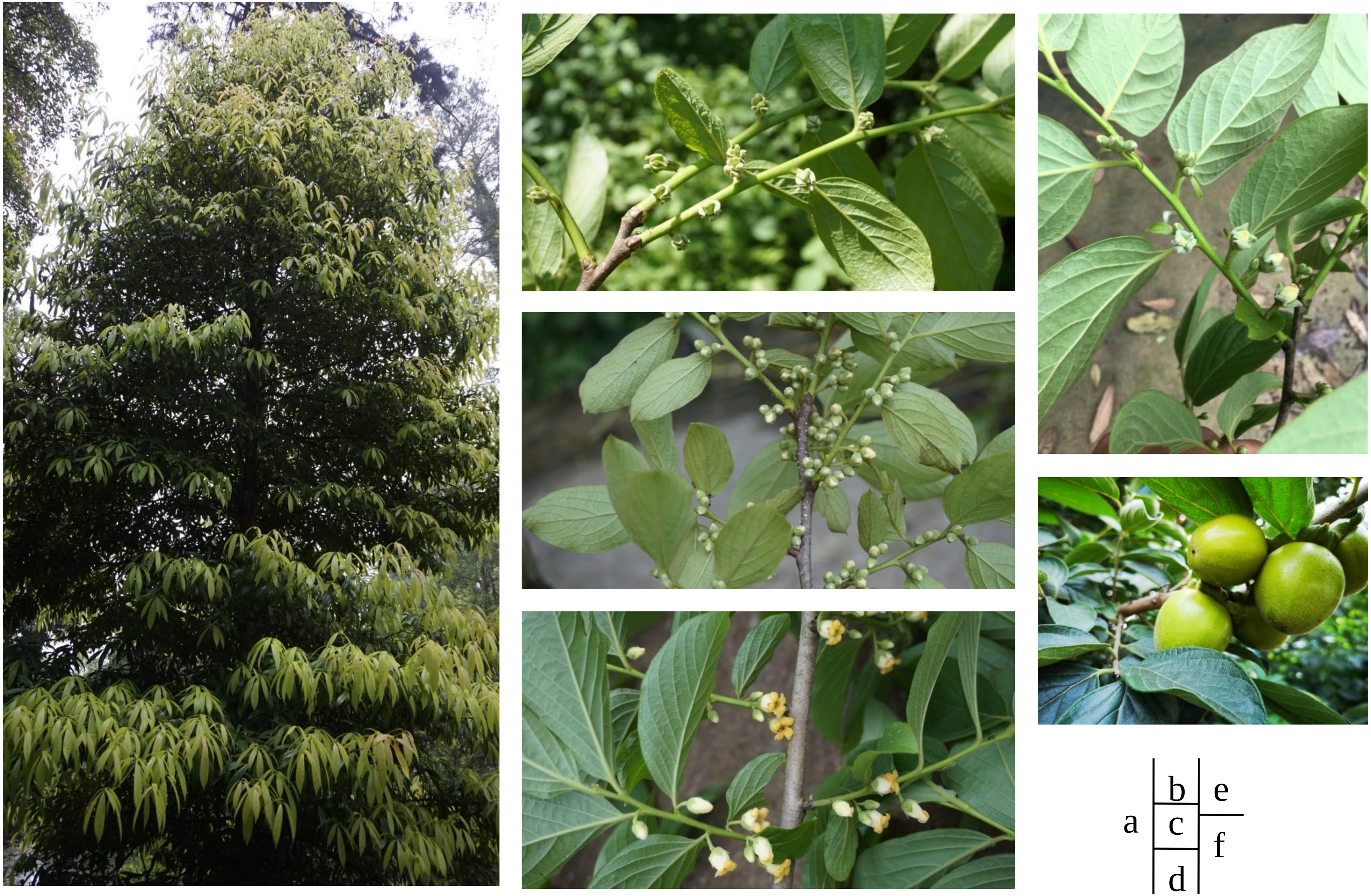

Figure

Click here to download Figure Fig. 2.pdf

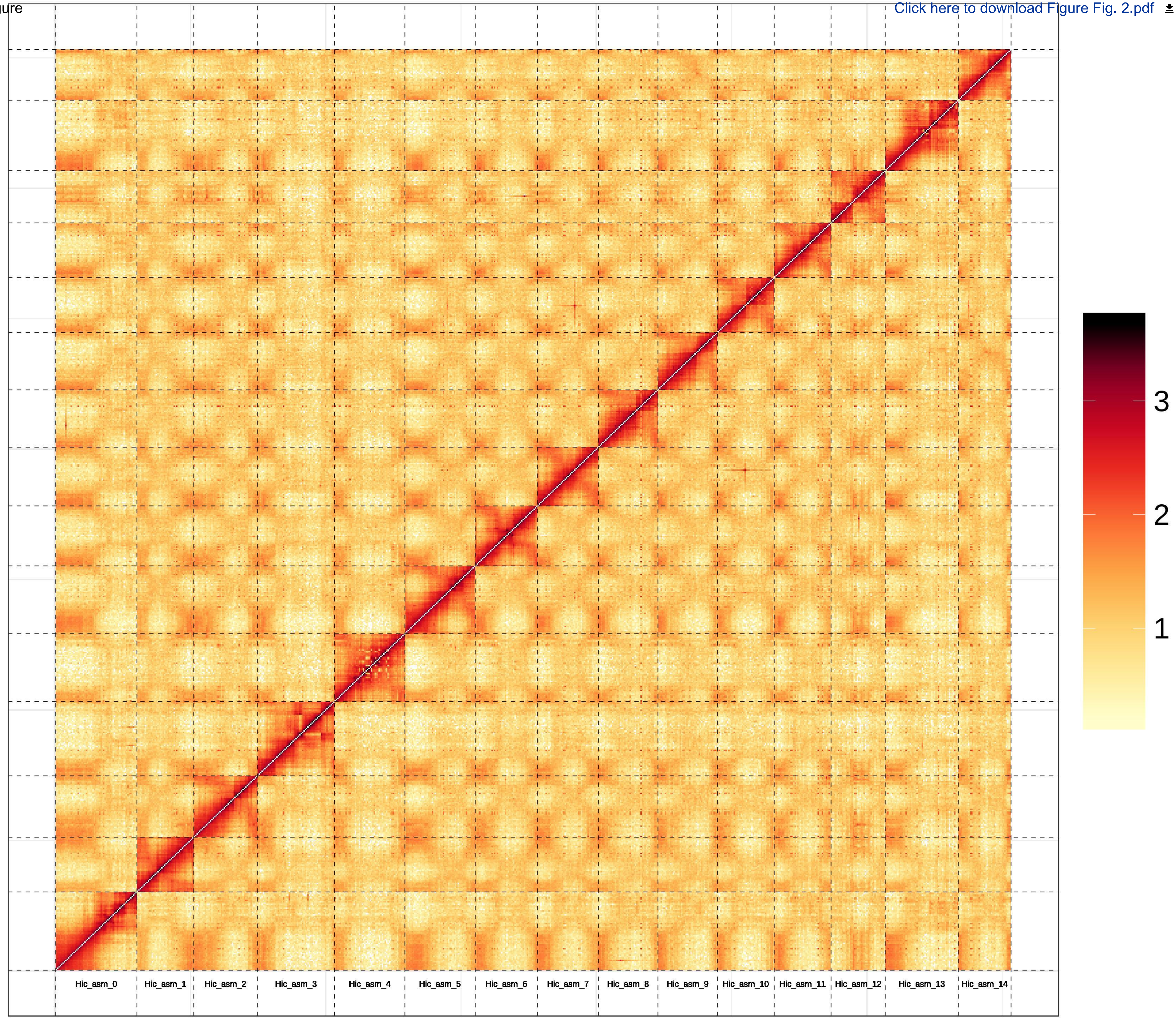

Figure

Click here to download Figure Fig. 3.png

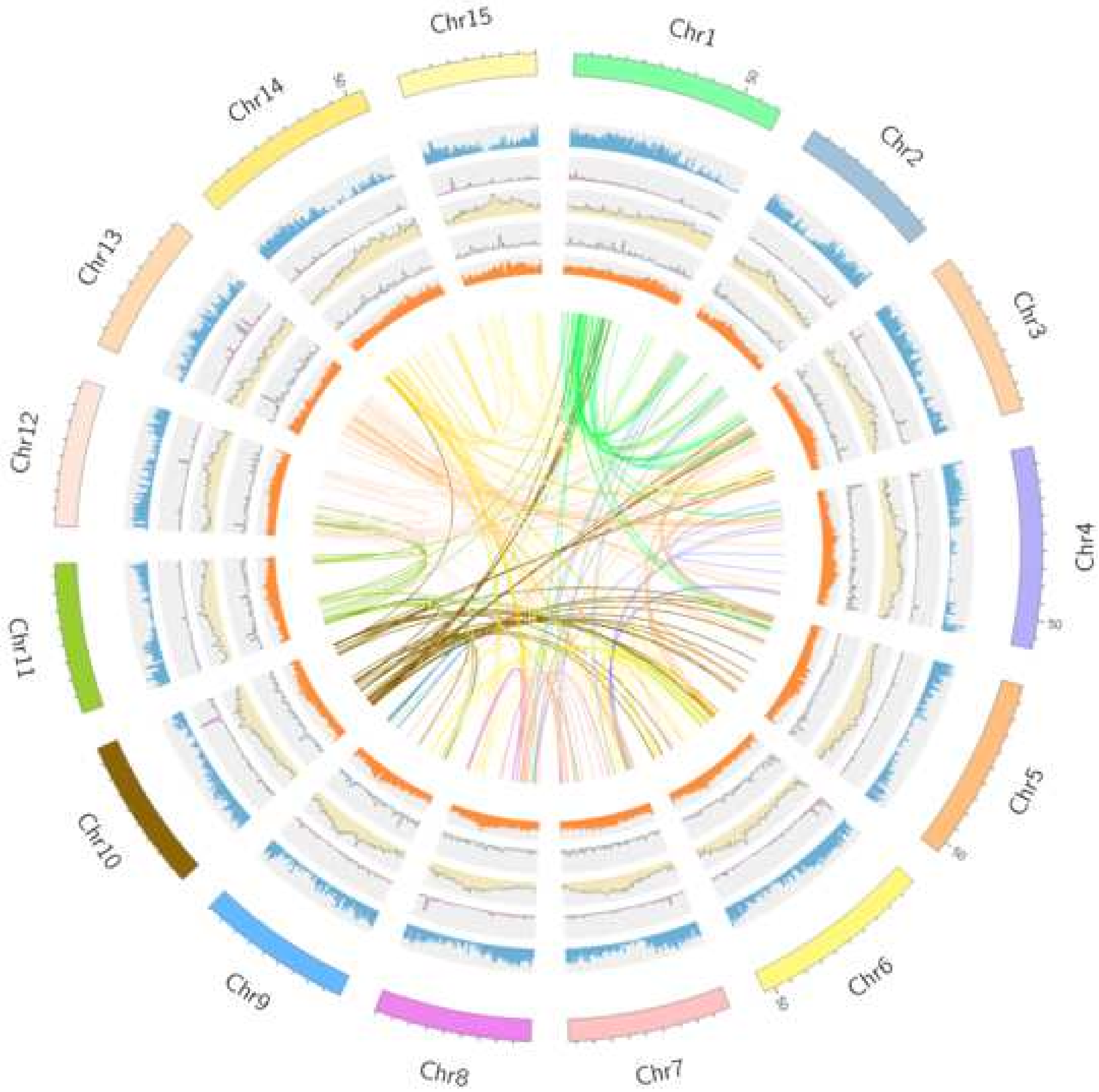

Figure

Click here to download Figure Fig. 4.png

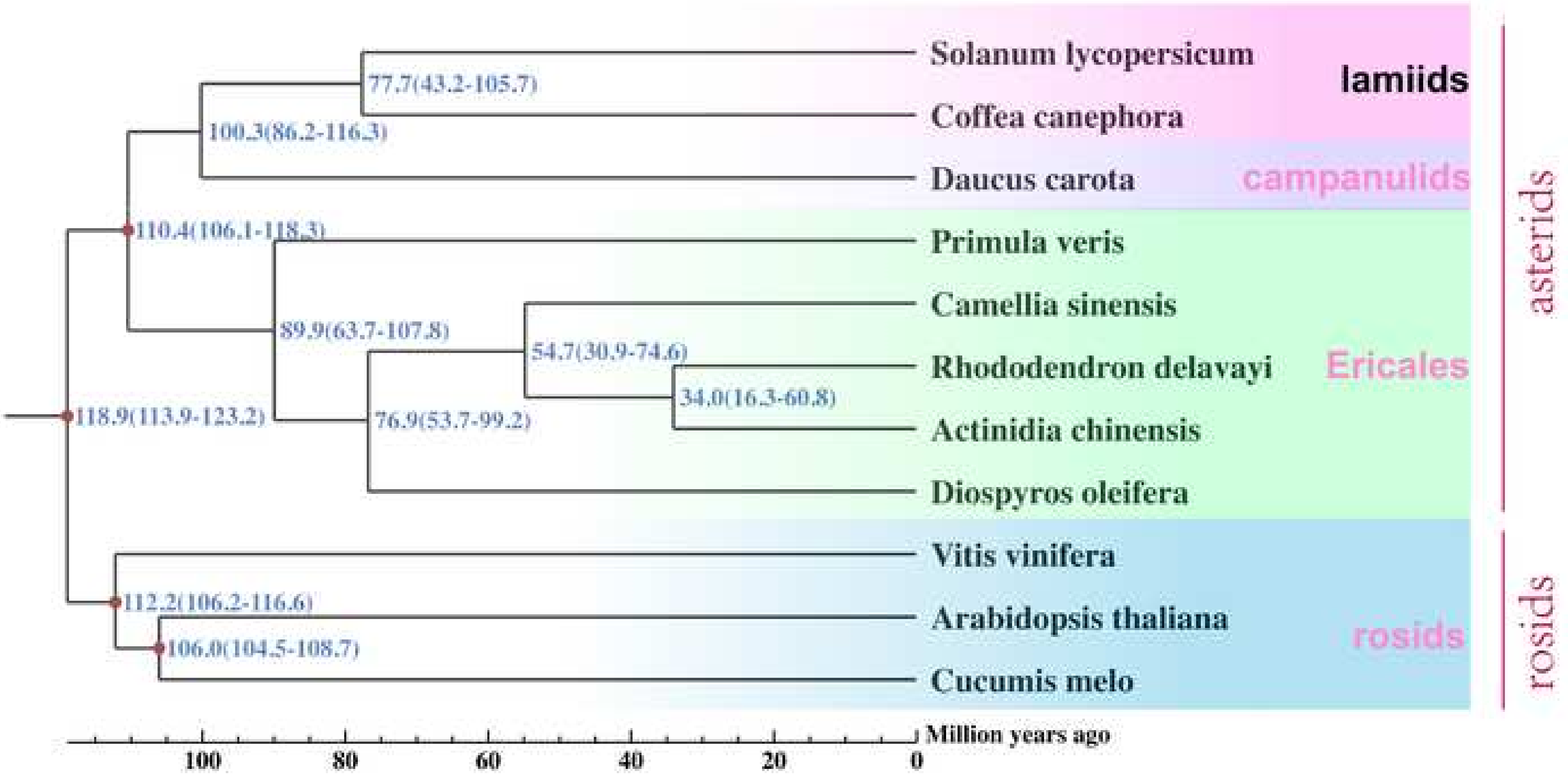

Supplementary Figure

Click here to access/download
Supplementary Material
Fig. S1.png

Supplementary Figure

Click here to access/download
Supplementary Material
Fig. S2.png

Click here to access/download
Supplementary Material
Supplementary Tables.xlsx
