# Supplementary material for: A high-quality chromosomal genome assembly of Diospyros oleifera Cheng
Source: Gigascience. 2020 Jan 16;9(1):giz164. doi: 10.1093/gigascience/giz164 (PMC6964648; doi:10.1093/gigascience/giz164)
Supplement: giz164_GIGA-D-19-00174_Revision_1 [file giz164_giga-d-19-00174_revision_1.pdf]

GigaScience

A high-quality chromosomal genome assembly of Diospyros oleifera

--Manuscript Draft--

**Manuscript Number:**	GIGA-D-19-00174R1
**Full Title:**	A high-quality chromosomal genome assembly of Diospyros oleifera
**Article Type:**	Data Note
**Funding Information:**	National Key R &D Program of China (2018YFD1000606) — Dr. Jianmin Fu<br>Fundamental Research Funds for the Central Non-profit Research Institution of CAF (CAFYBB2017ZA005) — Dr. Peng Sun<br>Fundamental Research Funds for the Central Non-profit Research Institution of CAF (CAFYBB2017ZA004-3) — Dr. Fangdong Li
**Abstract:**	Background: Diospyros oleifera, a member of the genus Diospyros of the family Ebenaceae, is an economically important tree. Phylogenetic analyses have indicated that D. oleifera is closely related to D. kaki, and could be used as a model plant for research on D. kaki. Therefore, development of the genomic resources of D. oleifera will facilitate auxiliary assembly of the hexaploid persimmon genome and provide insight into the molecular mechanisms of the major economic traits. Findings: The D. oleifera genome was assembled into a total of 443.6 Gb of raw reads using the Pacific Bioscience Sequel and Illumina HiSeq X Ten platforms. The final draft genome was approximately 812.3 Mb and had a high level of continuity with the 3.36 Mb N50. By using the Hi-C data and the draft genome assembly, fifteen scaffolds corresponding to the 15 chromosomes were assembled to a final size of 721.5 Mb using 332 scaffolds, accounting for 88.81% of the genome. The identified repeat sequences accounted for 54.8% of the genome. By de novo sequencing and analysis of homology with other plant species, 30,539 protein-coding genes with an average transcript size of 1,080.9 bp were annotated, of which 28,146 protein-coding genes (92.2%) had conserved functional motifs or functional terms. In addition, 171 candidate genes involved in the synthesis of tannin and deastringency in persimmon were identified, of which CHS (Chalcone synthase) genes had different degrees of expansion in D.oleifera genome compared with D.lotus, C.sinensis, and V.vinifera. Moreover, 186 positively selected genes were identified, including CHI (Chalcone isomerase) gene, which was one of the key enzymes in the flavonoid-anthocyanin pathway. Phylogenetic tree indicated that the split of D.oleifera and D. lotus was estimated at 9.0 Mya. Besides the ancient γ event, a second WGD event occurred in D.oleifera and D.lotus (4dtv=0.36 ~0.27-0.42). Conclusions: We generated a high-quality chromosome-level draft genome for D. oleifera, which will facilitate the assembly of the hexaploid persimmon genome and further studies on the major economic traits in the genus Diospyros.
**Corresponding Author:**	Yujing Suo<br><br>CHINA
**Corresponding Author Secondary Information:**	
**Corresponding Author's Institution:**	
**Corresponding Author's Secondary Institution:**	
**First Author:**	Yujing Suo
**First Author Secondary Information:**	
**Order of Authors:**	Yujing Suo<br>Peng Sun

Powered by Editorial Manager® and ProduXion Manager® from Aries Systems Corporation

	Huihui Cheng
	Weijuan Han
	Songfeng Diao
	Huawei Li
	Yini Mai
	Xing Zhao
	Fangdong Li
	Jianmin Fu
**Order of Authors Secondary Information:**	
**Response to Reviewers:**	Dear Editor,<br><br>Thank you very much for offering us the opportunity to resubmit a revised version of our manuscript. Hereby, we submit the revised manuscript entitled “A high-quality chromosomal genome assembly of Diospyros oleifera” (GIGA-D-19-00174) to GigaScience. We appreciate the valuable comments and suggestions from you and the reviewers, which help us to improve and clarify the manuscript. We have discussed the comments carefully and tried our best to improve the manuscript according to the reviewers’ good comments.<br><br>The main modifications are as follows:<br>1. We confirmed the NCBI data link, you and the reviewers can find the data under this link:<br>https://dataview.ncbi.nlm.nih.gov/object/PRJNA532832?reviewer=gbss3pp9p06h5hosks3vrepirg<br><br>2. We supplemented the content of comparative genomic analysis, including gene family cluster, expansion and contraction of gene families, positively selection analysis, and macrosynteny analysis. Some interesting results were found: Firstly, compared with D.lotus, C.sinensis, and V.vinifera, CHS (Chalcone synthase) genes (the first key enzyme in the flavonoid-anthocyanin pathway) had different degrees of expansion in D.oleifera genome, which may be contribute to the abundant tannin production in the D.oleifera. Besides, CHI (Chalcone isomerase) gene (another key enzyme in the flavonoid-anthocyanin pathway) was found to be positive selected. These results will provide important data support for the molecular mechanism analysis of the major economic traits in Diospyros. Furthermore, the divergence time of between D. oleifera and D.lotus was estimated at 9.0 Mya, and two WGD events occurred in D.oleifera genome. These results will be helpful for the further analysis of the evolution of Diospyros species, and the origin of hexaploid persimmon.<br><br>Detailed responses to your and the reviewers’ comments are provided in the next sections. We hope you and the reviewers will be satisfied with our responses to the comments and the revisions for the original manuscript. Please feel free to contact us with any questions and we are looking forward to your response.<br><br>Thanks and Best wishes!<br><br>Yours sincerely,<br>Yujing Suo and Jianmin Fu<br><br><br>Response to Reviewer:<br><br>Reviewer #1: Suo et al report a chromosome scale assembly of D. oleifera, a diploid relative of hexaploid persimmon. They used a combination of Illumina, 10x, PacBio, and HiC to generate the chromosome scale assembly D. oleifera. The inclusion of high coverage Illumina data and scaffolding with 10x likely ensured that most of the residual indels from the PacBio only assembly were corrected. The HiC contact map in Figure 2

has no obvious inversions or misplacements, suggesting the genome is well assembled. This resource will be useful for the comparative genomics and persimmon research communities. I have a few minor concerns that should be addressed before this manuscript is published.

Q1. The estimated heterozygosity of D. oleifera is quite high (1.1%) and this would have likely resulted in assembly issues related to haplotype specific contigs. How many primary and alternate contigs were assembled by FALCON?
A: There were 2,986 contigs initially assembled by FALCON (Table 1).

Q2.Akagi et al. (https://www.biorxiv.org/content/early/2019/05/05/628537.full.pdf) report a chromosome scale assembly of diploid persimmon (D. lotus). The authors could cite this preprint in their manuscript and if the genome is publicly available, survey macrosynteny.
A: Thanks for your suggestion. We have added the macrosynteny analysis (Revised manuscript，Page12，Line 291-297). The chromosome-based macrosynteny analysis revealed a striking correspondence between D.oleifera and D.lotus, for that there were totally 432 syntenic blocks showed in supplementary Fig. S5.

Q3. The identification of homologs of sex determination genes from kiwi and D. lotus is not informative, as the kiwi sex determination system is likely completely different from D. oleifera and sex chromosomes may have an independent origin in D. lotus and D. oleifera. It is fine to leave this in the paper, but the statement that candidate sex determination genes were identified should be removed from the abstract
A: Thanks for your suggestion. We have removed the content about sex determination, and focused on the analysis of genes related to tannin synthesis.

Q 4. The identification of a WGD event in D. oleifera is interesting, and figure S2 could probably be moved to the main text. Based on this figure, it looks like there could have been two WGD events in D. oleifera.
A: Thanks for your suggestion. We have moved the Fig. S2 to the main text as the new Fig. 5. Besides the ancient γ event (all core eudicots share an ancient WGD, 4dtv = 0.66), a second WGD event occurred in D.oleifera and D.lotus (4dtv=0.36 ~0.27-0.42) which might contribute to the divergence of Ebenaceae with A. chinensis and C. sinensis. (Revised manuscript，Page12，Line 289-292)

Minor
Page 6, line 152. homologous should not be use here
Versions are provided or most but not all bioinformatics software. Where appropriate, versions should be added.
A: ‘homologous’ in Page 6, line 152 was deleted and versions of bioinformatics software had been provided in the article. (Revised manuscript，Page7，Line 165-166)

Reviewer #2: This manuscript describes the assembly of the first chromosome-level genome sequence of an Ebenaceae, Diospyros oleifera. The newly generated genome sequence was analysed for TE and gene content as well as for tannin synthase and sex determination genes. A phologenetic tree was constructed for divergence time estimation.

Data preparation as well as the construction of the pseudomolecules follows established and proven protocols and the results look good to me. Same is true for the gene model prediction and TE detection.

My main issue with this study is that it is almost exclusively a description of a newly established genomic resources, with very little to no new biological insights included in this manuscript. There is a little bit on tannin synthase and sex determination but this is all based on existing knowledge and little more than a homolog search. I appreciate the generation of these novel and helpful resources but these data could/should have been used to gain more biological insights.
A：For the question you mentioned, we supplemented the content of comparative

Powered by Editorial Manager® and ProduXion Manager® from Aries Systems Corporation

	genomic analysis in the revised manuscript, including gene family cluster, expansion and contraction of gene families, positively selection analysis, and macrosynteny analysis. Some interesting results were found: Firstly，compared with D.lotus, C.sinensis, and V.vinifera, CHS (Chalcone synthase) genes (the first key enzyme in the flavonoid-anthocyanin pathway) had different degrees of expansion in D.oleifera genome, which may be contribute to the abundant tannin production in the D.oleifera. Besides, CHI (Chalcone isomerase) gene (another key enzyme in the flavonoid-anthocyanin pathway) was found to be positive selected. These results will contribute to the molecular mechanism analysis of the major economic traits in Diospyros. Furthermore，the divergence time of between D. oleifera and D.lotus was estimated at 9.0 Mya, and two WGD events occurred in D.oleifera genome. These results will be helpful for the further analysis of the evolution of Diospyros species, and the origin of hexaploid persimmon.<br>In brief, this study provides a high-quality chromosomal level assembly of D.oleifera genome, which will provide important data support for the assembly of subsequent hexaploid persimmon genomes and the molecular mechanism analysis of the major economic traits in Diospyros.<br><br>More specific issues:<br>a.)Transcriptome data: I could not find a proper description of the transcriptome data that was obviously generated with this study and used for gene prediction. This could e.g. also be used to establish transcription levels for gene or gene families of interest.<br>A：The description of the transcriptome data was displayed in the ‘Genomic RNA extraction, library construction and sequencing’ part of the article. (Revised manuscript，Page7，Line 156-160)<br><br>b.)Functional annotation: I would recommend to use more specialized tools such as AHRD or BLAST2GO instead of simple best Blast hit for the human readable descriptions.<br>A：Thanks for your suggestion. We had reannotated the gene set by BLAST2GO, as a result，19,900 genes were annotated. After combining with the earlier annotation by blastp with InterPro database, there were totally 20,826 genes that had GO annotation, account for 68.20% of the gene models. The results were showed in the article. (Revised manuscript，Page8，Line 186-191)<br><br>c.)Phylogeny: I would recommend to use OrthoFinder instead of the older OrthoMCL version for determining the orthologous groups. Also, I'm not sure about the bias possibly introduced by just picking the single copy orthologs for the construction of the phylogenetic tree.<br>A：Thanks for your suggestion. We had reanalysis the gene families with OrthoFinder and got totally 19,722 clusters which were used for the phylogeny construction. The results were displayed in the article. (Revised manuscript，Page9，Line 224-230)<br><br>d.)I could not access any data under the NCBI accession number given.<br>A：We confirmed the NCBI data link, you and the reviewers can find the data under this link:<br>https://dataview.ncbi.nlm.nih.gov/object/PRJNA532832?reviewer=gbss3pp9p06h5hosks3vrepirg<br><br>e.) Language and grammar needs improvement.<br>A：The English in this document has been checked by at least two professional editors, both native speakers of English. For a certificate, please see:<br>http://www.textcheck.com/certificate/Q48QsC
**Additional Information:**	
**Question**	**Response**
Are you submitting this manuscript to a special series or article collection?	No
**Experimental design and statistics**	Yes

Powered by Editorial Manager® and ProduXion Manager® from Aries Systems Corporation

Full details of the experimental design and statistical methods used should be given in the Methods section, as detailed in our Minimum Standards Reporting Checklist. Information essential to interpreting the data presented should be made available in the figure legends.<br><br>Have you included all the information requested in your manuscript?	
**Resources**<br><br>A description of all resources used, including antibodies, cell lines, animals and software tools, with enough information to allow them to be uniquely identified, should be included in the Methods section. Authors are strongly encouraged to cite Research Resource Identifiers (RRIDs) for antibodies, model organisms and tools, where possible.<br><br>Have you included the information requested as detailed in our Minimum Standards Reporting Checklist?	Yes
**Availability of data and materials**<br><br>All datasets and code on which the conclusions of the paper rely must be either included in your submission or deposited in publicly available repositories (where available and ethically appropriate), referencing such data using a unique identifier in the references and in the "Availability of Data and Materials" section of your manuscript.<br><br>Have you have met the above requirement as detailed in our Minimum Standards Reporting Checklist?	Yes

Manuscript
Click here to access/download;Manuscript;Revised Manuscript.doc
Click here to view linked References

A high-quality chromosomal genome assembly of *Diospyros oleifera*

Yujing Suo[1,2,3,†], Peng Sun[1,2,3,†], Huihui Cheng[4†], Weijuan Han[1,2,3], Songfeng Diao[1,2,3], Huawei Li[1,2,3], Yini Mai[1,2,3], Xing Zhao[4], Fangdong Li[1,2,3,]* and Jianmin Fu[1,2,3,]*

[1]Key Laboratory of Non-timber Forest Germplasm Enhancement & Utilization of State Administration of Forestry and Grassland, No.3 Weiwu Road, Jinshui District, Zhengzhou, 450003, China; [2]Non-timber Forest Research and Development Center, Chinese Academy of Forestry, No.3 Weiwu Road, Jinshui District, Zhengzhou, 450003, China; [3]National Innovation Alliance of Persimmon Industry, No.3 Weiwu Road, Jinshui District, Zhengzhou, 450003, China; [4]Novogene Bioinformatics Institute, Beijing, 100083, China

* Correspondence address. Fangdong Li, Non-timber Forest Research and Development Center, Chinese Academy of Forestry, No.3 Weiwu Road, Jinshui District, Zhengzhou, 450003, E-mail: lifangdong66@163.com; Jianmin Fu, Non-timber Forest Research and Development Center, Chinese Academy of Forestry, No.3 Weiwu Road, Jinshui District, Zhengzhou, 450003, E-mail: fjm371@163.com
[†]Contributed equally to this work.

Abstract

Background: *Diospyros oleifera*, a member of the genus *Diospyros* of the family Ebenaceae, is an economically important tree. Phylogenetic analyses have indicated that *D. oleifera* is closely related to *D. kaki*, and could be used as a model plant for research on *D. kaki*. Therefore, development of the genomic resources of *D. oleifera* will facilitate auxiliary assembly of the hexaploid persimmon genome and provide insight into the molecular mechanisms of the major economic traits. **Findings:** The *D. oleifera* genome was assembled into a total of 443.6 Gb of raw reads using the Pacific Bioscience Sequel and Illumina HiSeq X Ten platforms. The final draft genome was approximately 812.3 Mb and had a high level of continuity with the 3.36 Mb N50. By using the Hi-C data and the draft genome assembly, fifteen scaffolds corresponding to the 15 chromosomes were assembled to a final size of 721.5 Mb using 332 scaffolds, accounting for 88.81% of the genome. The identified repeat sequences accounted for 54.8% of the genome. By *de novo* sequencing and analysis of homology with other plant species, 30,539 protein-coding genes with an average transcript size of 1,080.9 bp were annotated, of which 28,146 protein-coding genes (92.2%) had conserved functional motifs or functional terms. In addition, 171 candidate genes

involved in the synthesis of tannin and deastringency in persimmon were identified, of which *CHS* (Chalcone synthase) genes had different degrees of expansion in *D.oleifera* genome compared with *D.lotus*, *C.sinensis*, and *V.vinifera*. Moreover, 186 positively selected genes were identified, including *CHI* (Chalcone isomerase) gene, which was one of the key enzymes in the flavonoid-anthocyanin pathway. Phylogenetic tree indicated that the split of *D.oleifera* and *D. lotus* was estimated at 9.0 Mya. Besides the ancient γ event, a second WGD event occurred in *D.oleifera* and *D.lotus* (4dtv=0.36 ~0.27-0.42). **Conclusions:** We generated a high-quality chromosome-level draft genome for *D. oleifera*, which will facilitate the assembly of the hexaploid persimmon genome and further studies on the major economic traits in the genus *Diospyros*.

Keywords: *Diospyros oleifera*; chromosome-level genome assembly; Hi-C assembly; Tannin synthesis; sex determination genes

Data Description

Background

Diospyros, the largest genus in the family Ebenaceae, comprising more than 500 species, of which the ebony and fruit have considerable economic value. The ebony of more than 20 species of *Diospyros* (including *D. reticulata* from Africa, *D. ebenum* and *D. ferrea* from Asia) is used commercially for arts, crafts, and decorative building materials. In addition, *D. kaki*, *D. oleifera*, and *D. lotus* are important species for fruit production; indeed, *D. kaki* is one of the most widely distributed fruit trees worldwide. However, most *D. kaki* cultivars are hexaploid (2n=6x=90) or nonaploid (2n=9x=135) and its progenitor, origin, and polyploidization mechanisms are unclear, which hampers molecular breeding. *D. oleifera* is diploid (2n=2x=30) and its fruit contains large quantities of tannins, important raw materials for the production of persimmon paint (Fig. 1). Also, *D. oleifera* is frequently selected as stock for grafting of persimmon (*D. kaki*). Phylogenetic

analyses based on the chloroplast genome and protein-coding, intergenic, and intron sequences have indicated that *D. oleifera* is closely related to *D. kaki* and could be used as a model plant for research on *D. kaki* [1]. Therefore, analysis of the genome of *D. oleifera* will contribute to auxiliary assembly of the hexaploid persimmon genome.

Similar to persimmon, *D. oleifera* has gynoecious, androecious, monoecious and andromonoecious sex types, making it ideal for studying the mechanism of sex determination of persimmon. The mechanism of sex determination of *D. lotus* has been investigated; using segregating F1 sibling trees (female (XX) and male (XY) progenies), two key genes (*MeGI* and *OGI*) were identified. *OGI* is required for the formation of male flowers and encodes a small RNA that represses the expression of *MeGI* [2]. However, this mechanism of sex determination cannot explain the monoecious sex type of other *Diospyros* species. Therefore, development of the genomic resources of *D. oleifera* could provide insight into the mechanisms of sex determination of members of the genus *Diospyros*.

Here, we constructed a high-quality chromosome-level reference genome assembly for *D. oleifera* using the long reads generated by the Pacific Biosciences (PacBio) DNA sequencing platform and Hi-C data. The high quality (in terms of completeness and continuity) of the genome will facilitate both assembly of the hexaploid persimmon genome and further studies on the major economic traits in the genus *Diospyros*.

Genomic DNA extraction, library construction, sequencing, and genome size estimation

Genomic DNA was extracted from fresh leaves of *D. oleifera* using a DNAsecure Plant Kit (Tiangen Biotech, Beijing, China). A short-read genomic library was prepared using the TruSeq DNA PCR-Free LT Library Kit (Illumina, San Diego, CA, USA). Five paired-end genomic sequence libraries with a gradient insert size of 250 to 450 bp were constructed and sequenced on the Illumina HiSeq X Ten platform. A total of 104.02 Gb of raw sequence data (119.78-fold coverage of the *D. oleifera* genome) were used for genome assembly (Supplementary Table S1).

At least 10 μg of sheared DNA was required to generate the 40 kb insert library. Preparation of the SMRT cell template involved DNA concentration, damage repair, end repair, ligation of hairpin adapters, and template purification. Subsequently, the genome was sequenced on the PacBio Sequel platform (Pacific Biosciences, Menlo Park, CA, USA). A total of 99.76 Gb of raw sequence data (114.88-fold coverage of the *D. oleifera* genome) were used for genome assembly (Supplementary Table S1).

To produce a 10X genome library, about 1 ng of input DNA (50 kb length) was used for the GEM reaction during PCR, and 16 bp barcodes were introduced into droplets. Next, the droplets were fractured following purification of the intermediate DNA library. The library comprised 109.88 Gb (126.53-fold coverage of the *D. oleifera* genome) and was sequenced using 150 bp paired-end reads on the Illumina HiSeq X platform (Supplementary Table S1).

One Dovetail Hi-C library was prepared as described previously [3]. Briefly, for each library, chromatin was fixed in place in the nucleus using formaldehyde and then extracted. Fixed chromatin was digested with *Dpn*II, the 5′ overhangs were filled using biotinylated nucleotides, and free blunt ends were ligated. After ligation, crosslinks were reversed, and DNA was separated from protein. Purified DNA was treated to remove biotin outside of the ligated fragments, sheared to a mean fragment size of about 350 bp, and used to create sequence libraries with NEBNext Ultra enzymes (New England Biolabs, Ipswich, MA, USA) and Illumina-compatible adapters. Biotin-containing fragments were isolated using streptavidin beads before PCR enrichment of the libraries; the libraries were next sequenced on the Illumina HiSeq PE150 platform. A total of 98.24 Gb of reads was produced for the libraries. Together, these Dovetail Hi-C library reads provided 113.12-fold physical coverage of the genome (Supplementary Table S1).

High-quality paired-end reads from *D. oleifera* were used to generate 17-mer frequency information by k-mer analysis [4]. The 17-mer distribution was dependent on the characteristics of the genome and followed a Poisson distribution (Supplementary Fig. S1). We estimated the

genome to be 868.41 Mb in size with a heterozygosity of 1.08% (Supplementary Table S2).

De novo assembly of *D. oleifera*

De novo assembly of the long reads generated by SMRT sequencing was performed using FALCON [5] (v.0.3, https://github.com/PacificBiosciences/FALCON/). Briefly, we first selected the longest coverage of subreads as seeds for error correction. Next, the data were filtered and assembled (length_cutoff_pr = 4000, max_diff = 100, and max_cov = 100). A total of 2,986 contigs was assembled with a total length of 806.74 Mb (accounting for about 92.9% of the estimated genome), an N50 of 2.92 Mb, and a longest contig of 14.72 Mb (Table 1). The primary contigs (p-contigs) were polished using Quiver [6] by aligning SMRT reads, which produced a genome of 812.37 Mb and an N50 of 2.94 Mb. Finally, Pilon [7] (v.1.22) was used to perform the second round of error correction with the short paired-end reads generated by the Illumina HiSeq platform, resulting in a genome of 811.09 Mb and a longest contig of 14.81 Mb (Table 1). For the scaffolding step, Long Ranger (v. 2.1.2, https://support.10xgenomics.com/genome-exome/software/pipelines/latest/installation) was applied to build scaffolds using the 10X data. FragScaff [8] (v. 1-1) was used to build superscaffolds from the barcoded sequencing reads. The final assembly contained 2,812 scaffolds and had a total length of 812.32 Mb, representing approximately 93.54% of the genome estimated by k-mer analysis. The sizes of the longest contig and scaffold were 14.82 and 17.43 Mb, respectively, and the N50s were 2.94 and 3.36 Mb, respectively (Table 1). Subsequently, the Hi-C sequencing data were aligned to the assembled scaffolds by BWA-mem [9] (v.0.7.8), and the scaffolds were clustered onto chromosomes with LACHESIS (http://shendurelab.github.io/LACHESIS/). Among the 2,812 scaffolds, 332 were grouped into the 15 chromosomes, with maximum and minimum lengths of 61.45 Mb and 40.21 Mb, respectively (Fig. 2). The final genome was 721.45 Mb and the N50 was 33.5 Mb, accounting for 88.81% of the total genome (Supplementary Table S3, Fig. 3). The continuity and integrity of the assembly for *D. oleifera* is significantly better than that of the published *D. lotus* genome, which

final genome was 945.63 Mb with contigs N50 0.65Mb, and 746.09 Mb (78.9%) was assembled into the 15 pseudomolecules[10].

Assessment of the assembled genome

To estimate the quality of the assembled genome, the short reads were mapped back to the consensus genome using BWA; the overall mapping rate was 98.19%, suggesting that the assembly contained comprehensive genomic information (Supplementary Table S4). The completeness of gene regions was assessed using Core Eukaryotic Gene Mapping Approach (CEGMA) [11] and Benchmarking Universal Single-Copy Orthologs (BUSCO) [12] to evaluate the completeness of the assembled genome. The CEGMA assessment showed that 215 (93.55%) of 248 core eukaryotic genes were covered (Supplementary Table S5). In contrast, 89.4% of complete BUSCOs were detected and 6.6% were missing (Supplementary Table S6), indicating that the assembled genome had a high level of completeness.

Repetitive elements identification of *D. oleifera*

The *D. oleifera* genome was subjected to annotation of repetitive sequences—transposable elements (TEs) and tandem repeats. RepeatMasker [13,14] (v.4.0.5) was used to detect TEs in a repeat library derived from a known repeat library (Repbase, v. 15.02) and a *de novo* repeat library generated using RepeatModeler [3] (v. 1.0.5), RepeatScout [15] (v.1.0.5), Piler (v.1.0), and LTR_FINDER [16] (v.1.0.7). RepeatProteinMask [14] (v.4.0.5) was used to detect TEs in the *D. oleifera* genome by comparison with a TE database. Tandem repeats were identified using Tandem Repeats Finder [17] (v.4.0.7).

Repetitive sequences comprised 54.8% of the *D. oleifera* genome, among which TEs accounted for 53.03%. The most frequently detected TEs were long terminal repeat (LTR) retrotransposons (46.73%), followed by DNA TEs (4.17%). Of the LTRs, 26.63% and 14.40% were Ty3/Gypsy and Ty1/Copia, respectively (Table 2).

Genomic RNA extraction, library construction, sequencing

For RNA-sequencing, we collected different tissues of *D.oleifera* from the same plant used for genome sequencing, including material from leaf, root, seed, stem and fruit. Total RNAs were extracted using TRIzol® Reagent (Thermo Fisher Scientific, USA) according to the manufacturer's instructions. RNA-seq was performed using an Illumina platform.

Annotation of protein-coding genes

De novo, homolog-based, and RNA sequencing (RNA-seq)-based predictions were employed to annotate the protein-coding genes in the *D. oleifera* genome. The following *ab initio* gene prediction software packages were used to predict genes: Augustus [18, 19] (v. 3.0.2), Genescan [20] (v. 1.0), Geneid [21] (v.1.4), GlimmerHMM [22] (v. 3.0.2), and SNAP [23] (2013-11-29). The protein sequences of seven species (including *Arabidopsis thaliana* and *Daucus carota*) were downloaded from Ensembl or the National Center for Biotechnology Information (NCBI) databases. Homologous sequences were aligned against the repeat-masked *D. oleifera* genome using TBLASTN [24] (v.2.2.26, E-value ≤ 1E-05). Genewise [25] (v. 2.2.0) was employed to predict gene models based on the aligned sequences. The RNA-seq data were assembled into the unique sequences of transcripts by mapping the RNA-seq data to the *D. oleifera* genome using TopHat [26] (v. 2.0.8) and Cufflinks [27] (v. 2.1.1) (http://cufflinks.cbcb.umd.edu/) for transcript assembly. Alternatively, Trinity [28] (v.2.1.1) was used to assemble the RNA-seq data, and the gene structures were improved using PASA [29] software (r20140417, http://pasapipeline.github.io/). A weighted and non-redundant gene set was generated by merging all of the gene models predicted by the above three approaches with EVidenceModeler (EVM) [30] (v.1.1.1). PASA was applied to adjust the gene models generated by EVM. The final reference gene set contained 30,539 protein-coding genes with an average transcript size of 7,112.37 bp, an average coding sequence size of 1,080.95 bp, and a mean number of exons per gene of 4.62 (Supplementary Table S7). The number of annotated genes in this genome is less than that in the *D. lotus* genome (40,532 genes).

Functional annotation

Functional annotation of protein-coding genes was performed according to the best BLAST hit by BLASTP (v.2.2.28, E-value ≤ 1E-05) searching of the SwissProt, TrEMBL [31], and NCBI non-redundant (NR) protein databases. Motifs and domains were annotated by searching the Pfam, PRINTS, PROSITE, ProDom, and SMART InterPro (v. 29.0) databases using InterProScan [32] (v. 4.8). The Gene Ontology term for each gene was annotated by Blast2GO [33]. Additionally, the gene sets were mapped to Kyoto Encyclopedia of Genes and Genomes (KEGG) [34] (v. 53) pathways to identify the best match classification for each gene (BLASTp E-value ≤ 1E-05). Finally, 28,580 protein-coding genes (93.59% of total 30,539 genes) had conserved functional motifs or functional terms—92.01% (28,098), 78.69% (24,032), and 71.18% (21,739) of the genes in NR, InterPro, and KEGG, respectively (Supplementary Table S8).

Annotation of non-coding RNAs

tRNA genes were predicted using tRNAscan-SE software [35] (v.1.4) with the default parameters. rRNAs were annotated based on their level of homology with the rRNAs of several species of higher plants (not shown) using BLASTN with an E-value of 1e-5. The miRNA and snRNA fragments were identified by searching the Rfam database (v. 11.0) using INFERNAL [36,37] (v.1.1) software. Finally, 564 miRNAs, 507 tRNAs, 2,207 rRNAs, and 803 snRNAs were identified, which had average lengths of 114.69, 74.82, 161.40, and 111.54 bp, respectively (Supplementary Table S9).

Identification of tannin synthase genes in *D. oleifera*

Given the importance of tannin production in *D. oleifera*, we further indentified genes within the tannin biosynthesis pathway, which including chorismic acid pathway, phenylpropane metabolic pathway, flavonoid-anthocyanin pathway, and proanthocyanidin specific pathway (Supplementary Fig. S1). All of the synthase genes involved in the three pathways, as well as several closely related transcription factors including WD40 and WIP-ZF were identified by aligning to reference genes downloaded in NCBI (https://www.ncbi.nlm.nih.gov) or TAIR

(https://www.arabidopsis.org/index.jsp) using Blastp (E-value<1e-5, identity≥50%, and coverage≥50%). A Pfam HMMER search was used to filter genes that don't contain the corresponding domain. Transcription factors (TFs) including MYB, MYC and WRKY were identified and classified into different families using the iTAK pipeline (v.1.7, http://bioinfo.bti.cornell.edu/cgi-bin/itak/index.cgi). As a result, 171 genes and 380 TFs were identified, of which 13, 59, and 21 genes were involved in phenylpropane metabolic pathway, flavonoid-anthocyanin pathway, and proanthocyanidin specific pathway respectively. Also, we detected 18 genes encoding transport proteins such as Glutathione S-transferase (GST) and Multi-drug and toxic compound extrusion transporter (MATE), which were closely related to transmembrane transport of tannin. Besides, we identified the key genes of acetaldehyde metabolism, such as *ADH*(10), *ALDH*(19), and *PDC*(5), which were related to the deastringency of persimmon (Supplementary Table S10). The tannin synthetic genes and TFs identified in this study provided the basis for molecular breeding of persimmon tannins.

Gene family cluster, Phylogenetic tree construction and divergence time estimation

10 other sequenced plant species were used to investigate the evolution of *D.oleifera*, including 8 asterids plants (*D. lotus*, *P.veris*, *R.delavayi*, *C.sinensis*, *A.chinensis*, *D.carota*, *C.canephora*, *S.lycopersicum*) and 3 rosids plants (*A.thaliana*, *V.vinifera*, *C.melo*). Gene families were generated by Orthofinder [38] (v.2.3.1, http://www.stevekellylab.com/software/orthofinder). First, nucleotide and protein data of 10 species were downloaded from Ensembl (Release 70) and NCBI. Before an "all against all" BLASTP (E-value ≤ 1E-07) program, the longest transcript was selected from alternative splicing transcripts belonging to one gene, and genes with ≤ 50 amino acids were removed. The alignments with high-scoring segment pairs (HSPs) were conjoined for each gene pair by solar [39]. After clustering, 19,722 gene families were detected across *D.oleifera* and 11 other species, of which 5,599 gene families and 221 single-copy orthologs were shared by 12 species.

Among the 5 Ericales species (*D.oleifera, D.lotus, A.chinensis, R.delavayi,* and *C.sinensis*), 177 gene families consisting of 312 genes were unique to *D.oleifera* (Supplementary Fig. S3), which KEGG pathway enriched in Ubiquitin mediated proteolysis, Pyruvate metabolism, Biosynthesis of amino acids, Carbon metabolism, Glycine, serine and threonine metabolism, Aminoacyl-tRNA biosynthesis, and Valine, leucine and isoleucine degradation.

Based on this ortholog set, a phylogenetic tree of the 12 plant species was constructed using Othofinder based on phylogenitic tree constructed by FastME [40] (v.2.1.5). Then mcmctree program of PAML [41] (v.4.5, http://abacus.gene.ucl.ac.uk/software/paml.html) was applied to estimate divergence time among 12 species by using the 221 shared single-copy orthologs with main parameters burn-in=100,000, sample-number=100,000, and sample-frequency=2. 4 calibration points were selected from TimeTree website (http://www.timetree.org) as normal priors to restrain the age of the nodes. The phylogenetic tree confirmed the grouping of Angiospermae. The split of *D.oleifera* and *D. lotus* was estimated at 9.0 Mya (Fig.4).

Expansion and contraction of gene families

We determined the expansion and contraction of the gene families by comparing the cluster size differences between the ancestor and each species using the CAFÉ program [42]. A random birth and death model were used to study changes of gene families along each lineage of phylogenetic tree. A probabilistic graphical model (PGM) was introduced to calculate the probability of transitions in gene family size from parent to child nodes in the phylogeny. Using conditional likelihoods as the test statistics, we calculated the corresponding p-valuein each lineage and p-value of 0.05 was used to identify families that were significantly expanded and contracted. Compared with the common ancestor of *D.oleifera* and *D. lotus*, 175 gene families (1,896 genes) have expanded in *D.oleifera* (Supplementary Fig. S4) which KEGG pathway enriched in Ubiquitin mediated proteolysis, ABC transporters, Carbon fixation in photosynthetic organisms, etc. On the contrary, 333 gene families

(1,021 genes) were contracted in *D.oleifera*, which KEGG pathway enriched in Plant−pathogen interaction, Phenylpropanoid biosynthesis, Cyanoamino acid metabolism, etc.

In addition, the reference sequences of tannin synthase genes identified in *D.oleifera* were used as a query for blast to identify the homology genes in *D.lotus*, *C.sinensis*, and *V.vinifera*. A sequence with similarity greater than cutoff (50%) and coverage greater than cutoff (50%) were selected as a preliminary candidate gene; then searching for the domain by hmmsearch, only when it in query was identical with that in subject, the candidate gene was finally retained. A fisher test (pvalue $\leq$ 0.05) was performed on the number of individual genes related to tannin synthesis in the genomes of the four species to see if the corresponding gene expanded or contracted. The results showed that compared with other species, *CHS* (Chalcone synthase) genes had different degrees of expansion in *D.oleifera* genome (Supplementary Table S11). CHS is the first key enzyme in the flavonoid-anthocyanin pathway, the expansion of *CHS* gene may be related to the abundant tannin production in the *D.oleifera*. Besides, compared with *V.vinifera*, *LAC* (Laccase) genes showed contraction in *D.oleifera*. It has been reported that LAC may be involved in the polymerization of persimmon tannin monomers, but the specific functions need to be further verified [43,44].

Positively selected genes in *D.oleifera*

The CDS alignments of 789 single-copy gene families in *D.oleifera*, *D.lotus*，*A.chinensis*, *P.veris*, *R.delavayi* and *S.lycopersicum* were generated using MUSCLE. Gblocks [45] was applied to filter poorly aligned positions and divergent regions of the CDS alignments. With the *D.oleifera* as foreground branch, positive selection sites were detected based on branch-site models [46] of PAML [41] using the CDS alignments. P-values were computed using the χ^2 statistic and adjusted by FDR method. Finally, 186 genes were positive selected in *D.oleifera* (Supplementary Table S12), which KEGG pathway enriched in Lipoic acid metabolism, One carbon pool by folate, Fatty acid degradation, Thiamine metabolism, Fatty acid elongation, Carotenoid biosynthesis, Fatty acid

metabolism, Nucleotide excision repair, Pyrimidine metabolism, Purine metabolism, Starch and sucrose metabolism. Among the 186 PSGs， *CHI* (Chalcone isomerase) gene was found to be positive selected (ID:evm.model.original_scaffold_909.101), which was one of the key enzymes in the flavonoid-anthocyanin pathway and was involved in the biosynthesis of persimmon tannins .

Whole-genome duplication and macrosynteny analysis.

We used BLASTP (E-value < 1e-5) to do homolog or paralog search with the *D.oleifera* and other genomes (*A.chinensis, C.canephora, C.sinensis*) and MCScanX (s=5, e=1e-5) [47] was used to detect syntenic blocks. Then calculate 4dtv (transversion substitutions at fourfold degenerate sites) rates for all syntenic genes to identify putative whole genome duplication or species split events in *D.oleifera*. Besides the ancient whole-genome duplication (WGD) events occurred in all dicots species: γ event (all core eudicots share an ancient WGD, 4dtv = 0.66), a second WGD event occurred in *D.oleifera* and *D.lotus* (4dtv=0.36 ~0.27-0.42) which might contribute to the divergence of Ebenaceae with *A. chinensis* and *C. sinensis* (Fig. 5). Besides, we got 431 syntenic blocks between *D.olerfera* and *D.lotus*. On the whole, except for the translocation of some loci, the sequence of genes between *D.olerfera* and *D.lotus* were relatively conservative (Supplementary Fig. S5). However, compared with *D.olerfera* genome, *D.lotus* genome lacked some regions on each chromosome, which may be lost in the process of anchoring contigs to 15 pseudo-chromosomes using genetic maps. This result further proved the integrity and accuracy of the *D.olerfera* genome assembly.

Conclusions

We generated a high-quality chromosome-level draft genome of *D. oleifera* based on long reads generated by the third-generation PacBio Sequel sequencing platform. The final draft genome was approximately 812.3 Mb, slightly smaller than the 868.4 Mb estimated by k-mer analysis. The Hi-

C data were combined with the assembled draft genome to generate chromosome-length scaffolds. As a result, 15 scaffolds corresponding to the 15 chromosomes were assembled; these comprised 721.5 Mb and 332 scaffolds, with an N50 of 33.5 Mb, and accounted for 88.81% of the genome. 30,539 protein-coding genes were predicted, and 92.2% (28,146 genes) of all protein-coding genes were annotated. Also, repeat sequences accounted for 54.8% of the genome, and 564 miRNAs, 507 tRNAs, 2,207 rRNAs, and 803 snRNAs were identified. In addition, 171 candidate genes involved in the synthesis of tannin and deastringency were identified, of which *CHS* genes had different degrees of expansion in *D.oleifera* genome compared with *D.lotus*, *C.sinensis*, and *V.vinifera*. Moreover, 186 positively selected genes were identified, including *CHI* gene, which was one of the key enzymes in the flavonoid-anthocyanin pathway. The divergence time between *D. oleifera* and *D.lotus* was estimated at 9.0 Mya, and two WGD events occurred in *D.oleifera* genome. The high-quality chromosomal genome assembly of *D.oleifera* will facilitate both research on the major economic traits in the genus *Diospyros* and assembly of the hexaploid persimmon genome.

Availability of supporting data

The data set supporting the results of this article is available in the NCBI Sequence Read Archive [Accessions: PRJNA532832].

Additional files

Fig. S1: k-mer distribution of the *D.olefera* genome.

Fig. S2: Tannins sythnase genes and deastringency process in *Diospyros*.

Fig. S3: Venn diagram of gene family cluster of five Ericales species.

Fig. S4: Gene family expansion and contraction analysis of 12 species.

Fig. S5: Macrosynteny analysis between *D.olefera* genome and *D.lotus* genome. a: gene density; b: LINE transposon density; c: LTR transposon density; d: DNA transposon density; e: GC density (density is calculated in units of 500 kb).

Table S1: The sequencing data size by various sequencing platform.

Table S2: Estimation of *D.oleifera* genome size by K-mer analysis.

Table S3: Chromosomes length using HiC reads.

Table S4: Mapping rate of reads to *D.oleifera* genome assembly.

Table S5: CEGMA assessment of *D.oleifera* genome.

Table S6: BUSCO notation assessment of *D.oleifera* genome.

Table S7: Gene annotation of *D.oleifera* genome via three methods.

Table S8: *D.oleifera* genome gene annotation statistics used different databases.

Table S9: *D.oleifera* genome ncRNA annotation statistics used different databases.

Table S10: Genes involved in tannins sythnase genes and deastringency process in *D. oleifera*.

Table S11: Expansion and contraction of tannin synthase genes in *D.oleifera*.

Table S12：Positively selected genes in *D.oleifera.*

Abbreviations

BWA: Burrows-Wheeler Aligner; BLAST: Basic Local Alignment Search Tool; BUSCO: Benchmarking Universal Single-Copy Orthologs; NCBI: National Center for Biotechnology Information; PacBio: Pacific Biosciences; RNA-seq: RNA-sequencing; SMRT sequencing: single molecule real time sequencing; TE: transposable element; WGD: whole-genome duplication.

Competing interests

The authors declare that they have no competing interests.

Funding

This work was supported by the National Key R &D Program of China (2018YFD1000606), and the Fundamental Research Funds for the Central Non-profit Research Institution of CAF (CAFYBB2017ZA005 and CAFYBB2017ZA004-3)

Author contributions

J.M.F. and F.D.L. conceived the project. W.J.H., H.W.L. and S.F.D. collected the samples, Y.J.S., P.S. and Y.N.M. conducted genome assembly and data analysis, X.Z. provided intellectual insights, Y.J.S., P.S. and H.H.C. wrote the paper. All authors read and wrote part of the manuscript.

The English in this document has been checked by at least two professional editors, both native speakers of English. For a certificate, please see: http://www.textcheck.com/certificate/Q48QsC

References

1. Fu JM, Liu HM, Hu JJ, et al. Five Complete Chloroplast Genome Sequences from *Diospyros*: Genome Organization and Comparative Analysis. PLOS ONE, 2016, 11(7):e0159566-.
2. Akagi T, Henry IM, Tao R, et al. A Y-chromosome-encoded small RNA acts as a sex determinant in persimmons. Science, 2014, 346(6209):646-650.
3. Lieberman-Aiden E, et al. Comprehensive mapping of long-range interactions reveals folding principles of the human genome. Science, 2009, 326: 289–293.
4. Liu, B. et al. Estimation of genomic characteristics by analyzing k-mer frequency in de novo genome projects. arXiv: Genomics, 2013.
5. Chin CS, Peluso P, Sedlazeck FJ, et al. Phased diploid genome assembly with single-molecule

real-time sequencing. Nature Methods, 2016.

6. Chin CS, Alexander DH, Marks P, et al. Nonhybrid, finished microbial genome assemblies from long-read SMRT sequencing data. Nature Methods, 2013, 10(6):563.
7. Walker BJ, Abeel T, Shea T, et al. Pilon: An Integrated Tool for Comprehensive Microbial Variant Detection and Genome Assembly Improvement. PLOS ONE, 2014, 9.
8. Adey A, Kitzman JO, Burton JN, et al. In vitro, long-range sequence information for de novo genome assembly via transposase contiguity. Genome Research, 2014, 24(12):2041-2049.
9. Li H, and Durbin R. Fast and accurate short read alignment with Burrows-Wheeler transform. Bioinformatics, 2009.
10. Akagi T, Shirasawa K, Nagasaki H, et al. The persimmon genome reveals clues to the evolution of a lineage-specific sex determination system in plants. bioRxiv 628537; doi: https://doi.org/10.1101/628537
11. Parra G, Bradnam K, Korf I. CEGMA: a pipeline to accurately annotate core genes in eukaryotic genomes. Bioinformatics, 2007, 23(9):1061-1067.
12. Simao FA, Waterhouse RM, Ioannidis P, et al. BUSCO: assessing genome assembly and annotation completeness with single-copy orthologs. Bioinformatics, 2015:btv351.
13. Smit AF, Hubley R, Green P. RepeatMasker at <http://www.repeatmasker.org/RepeatModeler.html>.
14. Chen, N. Using RepeatMasker to identify repetitive elements in genomic sequences. *Curr Protoc* Bioinformatics, 2004, Chapter 4, Unit 4.
15. Price AL, Jones NC, Pevzner PA . De novo identification of repeat families in large genomes. Bioinformatics, 2005, 21(Suppl 1):i351-i358.
16. Xu Z, Wang H. LTR_FINDER: an efficient tool for the prediction of full-length LTR retrotransposons. Nucleic Acids Research, 2007, 35(Web Server):W265-W268.
17. Benson G. Tandem repeats finder: a program to analyze DNA sequences. Nucleic Acids Res.

1999, 27, 573-580.
18. Stanke M, Waack S. Gene prediction with a hidden Markov model and a new intron submodel. Bioinformatics, 2003, 19, ii215–25.
19. Stanke M, Sch?Ffmann O, Morgenstern B, et al. Gene prediction in eukaryotes with a generalized hidden Markov model that uses hints from external sources. Bmc Bioinformatics, 2006, 7(1):62-0.
20. Salamov AA. Ab initio gene finding in Drosophila genomic DNA. Genome Res, 2000, 10, 516–522.
21. Parra G, Blanco E, Guigó R. GeneID in Drosophila. Genome Research, 2000, 10(4):511.
22. Majoros WH, Pertea M, Salzberg SL. TigrScan and GlimmerHMM: two open source ab initio eukaryotic gene-finders. Bioinformatics, 2004, 20(16):2878-2879.
23. Korf I. Gene finding in novel genomes. BMC Bioinformatics, 2004, 5, 59.
24. Altschul SF, et al. Gapped BLAST and PSI-BLAST: a new generation of protein database search programs. Nucleic Acids Res, 1997, 25, 3389-402.
25. Birney E, Clamp M, Durbin R. Genewise and genomewise. Genome Res, 2004, 14, 988–995.
26. Trapnell C, Pachter L, Salzberg SL. TopHat: Discovering splice junctions with RNA-Seq. Bioinformatics, 2009, 25, 1105-11.
27. Trapnell C, Williams BA, Pertea G, et al. Transcript assembly and quantification by RNA-Seq reveals unannotated transcripts and isoform switching during cell differentiation. Nature Biotechnology, 2010, 28(5):511-515.
28. Grabherr MG, Haas BJ, Yassour M, et al. Full-length transcriptome assembly from RNA-Seq data without a reference genome. Nature Biotechnology, 2011, 29(7):644-652.
29. Haas BJ, Delcher AL, Mount SM, et al. Improving the Arabidopsis genome annotation using maximal transcript alignment assemblies. Nucleic Acids Research, 2003, 31(19):5654-5666.
30. Haas BJ, Salzberg SL, Zhu W, et al. Automated eukaryotic gene structure annotation using

EVidenceModeler and the Program to Assemble Spliced Alignments. Genome biology, 2008, 9(1).

31. Bairoch A, Apweiler R. The SWISS-PROT protein sequence database and its supplement TrEMBL in 2000. Nucleic Acids Res, 2000, 28, 45-48.

32. Mulder N, Apweiler R. InterPro and InterProScan: tools for protein sequence classification and comparison. Methods Mol Biol, 2007, 396, 59-70.

33. Ashburner M, Ball CA, Blake JA, et al. Gene ontology: tool for the unification of biology. The Gene Ontology Consortium. Nat Genet, 2000, 25, 25-29.

34. Kanehisa M, Goto S. KEGG: kyoto encyclopedia of genes and genomes. Nucleic Acids Res, 2000, 28, 27-30.

35. Lowe TM, Eddy SR. tRNAscan-SE: A Program for Improved Detection of Transfer RNA Genes in Genomic Sequence. Nucleic Acids Res, 1997, 25, 955-964.

36. Nawrocki EP, Kolbe DL, Eddy SR. Infernal 1.0: inference of RNA alignments. Bioinformatics, 2009, 25(10):1335.

37. Nawrocki EP, Eddy SR. Infernal 1.1: 100-fold faster RNA homology searches. Bioinformatics, 2013, 29(22):2933-2935.

38. Emms DM, Kelly S. OrthoFinder: solving fundamental biases in whole genome comparisons dramatically improves orthogroup inference accuracy. Genome Biology, 2015, 16(1):157.

39. Yu XJ, Zheng HK, Wang J, et al. Detecting lineage-specific adaptive evolution of brain-expressed genes in human using rhesus macaque as outgroup. Genomics, 2006, 88(6):0-751.

40. Lefort V, Desper R, Gascuel O. FastME 2.0: A Comprehensive, Accurate, and Fast Distance-Based Phylogeny Inference Program: Table 1. Molecular Biology and Evolution, 2015:msv150.

41. Yang Z. PAML 4: Phylogenetic Analysis by Maximum Likelihood. Mol. Biol. Evol. 2007, 24: 1586-1591.

42. De Bie T, Cristianini N, Demuth JP, & Hahn MW. CAFE: a computational tool for the study of

gene family evolution. Bioinformatics,2006, 22(10), 1269-1271.

43. Zhao J，Pang Y，Dixon RA. The mysteries of proanthocyanidin transport and polymerization. Plant Physiology，2010,153 (2)：437－443.

44. Hu QN，Luo C，Zhang QL，Luo ZR. Isolation and characterization of a laccase gene potentially involved in proanthocyanidin polymerization in oriental persimmon（*Diospyros kaki* Thunb.）fruit. Molecular Biology Reports，2013, 40 (4)：2809－2820.

45. Castresana J. Selection of conserved blocks from multiple alignments for their use in phylogenetic analysis. Molecular Biology and Evolution,2000, 17: 540-552.

46. Zhang J, Nielsen R, Yang Z. Evaluation of an Improved Branch-Site Likelihood Method for Detecting Positive Selection at the Molecular Level. Mol Biol Evol.,2005, 22, 2472-2479 .

47. Wang Y, Tang H, Debarry JD, et al. MCScanX: a toolkit for detection and evolutionary analysis of gene synteny and collinearity. Nucleic Acids Research, 2012, 40(7):e49-e49.

Manuscript

Click here to access/download;Manuscript;Revised Manuscript with Track Changes.doc

Click here to view linked References

A high-quality chromosomal genome assembly of Genome sequence of *Diospyros oleifera*: the first chromosome-level draft Ebenaceae genome

Yujing Suo[1,2,3,†], Peng Sun[1,2,3,†], Huihui Cheng[4†], Weijuan Han[1,2,3], Songfeng Diao[1,2,3], Huawei Li[1,2,3], Yini Mai[1,2,3], Huihui Cheng[4], Xing Zhao[4], Fangdong Li[1,2,3,*] and Jianmin Fu[1,2,3,*]

[1]Key Laboratory of Non-timber Forest Germplasm Enhancement & Utilization of State Administration of Forestry and Grassland, No.3 Weiwu Road, Jinshui District, Zhengzhou, 450003, China; [2]Non-timber Forest Research and Development Center, Chinese Academy of Forestry, No.3 Weiwu Road, Jinshui District, Zhengzhou, 450003, China; [3]National Innovation Alliance of Persimmon Industry, No.3 Weiwu Road, Jinshui District, Zhengzhou, 450003, China; [4]Novogene Bioinformatics Institute, Beijing, 100083, China

* Correspondence address. Fangdong Li, Non-timber Forest Research and Development Center, Chinese Academy of Forestry, No.3 Weiwu Road, Jinshui District, Zhengzhou, 450003, E-mail: lifangdong66@163.com; Jianmin Fu, Non-timber Forest Research and Development Center, Chinese Academy of Forestry, No.3 Weiwu Road, Jinshui District, Zhengzhou, 450003, E-mail: fjm371@163.com

[†]Contributed equally to this work.

Abstract

Background: *Diospyros oleifera*, a member of the genus *Diospyros* of the family Ebenaceae, is an economically important tree. Phylogenetic analyses have indicated that *D. oleifera* is closely related to *D. kaki*, and could be used as a model plant for research on *D. kaki*. Therefore, development of the genomic resources of *D. oleifera* will facilitate auxiliary assembly of the hexaploid persimmon genome and provide insight into the molecular mechanisms of the major economic traitssex determination. **Findings:** The *D. oleifera* genome was assembled into a total of 443.6 Gb of raw reads using the Pacific Bioscience Sequel and Illumina HiSeq X Ten platforms. The final draft genome was approximately 812.3 Mb and had a high level of continuity with the 3.36 Mb N50. By using the Hi-C data and the draft genome assembly, fifteen scaffolds corresponding to the 15 chromosomes were assembled to a final size of 721.5 Mb using 332 scaffolds, accounting for 88.81% of the genome. The identified repeat sequences accounted for 54.8% of the genome. By *de novo* sequencing and analysis of homology with other plant species, 30,539 protein-coding genes with an average transcript size of 1,080.9 bp were annotated, of which 28,146 protein-coding genes

(92.2%) had conserved functional motifs or functional terms. In addition, 171 candidate genes ~~and 380 TFs~~ involved in the synthesis of tannin and deastringency in persimmon ~~and sex determination~~ were identified, of which *CHS* (Chalcone synthase) genes had different degrees of expansion in *D.oleifera* genome compared with *D.lotus*, *C.sinensis*, and *V.vinifera*. ~~In addition,~~Moreover, 186 positively selected genes were identified, including *CHI* (Chalcone isomerase) gene, which was one of the key ~~s a key~~ enzymes in the flavonoid-anthocyanin pathway. Phylogenetic tree indicated that the split of *D.oleifera* and *D. lotus* was estimated at 9.0 Mya. Besides the ancient γ event, a second WGD event occurred in *D.oleifera* and *D.lotus* (4dtv=0.36 ~0.27-0.42). **Conclusions:** We generated a high-quality chromosome-level draft genome for *D. oleifera*, which ~~is the first reference genome of a member of the family Ebenaceae. This genome~~ will facilitate the assembly of the hexaploid persimmon genome and further studies on the major economic traits in the genus *Diospyros*.

Formatted: Font: Italic

Formatted: Font: Italic

Formatted: Font: Italic

Formatted: Font: Italic

Formatted: Font: Italic

Formatted: Font: Italic

Formatted: Font: Italic

Formatted: Font: Italic

Formatted: Font: Italic

Formatted: Font: Italic

Formatted: Font: Italic

Keywords: *Diospyros oleifera*; chromosome-level genome assembly; Hi-C assembly; Tannin synthesis; sex determination genes

Data Description

Background

Diospyros, the largest genus in the family Ebenaceae, comprising more than 500 species, of which the ebony and fruit have considerable economic value. The ebony of more than 20 species of *Diospyros* (including *D. reticulata* from Africa, *D. ebenum* and *D. ferrea* from Asia) is used commercially for arts, crafts, and decorative building materials. In addition, *D. kaki*, *D. oleifera*, and *D. lotus* are important species for fruit production; indeed, *D. kaki* is one of the most widely distributed fruit trees worldwide. However, most *D. kaki* cultivars are hexaploid (2n=6x=90) or nonaploid (2n=9x=135) and its progenitor, origin, and polyploidization mechanisms are unclear,

which hampers molecular breeding. *D. oleifera* is diploid (2n=2x=30) and its fruit contains large quantities of tannins, important raw materials for the production of persimmon paint (Fig. 1). Also, *D. oleifera* is frequently selected as stock for grafting of persimmon (*D. kaki*). Phylogenetic analyses based on the chloroplast genome and protein-coding, intergenic, and intron sequences have indicated that *D. oleifera* is closely related to *D. kaki* and could be used as a model plant for research on *D. kaki* [1]. Therefore, analysis of the genome of *D. oleifera* will contribute to auxiliary assembly of the hexaploid persimmon genome.

Similar to persimmon, *D. oleifera* has gynoecious, androecious, monoecious and andromonoecious sex types, making it ideal for studying the mechanism of sex determination of persimmon. The mechanism of sex determination of *D. lotus* has been investigated; using segregating F1 sibling trees (female (XX) and male (XY) progenies), two key genes (*MeGI* and *OGI*) were identified. *OGI* is required for the formation of male flowers and encodes a small RNA that represses the expression of *MeGI* [2]. However, this mechanism of sex determination cannot explain the monoecious sex type of other *Diospyros* species. Therefore, development of the genomic resources of *D. oleifera* could provide insight into the mechanisms of sex determination of members of the genus *Diospyros*.

~~No genome sequence with chromosomal assembly of *Diospyros* has been reported.~~ Here, we constructed a high-quality chromosome-level reference genome assembly for *D. oleifera* using the long reads generated by the Pacific Biosciences (PacBio) DNA sequencing platform and Hi-C data. ~~This is the first chromosome-level reference genome of a member of the family Ebenaceae.~~ The high quality (in terms of completeness and continuity) of the genome will facilitate both assembly of the hexaploid persimmon genome and further studies on the major economic traits ~~mechanisms of sex determination~~ in the genus *Diospyros*.

Genomic DNA extraction, library construction, sequencing, and genome size estimation

Genomic DNA was extracted from fresh leaves of *D. oleifera* using a DNAsecure Plant Kit

(Tiangen Biotech, Beijing, China). A short-read genomic library was prepared using the TruSeq DNA PCR-Free LT Library Kit (Illumina, San Diego, CA, USA). Five paired-end genomic sequence libraries with a gradient insert size of 250 to 450 bp were constructed and sequenced on the Illumina HiSeq X Ten platform. A total of 104.02 Gb of raw sequence data (119.78-fold coverage of the *D. oleifera* genome) were used for genome assembly (Supplementary Table S1).

At least 10 μg of sheared DNA was required to generate the 40 kb insert library. Preparation of the SMRT cell template involved DNA concentration, damage repair, end repair, ligation of hairpin adapters, and template purification. Subsequently, the genome was sequenced on the PacBio Sequel platform (Pacific Biosciences, Menlo Park, CA, USA). A total of 99.76 Gb of raw sequence data (114.88-fold coverage of the *D. oleifera* genome) were used for genome assembly (Supplementary Table S1).

To produce a 10X genome library, about 1 ng of input DNA (50 kb length) was used for the GEM reaction during PCR, and 16 bp barcodes were introduced into droplets. Next, the droplets were fractured following purification of the intermediate DNA library. The library comprised 109.88 Gb (126.53-fold coverage of the *D. oleifera* genome) and was sequenced using 150 bp paired-end reads on the Illumina HiSeq X platform (Supplementary Table S1).

One Dovetail Hi-C library was prepared as described previously [3]. Briefly, for each library, chromatin was fixed in place in the nucleus using formaldehyde and then extracted. Fixed chromatin was digested with *Dpn*II, the 5′ overhangs were filled using biotinylated nucleotides, and free blunt ends were ligated. After ligation, crosslinks were reversed, and DNA was separated from protein. Purified DNA was treated to remove biotin outside of the ligated fragments, sheared to a mean fragment size of about 350 bp, and used to create sequence libraries with NEBNext Ultra enzymes (New England Biolabs, Ipswich, MA, USA) and Illumina-compatible adapters. Biotin-containing fragments were isolated using streptavidin beads before PCR enrichment of the libraries; the libraries were next sequenced on the Illumina HiSeq PE150 platform. A total of

98.24 Gb of reads was produced for the libraries. Together, these Dovetail Hi-C library reads provided 113.12-fold physical coverage of the genome (Supplementary Table S1).

High-quality paired-end reads from *D. oleifera* were used to generate 17-mer frequency information by k-mer analysis [4]. The 17-mer distribution was dependent on the characteristics of the genome and followed a Poisson distribution (Supplementary Fig. S1). We estimated the genome to be 868.41 Mb in size with a heterozygosity of 1.08% (Supplementary Table S2).

De novo* assembly of *D. oleifera

De novo assembly of the long reads generated by SMRT sequencing was performed using FALCON [5] (v.0.3, https://github.com/PacificBiosciences/FALCON/). Briefly, we first selected the longest coverage of subreads as seeds for error correction. Next, the data were filtered and assembled (length_cutoff_pr = 4000, max_diff = 100, and max_cov = 100). A total of 2,986 contigs was assembled with a total length of 806.74 Mb (accounting for about 92.9% of the estimated genome), an N50 of 2.92 Mb, and a longest contig of 14.72 Mb (Table 1). The primary contigs (p-contigs) were polished using Quiver [6] by aligning SMRT reads, which produced a genome of 812.37 Mb and an N50 of 2.94 Mb. Finally, Pilon [7] (v.1.22) was used to perform the second round of error correction with the short paired-end reads generated by the Illumina HiSeq platform, resulting in a genome of 811.09 Mb and a longest contig of 14.81 Mb (Table 1). For the scaffolding step, Long Ranger (v. 2.1.2, https://support.10xgenomics.com/genome-exome/software/pipelines/latest/installation) was applied to build scaffolds using the 10X data. FragScaff [8] (v. 1-1) was used to build superscaffolds from the barcoded sequencing reads. The final assembly contained 2,812 scaffolds and had a total length of 812.32 Mb, representing approximately 93.54% of the genome estimated by k-mer analysis. The sizes of the longest contig and scaffold were 14.82 and 17.43 Mb, respectively, and the N50s were 2.94 and 3.36 Mb, respectively (Table 1). Subsequently, the Hi-C sequencing data were aligned to the assembled scaffolds by BWA-mem [9] (v.0.7.8), and the scaffolds were clustered onto chromosomes with

LACHESIS (http://shendurelab.github.io/LACHESIS/). Among the 2,812 scaffolds, 332 were grouped into the 15 chromosomes, with maximum and minimum lengths of 61.45 Mb and 40.21 Mb, respectively (Fig. 2). The final genome was 721.45 Mb and the N50 was 33.5 Mb, accounting for 88.81% of the total genome (Supplementary Table S3, Fig. 3). The continuity and integrity of the assembly for *D. oleifera* is significantly better than th~~ose~~at of the published *D. lotus* genome~~.~~, which final genome was 945.63 Mb with contigs N50 0.65 Mb, and 746.09 Mb (78.9%) was assembled into the 15 pseudomolecules[10]~~()~~.

Formatted: Font: Italic

Formatted: Font: Italic

Assessment of the assembled genome

To estimate the quality of the assembled genome, the short reads were mapped back to the consensus genome using BWA; the overall mapping rate was 98.19%, suggesting that the assembly contained comprehensive genomic information (Supplementary Table S4). The completeness of gene regions was assessed using Core Eukaryotic Gene Mapping Approach (CEGMA) [~~10~~11] and Benchmarking Universal Single-Copy Orthologs (BUSCO) [~~11~~12] to evaluate the completeness of the assembled genome. The CEGMA assessment showed that 215 (93.55%) of 248 core eukaryotic genes were covered (Supplementary Table S5). In contrast, 89.4% of complete BUSCOs were detected and 6.6% were missing (Supplementary Table S6), indicating that the assembled genome had a high level of completeness.

Repetitive elements identification of *D. oleifera*

The *D. oleifera* genome was subjected to annotation of repetitive sequences—transposable elements (TEs) and tandem repeats. RepeatMasker [~~12~~13,~~13~~14] (v.~~ 3.3.0~~4.0.5) was used to detect TEs in a repeat library derived from a known repeat library (Repbase, v. 15.02) and a *de novo* repeat library generated using RepeatModeler [3] (v. 1.0.5), RepeatScout [~~14~~15] (v.1.0.5), Piler (v.1.0), and LTR_FINDER [~~15~~16] (v.1.0.7). RepeatProteinMask [~~13~~14] (v.4.0.5) was used to detect TEs in the *D. oleifera* genome by comparison with a TE database. Tandem repeats were identified using Tandem Repeats Finder [~~16~~17] (v.4.0.7).

Repetitive sequences comprised 54.8% of the *D. oleifera* genome, among which TEs accounted for 53.03%. The most frequently detected TEs were long terminal repeat (LTR) retrotransposons (46.73%), followed by DNA TEs (4.17%). Of the LTRs, 26.63% and 14.40% were Ty3/Gypsy and Ty1/Copia, respectively (Table 2).

Genomic RNA extraction, library construction, sequencing

For RNA-sequencing, we collected different tissues of *D.oleifera* from the same plant used for genome sequencing, including material from leaf, root, seed, stem and fruit. Total RNAs were extracted using TRIzol® Reagent (Thermo Fisher Scientific, USA) according to the manufacturer's instructions. RNA-seq was performed using an Illumina platform.

Annotation of protein-coding genes

De novo, homolog-based, and RNA sequencing (RNA-seq)-based predictions were employed to annotate the protein-coding genes in the *D. oleifera* genome. The following *ab initio* gene prediction software packages were used to predict genes: Augustus [~~17~~18, ~~18~~19] (v. 3.0.2), Genescan [~~19~~20] (v. 1.0), Geneid [~~20~~21] (v.1.4), GlimmerHMM [~~21~~22] (v. 3.0.2), and SNAP [~~22~~23] (2013-11-29). The protein sequences of seven ~~homologous~~ species (including *Arabidopsis thaliana* and *Daucus carota*) were downloaded from Ensembl or the National Center for Biotechnology Information (NCBI) databases. Homologous sequences were aligned against the repeat-masked *D. oleifera* genome using TBLASTN [~~23~~24] (v.2.2.26, E-value ≤ 1E-05). Genewise [~~24~~25] (v. 2.2.0) was employed to predict gene models based on the aligned sequences. The RNA-seq data were assembled into the unique sequences of transcripts by mapping the RNA-seq data to the *D. oleifera* genome using TopHat [~~25~~26] (v. 2.0.8) and Cufflinks [~~26~~27] (v. 2.1.1) (http://cufflinks.cbcb.umd.edu/) for transcript assembly. Alternatively, Trinity [~~27~~28] (v.2.1.1) was used to assemble the RNA-seq data, and the gene structures were improved using PASA [~~28~~29] software (r20140417, http://pasapipeline.github.io/). A weighted and non-redundant gene set was generated by merging all of the gene models predicted by the above three approaches with EVidenceModeler (EVM) [~~29~~30]

Formatted: Font: Bold

Formatted: Indent: First line: 0"

Formatted: Font: Italic

Formatted: Font:

(v.1.1.1). PASA was applied to adjust the gene models generated by EVM. The final reference gene set contained 30,539 protein-coding genes with an average transcript size of ~~1,080.95~~7,112.37 bp, an average coding sequence size of 1,080.95 bp, and a mean number of exons per gene of 4.62 (Supplementary Table S7). The number of annotated genes in this genome is ~~more~~less ~~in this genome~~ than that in the *D. lotus* genome (40,532 genes).

Formatted: Font: Italic

Functional annotation

Functional annotation of protein-coding genes was performed according to the best BLAST hit by BLASTP (v.2.2.28, E-value ≤ 1E-05) searching of the SwissProt, TrEMBL [~~30~~31], and NCBI non-redundant (NR) protein databases. Motifs and domains were annotated by searching the Pfam, PRINTS, PROSITE, ProDom, and SMART InterPro (v. 29.0) databases using InterProScan [~~31~~32] (v. 4.8). The Gene Ontology ~~[32~~33] term for each gene was annotated by Blast2GO [33].~~obtained from the corresponding InterPro description.~~ Additionally, the gene sets were mapped to Kyoto Encyclopedia of Genes and Genomes (KEGG) [~~33~~34] (v. 53) pathways to identify the best match classification for each gene (BLASTp E-value ≤ 1E-05). Finally, ~~28,146~~28,580 protein-coding genes (~~92.2~~93.59% of total 30,539 genes) had conserved functional motifs or functional terms—92.01% (28,098), 78.~~7~~69% (24,032), and ~~72.548~~71.18% (~~22,135~~21,739) of the genes in NR, InterPro, and KEGG, respectively (Supplementary Table ~~3~~S8).

Annotation of non-coding RNAs

tRNA genes were predicted using tRNAscan-SE software [~~34~~35] (v.1.4) with the default parameters. rRNAs were annotated based on their level of homology with the rRNAs of several species of higher plants (not shown) using BLASTN with an E-value of 1e-5. The miRNA and snRNA fragments were identified by searching the Rfam database (v. 11.0) using INFERNAL [~~35~~36,~~36~~37] (v.1.1) software. Finally, 564 miRNAs, 507 tRNAs, 2,207 rRNAs, and 803 snRNAs were identified, which had average lengths of 114.69, 74.82, 161.40, and 111.54 bp, respectively (Supplementary Table ~~S8~~S9).

Identification of tannin synthase genes ~~and sex determination genes~~ in *D. oleifera*

Given the importance of tannin production in *D. oleifera*, we further indentified genes within the tannin biosynthesis pathway, which including chorismic acid pathway, phenylpropane metabolic pathway, ~~and~~ flavonoid-anthocyanin ~~synthesis~~ pathway, and proanthocyanidin specific pathway (Supplementary Fig. S1). All of the synthase genes involved in the three pathways, as well as several closely related transcription factors including WD40 and WIP-ZF were identified by aligning to reference genes downloaded in NCBI (https://www.ncbi.nlm.nih.gov) or TAIR (https://www.arabidopsis.org/index.jsp) using Blastp (E-value<1e-5, identity≥50%, and coverage≥50%). A Pfam HMMER search was used to filter genes that don't contain the corresponding domain. Transcription factors (TFs) including MYB, MYC and WRKY were identified and classified into different families using the iTAK pipeline (v.1.7, http://bioinfo.bti.cornell.edu/cgi-bin/itak/index.cgi)~~–~~. As a result, 171 genes and 380 TFs were identified, of which ~~16~~13, ~~13~~59, and ~~80~~ 21 genes ~~that are~~were involved in ~~chorismic acid pathway,~~ phenylpropane metabolic pathway, ~~and~~ flavonoid-anthocyanin pathway, and proanthocyanidin specific pathway ~~flavonoid synthesis pathway were identified,~~ respectively. ~~such as: genes encoding the Flavanone 3-hydroxylase (F3H), Anthocyanidin reductase (ANR) and Anthocyanidin synthase (ANS).~~ Also, we detected 18 genes encoding transport proteins such as Glutathione S-transferase (GST) and Multi-drug and toxic compound extrusion transporter (MATE), which were closely related to transmembrane transport of tannin. Besides, we identified the key genes of acetaldehyde metabolism, such as *ADH*(10), *ALDH*(19), and *PDC*(5), which were related to the deastringency of persimmon (Supplementary Table ~~S9~~S10). The tannin synthetic genes and TFs identified in this study ~~provides~~ provided the basis for molecular breeding of persimmon tannins.

~~It has been reported that *MeGI* and *OGI* were the key sex determinant genes in *D. lotus* [2], and *SyGI* was a type-C cytokinin response regulator as a potential sex determinant gene in the genus *Actinidia* [37]. To detect the sex determinant genes in *D. oleifera*, the genome sequences was~~

Formatted: Default Paragraph Font

~~aligned with reference genes obtained from the NCBI (https://www.ncbi.nlm.nih.gov) or a kiwifruit database (ftp://bioinfo.bti.cornell.edu/pub/kiwifruit) using BLASTp (E-value < 1e-5, identity ≥ 30%, and coverage ≥ 60%). A Pfam HMMER search was performed to filter genes that did not contain the corresponding domain. As a result, two candidate *MeGI* homologous genes (evm.model.fragScaff_scaffold_41.132, located at Chr4; and evm.model.original_scaffold_1194.14, located at original_scaffold_1194) and one candidate *SyGI* homologous gene (evm.model.original_scaffold_171.540, located at Chr7) were identified in the *D. oleifera* genome. The functions of these genes should be the subject of further studies.~~

Gene family cluster, Phylogenetic tree construction and divergence time estimation

10 other sequenced plant species were used to investigate the evolution of *D.oleifera*, including ~~7~~ 8 asterids plants (*D. lotus*, *P.veris*, *R.delavayi*, *C.sinensis*, *A.chinensis*, *D.carota*, *C.canephora*, *S.lycopersicum*) and 3 rosids plants (*A.thaliana*, *V.vinifera*, *C.melo*). Gene families were generated by Orthofinder [38] (v.2.3.1, http://www.stevekellylab.com/software/orthofinder). ~~OrthoMCL [38] (http://orthomcl.org/orthomcl/).~~ First, nucleotide and protein data of 10 species were downloaded from Ensembl (Release 70) and NCBI. Before an "all against all" BLASTP (E-value ≤ 1E-07) program, the longest transcript was selected from alternative splicing transcripts belonging to one gene, and genes with ≤ 50 amino acids were removed. The alignments with high-scoring segment pairs (HSPs) were conjoined for each gene pair by solar [39]. ~~To identify homologous gene pairs, more than 30% coverage of the aligned regions in both homologous genes was required. Finally, the alignments were clustered into gene families using OrthoMCL with 1.5 inflation index.~~ After clustering, ~~23,924~~19,722 gene families were detected across *D.oleifera* and 11 other species, of which 5,599 gene families and ~~414~~ 221 single-copy orthologs were ~~detected across *D.oleifera* and 10~~ ~~11~~ ~~others~~shared by 12 species. –Among the 5 Ericales species (~~D.~~ *D.oleifera*, *D.lotus*, *A.chinensis*, *R.delavayi*, and *C.sinensis*), 177 gene families consisting of 312 genes were

Formatted: Font: Italic

Formatted: Font: Not Italic

Formatted: Font: Not Italic

unique to *D.oleifera* (Supplementary Fig. 4S3), which KEGG pathway enriched in Ubiquitin mediated proteolysis, Pyruvate metabolism, Biosynthesis of amino acids, Carbon metabolism, Glycine, serine and threonine metabolism, Aminoacyl-tRNA biosynthesis, and Valine, leucine and isoleucine degradation.

Based on this ortholog set, a phylogenetic tree of the 12 plant species was constructed using Othofinder based on phylogenitic tree constructed by fFastmeME [40] (v.2.1.5).

Then, the 414 221 shared single-copy orthologs were utilized to construct the phylogenetic tree. Protein sequences of these orthologs were aligned by MUSCLE [40]. Using the protein alignments, the phylogenetic tree was constructed by the ML (maximum likelihood) TREE algorithm in RAxML software [41, 42] (version 7.2.3). Then mcmctree program of PAML [41] (v.4.5, http://abacus.gene.ucl.ac.uk/software/paml.html) was applied to estimate divergence time among 14 12 species by using the 221 shared single-copy orthologs with main parameters burn-in=100,000, sample-number=100,000, and sample-frequency=2. 4 calibration points were selected from TimeTree website (http://www.timetree.org) as normal priors to restrain the age of the nodes. The phylogenetic tree confirmed the grouping of Angiospermae. The split of *D.oleifera* and *D. lotus* was estimated at 76.99.0 Mya (Fig.454).

Expansion and contraction of gene families

We determined the expansion and contraction of the gene families by comparing the cluster size differences between the ancestor and each species using the CAFÉ program [42]. A random birth and death model were used to study changes of gene families along each lineage of phylogenetic tree. A probabilistic graphical model (PGM) was introduced to calculate the probability of transitions in gene family size from parent to child nodes in the phylogeny. Using conditional likelihoods as the test statistics, we calculated the corresponding p-valuein each lineage and p-value of 0.05 was used to identify families that were significantly expanded and contracted. Compared towith the common

Formatted: Font: Italic

Formatted: Indent: First line: 1 ch

Formatted: Font: Italic

Formatted: Font: Bold

Formatted: Justified, Space Before: 13 pt, After: 13 pt

ancestor of *D.oleifera* and *D. lotus*, 175 gene families (1,896 genes) have expanded in *D.oleifera* (Supplementary Fig. ~~6~~S4) which KEGG pathway enriched in Ubiquitin mediated proteolysis, ABC transporters, Carbon fixation in photosynthetic organisms, etc ~~(Supplementary Fig .S3a)~~. On the contrary, ~~Similarly~~~~In addition,~~ 333 gene families (1,021 genes) were contracted in *D.oleifera*, which KEGG pathway enriched in Plant–pathogen interaction, Phenylpropanoid biosynthesis, Cyanoamino acid metabolism, etc ~~(Supplementary Fig .S3b)~~.

~~Moreover~~In addition, the reference sequences of tannin synthase genes identified in *D.oleifera* were used as a query for blast to identify the homology ~~tannin synthase~~ genes in *D.lotus*, *C.sinensis*, and *V.vinifera*. ~~Source search, taking a~~A sequence with similarity greater than cutoff (50%) and coverage greater than cutoff (50%) were selected as a preliminary candidate gene; then searching for the domain by hmmsearch, only when ~~the~~ it in query was identical with that in subject, the candidate gene was finally retained.~~gene is exactly the same as the subject domain, as a candidate homolog Gene retention.~~ A fisher test (pvalue ≤ 0.05) was performed on the number of individual genes related to tannin synthesis in the genomes of the four species to see if the corresponding gene expanded or contracted. The results showed that compared with other species, *CHS* (Chalcone synthase) genes had different degrees of expansion in *D.oleifera* genome~~,~~ (Supplementary Table S11). CHS is the first key enzyme in the flavonoid-anthocyanin pathway, the expansion of *CHS* gene may be related to the abundant tannin production in the *D.oleifera*. ~~and~~Besides, compared with *V.vinifera*, *LAC* (Laccase) genes showed contraction in *D.oleifera*. It has been reported that LAC may be involved in the polymerization of persimmon tannin monomers, but the specific functions need to be further verified [43,44]. ~~compared with *V.vinifera*, the persimmons had different degrees of expansion with respect to the CHS/STS genes of other species. In addition, the oil persimmon showed contraction compared with grapes.~~

Formatted: Font: Italic
Formatted: Font: Italic
Formatted
Formatted: Indent: First line: 1 ch
Formatted
Formatted
Formatted: Font: (Default) Times New Roman, 12 pt
Formatted: Font: (Default) Times New Roman, 12 pt
Formatted: Font: (Default) Times New Roman, 12 pt
Formatted: Font: (Default) Times New Roman, 12 pt
Formatted: Font: (Default) Times New Roman, 12 pt
Formatted: Font: (Default) Times New Roman, 12 pt
Formatted: Font: (Default) Times New Roman, 12 pt
Formatted: Font: (Default) Times New Roman, 12 pt
Formatted: Font: (Default) Times New Roman, 12 pt
Formatted: Font: (Default) Times New Roman, 12 pt, Italic
Formatted: Font: (Default) Times New Roman, 12 pt
Formatted: Font: (Default) Times New Roman, 12 pt
Formatted: Font: (Default) Times New Roman, 12 pt
Formatted: Font: (Default) Times New Roman, 12 pt
Formatted: Font: (Default) Times New Roman, 12 pt
Formatted: Font: (Default) Times New Roman, 12 pt
Formatted: Font: (Default) Times New Roman, 12 pt
Formatted: Font: (Default) Times New Roman, 12 pt
Formatted: Font: (Default) Times New Roman, 12 pt
Formatted: Font: (Default) Times New Roman, 12 pt
Formatted ...
Formatted ...
Formatted ...
Formatted ...
Formatted: Font: Italic
Formatted: Font: Italic
Formatted: Font: Italic
Formatted: Font: Italic
Formatted ...
Formatted

Positively selected genes in *D.oleifera*

The CDS alignments of 789 single-copy gene families in *D.oleifera*, *D.lotus*，*A.chinensis*, *P.veris*, *R.delavayi* and *S.lycopersicum* were generated using MUSCLE. Gblocks [435] was applied to filter poorly aligned positions and divergent regions of the CDS alignments. With the *D.oleifera* as foreground branch, positive selection sites were detected based on branch-site models43 [446]of PAML 40[41] using the CDS alignments. P-values were computed using the χ^2 statistic and adjusted by FDR method. Finally, 186 genes were positive selected in *D.oleifera* (Supplementary Table S102), which KEGG pathway enriched in Lipoic acid metabolism, One carbon pool by folate, Fatty acid degradation, Thiamine metabolism, Fatty acid elongation, Carotenoid biosynthesis, Fatty acid metabolism, Nucleotide excision repair, Pyrimidine metabolism, Purine metabolism, Starch and sucrose metabolism. GO term enriched in protein N-terminal asparagine amidohydrolase activity, UDP-3-O-[3-hydroxymyristoyl] N-acetylglucosamine deacetylase activity, exosome (RNase complex), transcription initiation from RNA polymerase III promoter, alpha-amylase inhibitor activity, voltage-gated calcium channel activity, orotidine-5-phosphate decarboxylase activity, de novo' pyrimidine nucleobase biosynthetic process, aminoacyl-tRNA hydrolase activity, glutaminase activity, oxaloacetate metabolic process, L-aspartate:2-oxoglutarate aminotransferase activity, aromatase activity, voltage-gated calcium channel complex, 3,5'-cyclic-AMP phosphodiesterase activity, cAMP catabolic process, L-phenylalanine:2-oxoglutarate aminotransferase activity, mitotic chromosome condensation, asparagine metabolic process, condensin complex . Among the 186 PSGs ， *CHI* (Chalcone isomerase) gene was found to be positive selected

Formatted: Not Superscript/ Subscript

Formatted: Not Superscript/ Subscript

Formatted: Not Superscript/ Subscript

Formatted: Not Superscript/ Subscript

Formatted: Not Superscript/ Subscript

Formatted: Font: Italic

(ID:evm.model.original_scaffold_909.101), which was one of the keys~~a key~~ enzymes in the flavonoid-anthocyanin pathway and was~~is~~ -involved in the biosynthesis of persimmon tannins .

Whole-genome duplication and macrosynteny analysis~~.~~

We used BLASTP (E-value < 1e-5) to do homolog or paralog search with the *D.oleifera* and other genomes (*A.chinensis*, *C.canephora*, *C.sinensis*) and MCScanX (s=5, e=1e-5) [~~43~~457] was used to detect syntenic blocks. Then calculate 4dtv (transversion substitutions at fourfold degenerate sites) rates for all syntenic genes to identify putative whole genome duplication or species split events in *D.oleifera*. Besides the ancient whole-genome duplication (WGD) events occurred in all dicots species: γ event (all core eudicots share an ancient WGD, 4dtv = 0.66), a second WGD event occurred in *D.oleifera* and *D.lotus* (4dtv=0.36 ~0.27-0.42) which might contribute to the divergence of Ebenaceae with *A. chinensis* ~~*D.oleifera*~~ and *C. sinensis* (~~Supplementary~~ Fig. ~~S26~~5). Besides, ~~W~~we got 431 syntenic blocks between *D.olerfera* and *D.lotus* ~~(Supplementary Fig. S57)~~. On the whole, except for the translocation of some loci, the sequence of genes between *D.olerfera* and *D.lotus* were relatively conservative (Supplementary Fig. S5). However, compared with *D.olerfera* genome, *D.lotus* genome lacked some regions on each chromosome, which may be lost in the process of anchoring contigs to 15 pseudo-chromosomes using genetic maps. This result further proved the integrity and accuracy of the *D.olerfera* genome assembly.

Conclusions

We generated a high-quality chromosome-level draft genome of *D. oleifera* based on long reads generated by the third-generation PacBio Sequel sequencing platform. The final draft genome was approximately 812.3 Mb, slightly smaller than the 868.4 Mb estimated by k-mer analysis. The Hi-C data were combined with the assembled draft genome to generate chromosome-length scaffolds.

Formatted: Font: Not Italic
Formatted: Font: Italic
Formatted: Font: Italic
Formatted: Font: Italic
Formatted: Font: Italic
Formatted: Font: Italic
Formatted: Justified

As a result, 15 scaffolds corresponding to the 15 chromosomes were assembled; these comprised 721.5 Mb and 332 scaffolds, with an N50 of 33.5 Mb, and accounted for 88.81% of the genome. ~~In addition,~~ 30,539 protein-coding genes ~~with an average transcript size of 1,080.9 bp~~ were predicted, and 92.2% (28,146 genes) of all protein-coding genes were annotated~~., 109 of which were related to the synthesis of tannin, 3 were related to sex determination~~. Also, repeat sequences accounted for 54.8% of the genome, and 564 miRNAs, 507 tRNAs, 2,207 rRNAs, and 803 snRNAs were identified. In addition, 171 candidate genes involved in the synthesis of tannin and deastringency were identified, of which *CHS* genes had different degrees of expansion in *D.oleifera* genome compared with- *D.lotus*, *C.sinensis*, and *V.vinifera*. Moreover, 186 positively selected genes were identified, including *CHI* gene, which was one of the key~~-s~~ enzymes in the flavonoid-anthocyanin pathway. The divergence time ~~of~~ between *D. oleifera* and *D.lotus* was estimated at ~~76.9~~ 9.0 Mya, and two WGD events occurred in *D.oleifera* genome. ~~To our knowledge, our chromosome-level genome assembly of *D. oleifera* is the first reference genome of a member of the family Ebenaceae.~~ The high-quality chromosomal genome assembly of ~~assembled genome~~*D.oleifera* will facilitate both research on the major economic traits ~~the mechanisms of sex determination~~ in the genus *Diospyros* and assembly of the hexaploid persimmon genome.

Availability of supporting data

The data set supporting the results of this article is available in the NCBI Sequence Read Archive [Accessions: PRJNA532832].

Additional files

Fig. S1: k-mer distribution of the *D.olefera* genome.

~~-~~Fig. S2: Tannins sythnase genes and deastringency process in *Diospyros*.

Fig. S3: Venn diagram of gene family cluster of five Ericales species.

Formatted: Font: Italic

Formatted: Font: Italic

Formatted: Underline color: Custom Color(RGB(34,34,34)), Expanded by 0.15 pt

Formatted: Font: Italic

Fig. S4: Gene family expansion and contraction analysis of 12 species.

Fig. S5: Macrosynteny analysis between *D.olefera* genome and *D.lotus* genome. a: gene density; b: LINE transposon density; c: LTR transposon density; d: DNA transposon density; e: GC density (density is calculated in units of 500 kb).

~~Fig. S2: Whole-genome duplication analysis of *D. oleifera* genome. Dol (*D.oleifera*), Ach (*A.chinensis*), Cca (*C.canephora*), Csi(*C.sinensis*).~~

Table S1: The sequencing data size by various sequencing platform.

Table S2: Estimation of *D.oleifera* genome size by K-mer analysis.

Table S3: Chromosomes length using HiC reads.

Table S4: Mapping rate of reads to *D.oleifera* genome assembly.

Table S5: CEGMA assessment of *D.oleifera* genome.

Table S6: BUSCO notation assessment of *D.oleifera* genome.

Table S7: Gene annotation of *D.oleifera* genome via three methods.

Table S8: *D.oleifera* genome gene annotation statistics used different databases.

Table ~~S8~~S9: *D.oleifera* genome ncRNA annotation statistics used different databases.

Table ~~S9~~S10: Genes involved in tannins sythnase genes and deastringency process in *D. oleifera*.

Table S11: Expansion and contraction of tannin synthase genes in *D.oleifera*.

Table S1~~01~~2： Positively selected genes in *D.oleifera*.

Abbreviations

BWA: Burrows-Wheeler Aligner; BLAST: Basic Local Alignment Search Tool; BUSCO: Benchmarking Universal Single-Copy Orthologs; NCBI: National Center for Biotechnology Information; PacBio: Pacific Biosciences; RNA-seq: RNA-sequencing; SMRT sequencing: single molecule real time sequencing; TE: transposable element; WGD: whole-genome duplication.

Competing interests

The authors declare that they have no competing interests.

Funding

This work was supported by the National Key R &D Program of China (2018YFD1000606), and the Fundamental Research Funds for the Central Non-profit Research Institution of CAF (CAFYBB2017ZA005 and CAFYBB2017ZA004-3)

Author contributions

J.M.F. and F.D.L. conceived the project. W.J.H., H.W.L. and S.F.D. collected the samples, Y.J.S., P.S. and Y.N.M. conducted genome assembly and data analysis, X.Z. provided intellectual insights, Y.J.S., P.S. and H.H.C. wrote the paper. All authors read and wrote part of the manuscript.

The English in this document has been checked by at least two professional editors, both native speakers of English. For a certificate, please see: http://www.textcheck.com/certificate/Q48QsC

Formatted: HTML Preformatted, Justified, Tab stops: Not at 0.64″ + 1.27″ + 1.91″ + 2.54″ + 3.18″ + 3.82″ + 4.45″ + 5.09″ + 5.73″ + 6.36″ + 7″ + 7.63″ + 8.27″ + 8.91″ + 9.54″ + 10.18″

Formatted: Font: Font color: Black, (Asian) Chinese (PRC), Border: : (No border)

Formatted: English (United Kingdom)

References

1. Fu JM, Liu HM, Hu JJ, et al. Five Complete Chloroplast Genome Sequences from *Diospyros*: Genome Organization and Comparative Analysis. PLOS ONE, 2016, 11(7):e0159566-.
2. Akagi T, Henry IM, Tao R, et al. A Y-chromosome-encoded small RNA acts as a sex determinant in persimmons. Science, 2014, 346(6209):646-650.
3. Lieberman-Aiden E, et al. Comprehensive mapping of long-range interactions reveals folding principles of the human genome. Science, 2009, 326: 289–293.

4. Liu, B. et al. Estimation of genomic characteristics by analyzing k-mer frequency in de novo genome projects. arXiv: Genomics, 2013.

5. Chin CS, Peluso P, Sedlazeck FJ, et al. Phased diploid genome assembly with single-molecule real-time sequencing. Nature Methods, 2016.

6. Chin CS, Alexander DH, Marks P, et al. Nonhybrid, finished microbial genome assemblies from long-read SMRT sequencing data. Nature Methods, 2013, 10(6):563.

7. Walker BJ, Abeel T, Shea T, et al. Pilon: An Integrated Tool for Comprehensive Microbial Variant Detection and Genome Assembly Improvement. PLOS ONE, 2014, 9.

8. Adey A, Kitzman JO, Burton JN, et al. In vitro, long-range sequence information for de novo genome assembly via transposase contiguity. Genome Research, 2014, 24(12):2041-2049.

9. Li H, and Durbin R. Fast and accurate short read alignment with Burrows-Wheeler transform. Bioinformatics, 2009.

10. Akagi T, Shirasawa K, Nagasaki H, et al. The persimmon genome reveals clues to the evolution of a lineage-specific sex determination system in plants. bioRxiv 628537; doi: https://doi.org/10.1101/628537

~~9.~~

~~10.~~11. Parra G, Bradnam K, Korf I. CEGMA: a pipeline to accurately annotate core genes in eukaryotic genomes. Bioinformatics, 2007, 23(9):1061-1067.

~~11.~~12. Simao FA, Waterhouse RM, Ioannidis P, et al. BUSCO: assessing genome assembly and annotation completeness with single-copy orthologs. Bioinformatics, 2015:btv351.

~~12.~~13. Smit AF, Hubley R, Green P. RepeatMasker at <http://www.repeatmasker.org/RepeatModeler.html>.

~~13.~~14. Chen, N. Using RepeatMasker to identify repetitive elements in genomic sequences. *Curr Protoc* Bioinformatics, 2004, Chapter 4, Unit 4.

~~14.~~15. Price AL, Jones NC, Pevzner PA . De novo identification of repeat families in large

Formatted: Pattern: Clear

Formatted: Font: (Default) Times New Roman

Formatted: Indent: Left: 0", Hanging: 0.25", Line spacing: Double

Formatted: Font: (Default) Times New Roman

Formatted: Font: (Default) Times New Roman

Formatted: Font: (Default) Times New Roman

Formatted: Font: (Default) Times New Roman

Formatted: Font: (Default) Times New Roman

Formatted: Font: (Default) Times New Roman

Formatted: Font: (Default) Times New Roman

Formatted: Font: (Default) Times New Roman

Formatted: Font: (Default) Times New Roman

Formatted: Font: (Default) Times New Roman

Formatted: Font: (Default) Times New Roman, Font color: Black

genomes. Bioinformatics, 2005, 21(Suppl 1):i351-i358.

~~15.~~16. Xu Z, Wang H. LTR_FINDER: an efficient tool for the prediction of full-length LTR retrotransposons. Nucleic Acids Research, 2007, 35(Web Server):W265-W268.

~~16.~~17. Benson G. Tandem repeats finder: a program to analyze DNA sequences. Nucleic Acids Res. 1999, 27, 573-580.

~~17.~~18. Stanke M, Waack S. Gene prediction with a hidden Markov model and a new intron submodel. Bioinformatics, 2003, 19, ii215–25.

~~18.~~19. Stanke M, Sch?Ffmann O, Morgenstern B, et al. Gene prediction in eukaryotes with a generalized hidden Markov model that uses hints from external sources. Bmc Bioinformatics, 2006, 7(1):62-0.

~~19.~~20. Salamov, A. A. Ab initio gene finding in Drosophila genomic DNA. Genome Res, 2000, 10, 516–522.

~~20.~~21. Parra G, Blanco E, Guigó R. GeneID in Drosophila. Genome Research, 2000, 10(4):511.

~~21.~~22. Majoros WH, Pertea M , Salzberg SL . TigrScan and GlimmerHMM: two open source ab initio eukaryotic gene-finders. Bioinformatics, 2004, 20(16):2878-2879.

~~22.~~23. Korf I. Gene finding in novel genomes. BMC Bioinformatics, 2004, 5, 59.

~~23.~~24. Altschul SF, et al. Gapped BLAST and PSI-BLAST: a new generation of protein database search programs. Nucleic Acids Res, 1997, 25, 3389-402.

~~24.~~25. Birney E, Clamp M, Durbin R. Genewise and genomewise. Genome Res, 2004, 14, 988–995.

~~25.~~26. Trapnell C, Pachter L, Salzberg SL. TopHat: Discovering splice junctions with RNA-Seq. Bioinformatics, 2009, 25, 1105-11.

~~26.~~27. Trapnell C, Williams BA, Pertea G, et al. Transcript assembly and quantification by RNA-Seq reveals unannotated transcripts and isoform switching during cell differentiation. Nature Biotechnology, 2010, 28(5):511-515.

~~27.~~28. Grabherr MG, Haas BJ, Yassour M, et al. Full-length transcriptome assembly from RNA-Seq data without a reference genome. Nature Biotechnology, 2011, 29(7):644-652.

~~28.~~29. Haas BJ, Delcher AL, Mount SM, et al. Improving the Arabidopsis genome annotation using maximal transcript alignment assemblies. Nucleic Acids Research, 2003, 31(19):5654-5666.

~~29.~~30. Haas BJ, Salzberg SL, Zhu W, et al. Automated eukaryotic gene structure annotation using EVidenceModeler and the Program to Assemble Spliced Alignments. Genome biology, 2008, 9(1).

~~30.~~31. Bairoch A, Apweiler R. The SWISS-PROT protein sequence database and its supplement TrEMBL in 2000. Nucleic Acids Res, 2000, 28, 45-48.

~~31.~~32. Mulder N, Apweiler R. InterPro and InterProScan: tools for protein sequence classification and comparison. Methods Mol Biol, 2007, 396, 59-70.

~~32.~~33. Ashburner M, Ball CA, Blake JA, et al. Gene ontology: tool for the unification of biology. The Gene Ontology Consortium. Nat Genet, 2000, 25, 25-29.

~~33.~~34. Kanehisa M, Goto S. KEGG: kyoto encyclopedia of genes and genomes. Nucleic Acids Res, 2000, 28, 27-30.

~~34.~~35. Lowe TM, Eddy SR. tRNAscan-SE: A Program for Improved Detection of Transfer RNA Genes in Genomic Sequence. Nucleic Acids Res, 1997, 25, 955-964.

~~35.~~36. Nawrocki EP, Kolbe DL, Eddy SR. Infernal 1.0: inference of RNA alignments. Bioinformatics, 2009, 25(10):1335.

~~36.~~37. Nawrocki EP, Eddy SR. Infernal 1.1: 100-fold faster RNA homology searches. Bioinformatics, 2013, 29(22):2933-2935.

~~37. Akagi T, Henry IM , Ohtani H, et al. A Y-encoded suppressor of feminization arose via lineage-specific duplication of a cytokinin response regulator in kiwifruit. The Plant Cell, 2018:tpc.00787.2017.~~

38. ~~Li, L. OrthoMCL: Identification of Ortholog Groups for Eukaryotic Genomes. Genome~~

~~Research, 2003, 13(9):2178-2189.~~Emms D~~-~~M~~-~~, Kelly S~~-~~. OrthoFinder: solving fundamental biases in whole genome comparisons dramatically improves orthogroup inference accuracy~~[J]~~. Genome Biology, 2015, 16(1):157.

39. Yu XJ, Zheng HK, Wang J, et al. Detecting lineage-specific adaptive evolution of brain-expressed genes in human using rhesus macaque as outgroup. Genomics, 2006, 88(6):0-751.

40. ~~Edgar RC. MUSCLE: multiple sequence alignment with high accuracy and high throughput. Nucleic Acids Res, 2004, 32: 1792-1797.~~Lefort V~~-~~, Desper R~~-~~, Gascuel O~~-~~. FastME 2.0: A Comprehensive, Accurate, and Fast Distance-Based Phylogeny Inference Program: Table 1.~~[J]~~. Molecular Biology and Evolution, 2015:msv150.

41. Yang Z. ~~(2007)~~ PAML 4: Phylogenetic Analysis by Maximum Likelihood. Mol. Biol. Evol. 2007, 24: 1586-1591.

42. De Bie~~,~~ T~~.~~, Cristianini~~,~~ N~~.~~, Demuth~~,~~ J~~.~~-P~~.~~, & Hahn~~,~~ M~~.~~-W. CAFE: a computational tool for the study of gene family evolution. Bioinformatics,2006, 22(10), 1269-1271~~(2006)~~.

43. Zhao J， Pang Y， Dixon R-A. ~~2010.~~ The mysteries of proanthocyanidin transport and polymerization. Plant Physiology，2010,153 (2)：437－443.

44. Hu Q-N，Luo C，Zhang Q-L，Luo Z-R. ~~2013.~~ Isolation and characterization of a laccase gene potentially involved in proanthocyanidin polymerization in oriental persimmon（*Diospyros kaki* Thunb.）fruit. Molecular Biology Reports，2013, 40 (4)：2809－2820.

45. Castresana~~,~~ J. Selection of conserved blocks from multiple alignments for their use in phylogenetic analysis. Molecular Biology and Evolution,2000, 17: 540-552. ~~(2000).~~

46. Zhang~~,~~ J~~.~~, Nielsen~~,~~ R~~. &~~, Yang~~,~~ Z. Evaluation of an Improved Branch-Site Likelihood Method for Detecting Positive Selection at the Molecular Level. Mol Biol Evol.,2005, 22, 2472-2479 ~~(2005)~~.

47. Wang Y, Tang H, Debarry JD, et al. MCScanX: a toolkit for detection and evolutionary analysis of gene synteny and collinearity. Nucleic Acids Research, 2012, 40(7):e49-e49.

Formatted: Indent: Left: 0", Hanging: 0.26", Line spacing: Double

Formatted: Font: Italic

Formatted: Indent: Left: 0", Hanging: 0.26"

-
40.

41. Stamatakis A. RAxML-VI-HPC: maximum likelihood-based phylogenetic analyses with thousands of taxa and mixed models. Bioinformatics, 2006, 22: 2688-2690.

42. Stamatakis A, Hoover P, Rougemont J. A Rapid Bootstrap Algorithm for the RAxML Web Servers. Systematic Biology, 2017, 57(5):758-771.

43.48. Wang Y, Tang H, Debarry JD, et al. MCScanX: a toolkit for detection and evolutionary analysis of gene synteny and collinearity. Nucleic Acids Research, 2012, 40(7):e49-e49.

Formatted: Indent: Left: 0.26", No bullets or numbering

Formatted: No bullets or numbering

Formatted: Indent: First line: 0"

 Click here to access/download;Table;Tables.xlsx

Table 1 Summary of the *D.oleifera* genome assembly using Pacbio long-read, Illumina reads and 10X genomics data

Program	SampleID	Length		Number	
		Contig(bp)	Scaffold(bp)	Contig	Scaffold
**Falcon**	Total	806,744,914	-	2,986	-
	N50	2,916,360	-	72	-
**Quiver**	Total	812369941	-	2,986	-
	N50	2,938,972	-	72	-
**Pilon**	Total	811,094,501	-	2986	-
	N50	2,937,127	-	72	-
**10X**	Total	811,094,501	812,323,628	2,986	2,812
	Max	14,814,786	17,432,797	-	-
	Number>=2000	-	-	2,803	2,629
	N50	2,937,127	3,359,874	71	62
	N60	2,314,962	2,662,781	103	89
	N70	1,622,862	1,911,995	144	125
	N80	790,034	1,007,083	214	182
	N90	196,816	257,477	421	333

Table 2 Classification of repetitive elements in *D.oleifera* genome

total repeative elements

Program	Repeat Size(bp)	% of genome
Trf	79,886,467	9.83
Repeatmasker	408,623,327	50.3
Proteinmask	22,154,795	2.73
Total	445,187,963	54.8

Transposons elements

Type		Transposons Elementss Length(bp)	% in Genome
DNA		33,844,732	4.17
LINE		13,187,364	1.62
SINE		74,819	0.01
LTR	total	379,582,766	46.73
	Gypsy	216,328,284	26.63
	Copia	116,970,626	14.40
	other	46,283,856	5.70
Unknown		10,513,280	1.29
Total		430,778,122	53.03

Fig. 1

Click here to access/download;Figure;Fig. 1.png

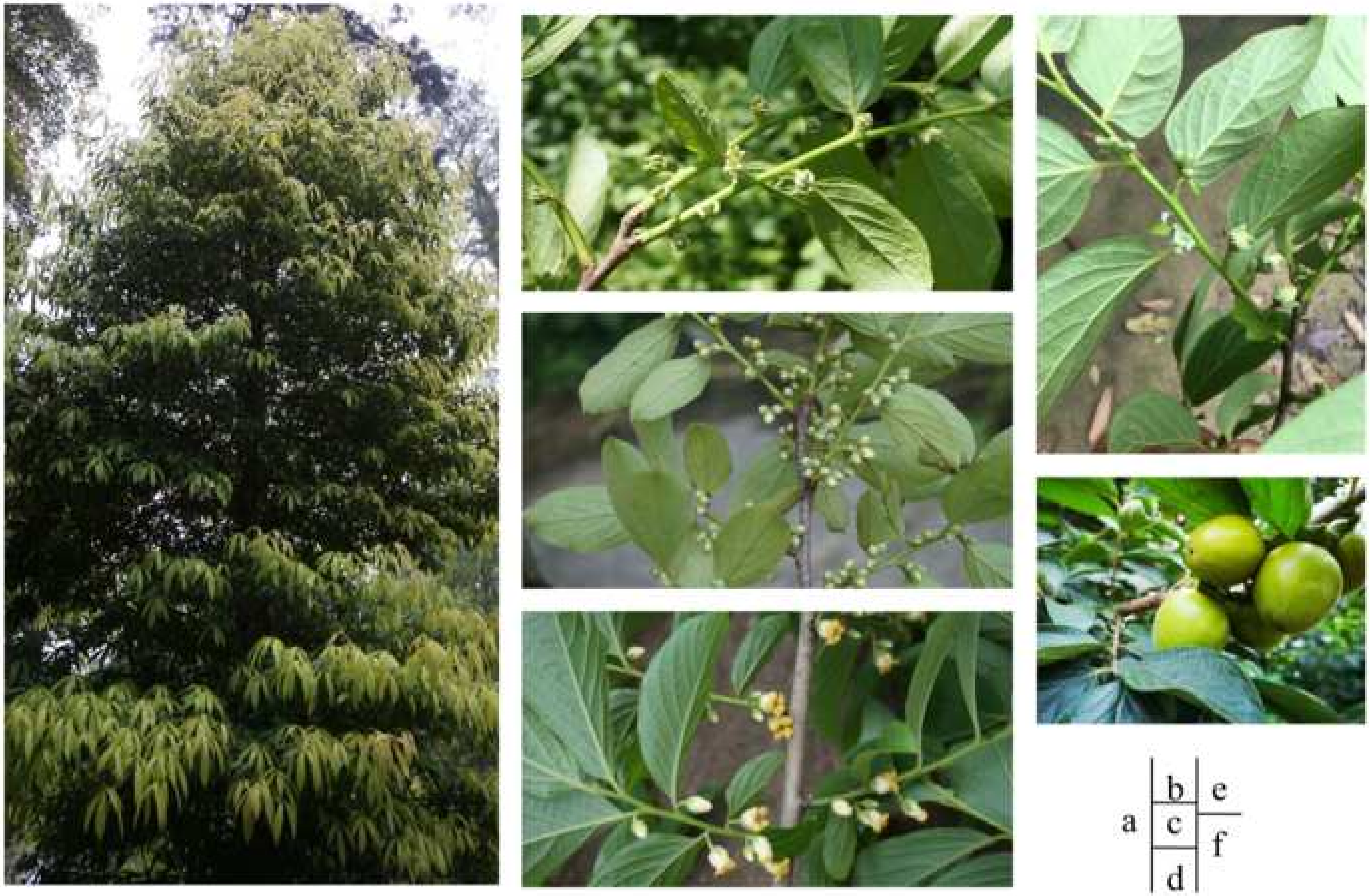

Fig. 2

Click here to access/download;Figure;Fig. 2.png

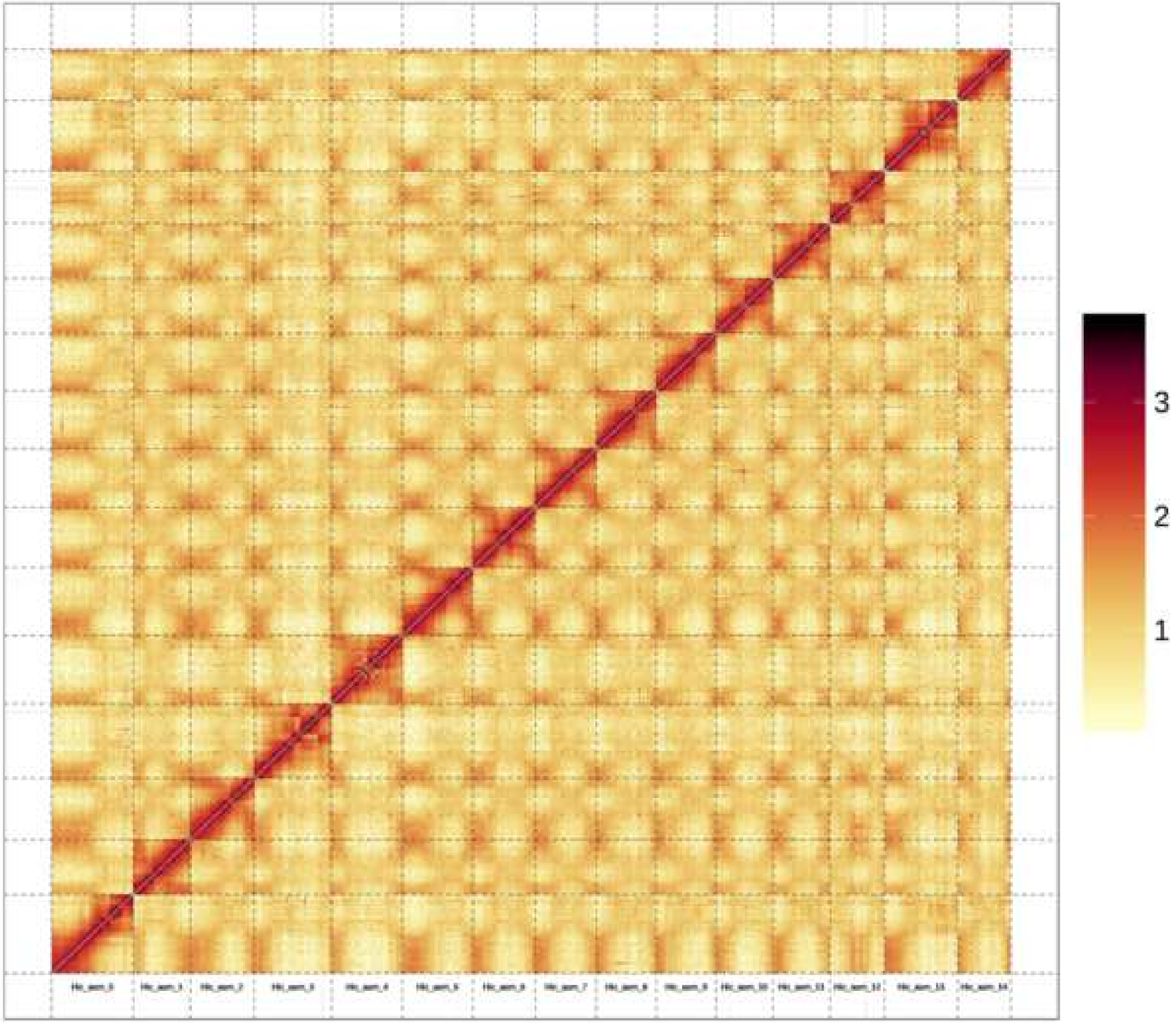

Fig. 3 Click here to access/download;Figure;Fig. 3.png

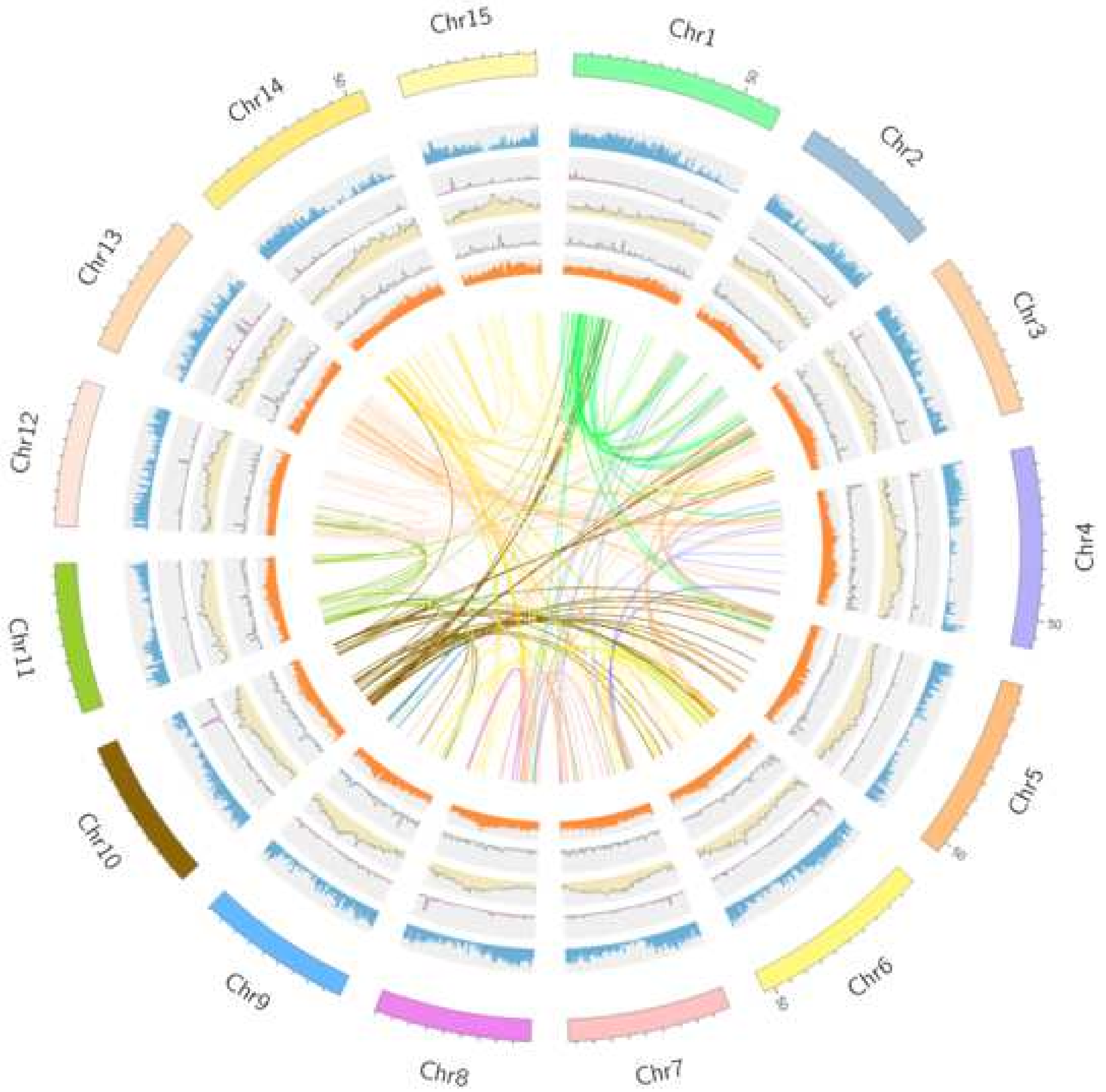

Fig. 4

Click here to access/download;Figure;Fig. 4.png

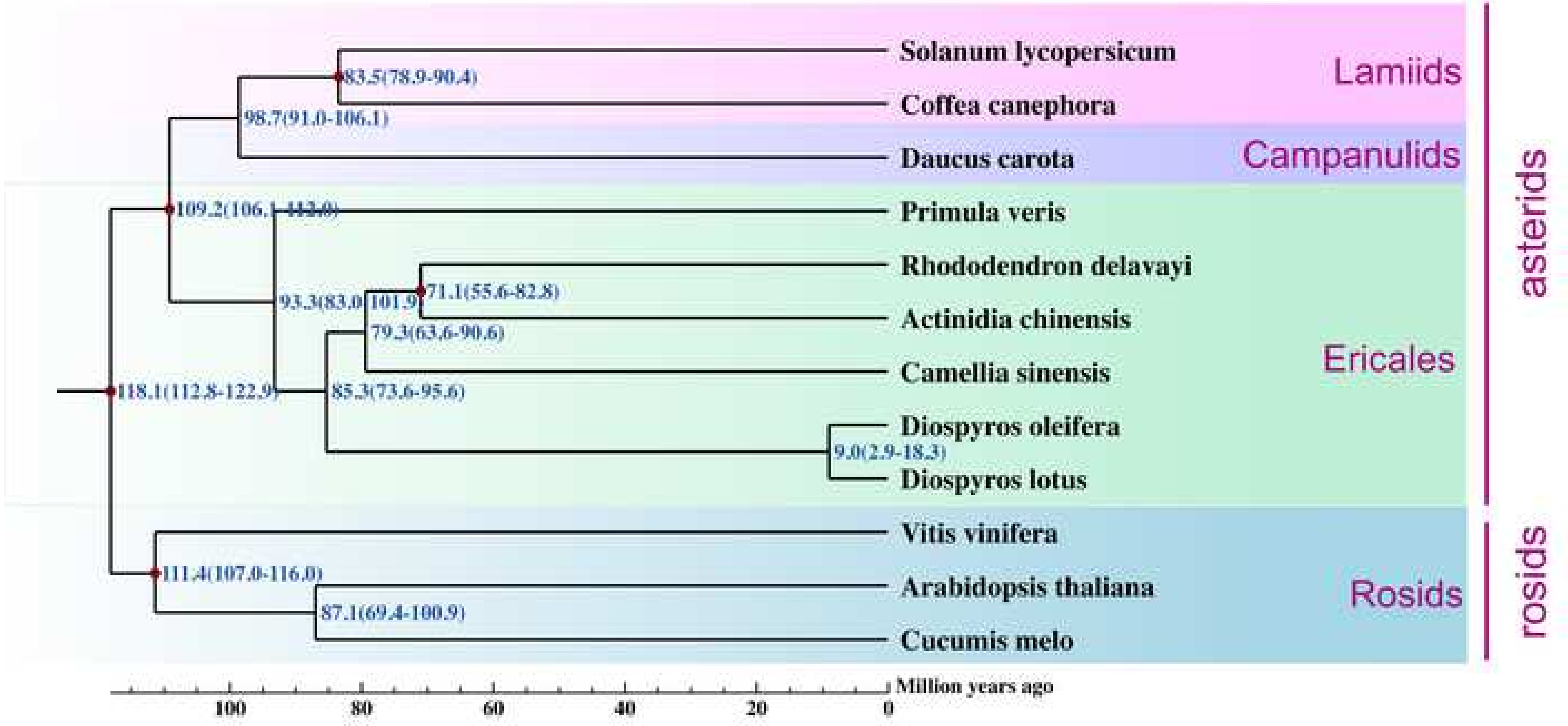

Fig. 5

Click here to access/download;Figure;Fig. 5.png

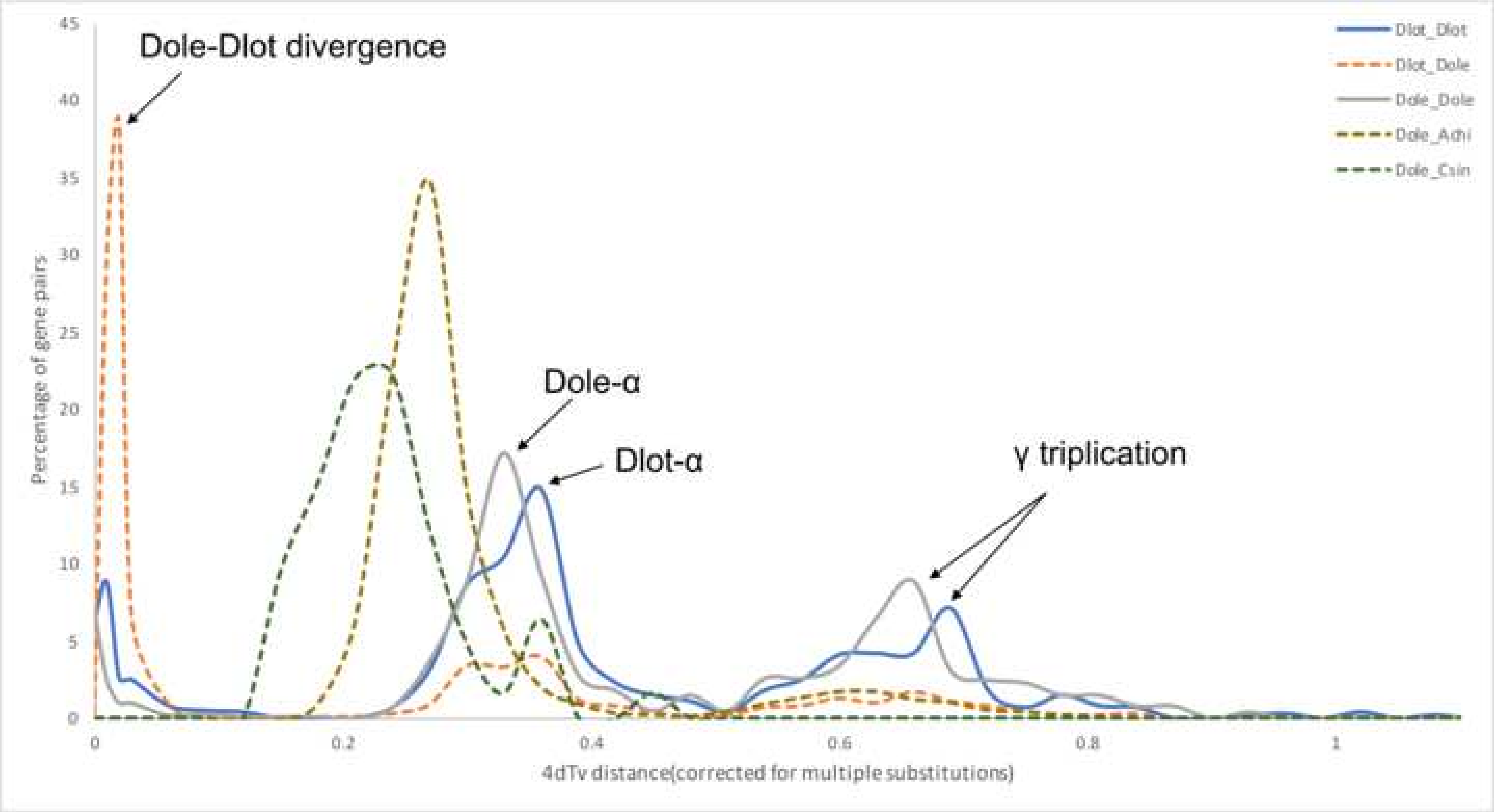

Fig. S1

Click here to access/download
Supplementary Material
Fig. S1.png

Fig. S2

Click here to access/download
Supplementary Material
Fig. S2.png

Fig. S3

Click here to access/download
Supplementary Material
Fig. S3.png

Fig. S4

Click here to access/download
Supplementary Material
Fig. S4.png

Fig. S5

Click here to access/download
Supplementary Material
Fig. S5.png

Click here to access/download
Supplementary Material
Supplementary Tables.xlsx

Personal Cover
Click here to access/download;Personal Cover;Response to Reviewers.docx

Dear Editor,

Thank you very much for offering us the opportunity to resubmit a revised version of our manuscript. Hereby, we submit the revised manuscript entitled "**A high-quality chromosomal genome assembly of *Diospyros oleifera*" (GIGA-D-19-00174)** to **GigaScience**. We appreciate the valuable comments and suggestions from you and the reviewers, which help us to improve and clarify the manuscript. We have discussed the comments carefully and tried our best to improve the manuscript according to the reviewers' good comments.

The main modifications are as follows:

1. We confirmed the NCBI data link, you and the reviewers can find the data under this link: https://dataview.ncbi.nlm.nih.gov/object/PRJNA532832?reviewer=gbss3pp9p06h5hosks3vrepirg

2. We supplemented the content of comparative genomic analysis, including gene family cluster, expansion and contraction of gene families, positively selection analysis, and macrosynteny analysis. Some interesting results were found: Firstly, compared with *D.lotus*, *C.sinensis*, and *V.vinifera*, *CHS* (Chalcone synthase) genes (the first key enzyme in the flavonoid-anthocyanin pathway) had different degrees of expansion in *D.oleifera* genome, which may be contribute to the abundant tannin production in the *D.oleifera*. Besides, *CHI* (Chalcone isomerase) gene (another key enzyme in the flavonoid-anthocyanin pathway) was found to be positive selected. These results will provide important data support for the molecular mechanism analysis of the major economic traits in *Diospyros*. Furthermore, the divergence time of between *D. oleifera* and *D.lotus* was estimated at 9.0 Mya, and two WGD events occurred in *D.oleifera* genome. These results will be helpful for the further analysis of the evolution of *Diospyros* species, and the origin of hexaploid persimmon.

Detailed responses to your and the reviewers' comments are provided in the next sections. We hope you and the reviewers will be satisfied with our responses to the comments and the revisions for the original manuscript. Please feel free to contact us with any questions and we are looking forward to your response.

Thanks and Best wishes!

Yours sincerely,
Yujing Suo and Jianmin Fu

Response to Reviewer:

Reviewer #1: Suo et al report a chromosome scale assembly of D. oleifera, a diploid relative of hexaploid persimmon. They used a combination of Illumina, 10x, PacBio, and HiC to generate the chromosome scale assembly D. oleifera. The inclusion of high coverage Illumina data and scaffolding with 10x likely ensured that most of the residual indels from the PacBio only assembly were corrected. The HiC contact map in Figure 2 has no obvious inversions or misplacements, suggesting the genome is well assembled. This resource will be useful for the comparative genomics and persimmon research communities. I have a few minor concerns that should be addressed before this manuscript is published.

Q1. The estimated heterozygosity of D. oleifera is quite high (1.1%) and this would have likely resulted in assembly issues related to haplotype specific contigs. How many primary and alternate contigs were assembled by FALCON?

A: There were 2,986 contigs initially assembled by FALCON (Table 1).

Q2.Akagi et al. (https://www.biorxiv.org/content/early/2019/05/05/628537.full.pdf) report a chromosome scale assembly of diploid persimmon (D. lotus). The authors could cite this preprint in their manuscript and if the genome is publicly available, survey macrosynteny.

A: Thanks for your suggestion. We have added the macrosynteny analysis (Revised manuscript，Page12，Line 291-297). The chromosome-based macrosynteny analysis revealed a striking correspondence between *D.oleifera* and *D.lotus*, for that there were totally 432 syntenic blocks showed in supplementary Fig. S5.

Q3. The identification of homologs of sex determination genes from kiwi and D. lotus is not informative, as the kiwi sex determination system is likely completely different from D. oleifera and sex chromosomes may have an independent origin in D. lotus and D. oleifera. It is fine to leave this in the paper, but the statement that candidate sex determination genes were identified should be removed from the abstract

A: Thanks for your suggestion. We have removed the content about sex determination, and focused on the analysis of genes related to tannin synthesis.

Q 4. The identification of a WGD event in D. oleifera is interesting, and figure S2 could probably be moved to the main text. Based on this figure, it looks like there could have been two WGD events in D. oleifera.

A: Thanks for your suggestion. We have moved the Fig. S2 to the main text as the new Fig. 5. Besides the ancient γ event (all core eudicots share an ancient WGD, 4dtv = 0.66), a second WGD event occurred in *D.oleifera* and *D.lotus* (4dtv=0.36 ~0.27-0.42) which might contribute to the divergence of Ebenaceae with *A. chinensis* and *C. sinensis*. (Revised manuscript，Page12，Line 289-292)

Minor

Page 6, line 152. homologous should not be use here

Versions are provided or most but not all bioinformatics software. Where appropriate, versions should be added.

A: ‘homologous’ in Page 6, line 152 was deleted and versions of bioinformatics software had been provided in the article. (Revised manuscript，Page7，Line 165-166)

Reviewer #2: This manuscript describes the assembly of the first chromosome-level genome sequence of an Ebenaceae, Diospyros oleifera. The newly generated genome sequence was analysed for TE and gene content as well as for tannin synthase and sex determination genes. A phologenetic tree was constructed for divergence time estimation.

Data preparation as well as the construction of the pseudomolecules follows established and proven protocols and the results look good to me. Same is true for the gene model prediction and TE detection.

My main issue with this study is that it is almost exclusively a description of a newly established genomic resources, with very little to no new biological insights included in this manuscript. There is a little bit on tannin synthase and sex determination but this is all based on existing knowledge and little more than a homolog search. I appreciate the generation of these novel and helpful resources but these data could/should have been used to gain more biological insights.

A: For the question you mentioned, we supplemented the content of comparative genomic analysis in the revised manuscript, including gene family cluster, expansion and contraction of gene families, positively selection analysis, and macrosynteny analysis. Some interesting results were found: Firstly，compared with *D.lotus*, *C.sinensis*, and *V.vinifera*, *CHS* (Chalcone synthase) genes (the first key enzyme in the flavonoid-anthocyanin pathway) had different degrees of expansion in *D.oleifera* genome, which may be contribute to the abundant tannin production in the *D.oleifera*. Besides, *CHI* (Chalcone isomerase) gene (another key enzyme in the flavonoid-anthocyanin pathway) was found to be positive selected. These results will contribute to the molecular mechanism analysis of the major economic traits in *Diospyros*. Furthermore，the divergence time of between *D. oleifera* and *D.lotus* was estimated at 9.0 Mya, and two WGD events occurred in *D.oleifera* genome. These results will be helpful for the further analysis of the evolution of Diospyros species, and the origin of hexaploid persimmon.

In brief, this study provides a high-quality chromosomal level assembly of *D.oleifera* genome, which will provide important data support for the assembly of subsequent hexaploid persimmon genomes and the molecular mechanism analysis of the major economic traits in *Diospyros*.

More specific issues:

a.) Transcriptome data: I could not find a proper description of the transcriptome data that was obviously generated with this study and used for gene prediction. This could e.g. also be used to establish transcription levels for gene or gene families of interest.

A: The description of the transcriptome data was displayed in the 'Genomic RNA extraction, library construction and sequencing' part of the article. (Revised manuscript, Page7, Line 156-160)

b.) Functional annotation: I would recommend to use more specialized tools such as AHRD or BLAST2GO instead of simple best Blast hit for the human readable descriptions.

A: Thanks for your suggestion. We had reannotated the gene set by BLAST2GO, as a result, 19,900 genes were annotated. After combining with the earlier annotation by blastp with InterPro database, there were totally 20,826 genes that had GO annotation, account for 68.20% of the gene models. The results were showed in the article. (Revised manuscript, Page8, Line 186-191)

c.) Phylogeny: I would recommend to use OrthoFinder instead of the older OrthoMCL version for determining the orthologous groups. Also, I'm not sure about the bias possibly introduced by just picking the single copy orthologs for the construction of the phylogenetic tree.

A: Thanks for your suggestion. We had reanalysis the gene families with OrthoFinder and got totally 19,722 clusters which were used for the phylogeny construction. The results were displayed in the article. (Revised manuscript, Page9, Line 224-230)

d.) I could not access any data under the NCBI accession number given.

A: We confirmed the NCBI data link, you and the reviewers can find the data under this link: https://dataview.ncbi.nlm.nih.gov/object/PRJNA532832?reviewer=gbss3pp9p06h5hosks3vrepirg

e.) Language and grammar needs improvement.

A: The English in this document has been checked by at least two professional editors, both native speakers of English. For a certificate, please see: http://www.textcheck.com/certificate/Q48QsC
