# Supplementary material for: A high-quality chromosomal genome assembly of Diospyros oleifera Cheng
Source: Gigascience. 2020 Jan 16;9(1):giz164. doi: 10.1093/gigascience/giz164 (PMC6964648; doi:10.1093/gigascience/giz164)
Supplement: giz164_GIGA-D-19-00174_Revision_2 [file giz164_giga-d-19-00174_revision_2.pdf]

GigaScience

A high-quality chromosomal genome assembly of Diospyros oleifera

--Manuscript Draft--

**Manuscript Number:**	GIGA-D-19-00174R2
**Full Title:**	A high-quality chromosomal genome assembly of Diospyros oleifera
**Article Type:**	Data Note
**Funding Information:**	National Key R &D Program of China (2018YFD1000606) — Dr. Jianmin Fu<br>Fundamental Research Funds for the Central Non-profit Research Institution of CAF (CAFYBB2017ZA005) — Dr. Peng Sun<br>Fundamental Research Funds for the Central Non-profit Research Institution of CAF (CAFYBB2017ZA004-3) — Dr. Fangdong Li
**Abstract:**	Background: Diospyros oleifera, a member of the genus Diospyros of the family Ebenaceae, is an economically important tree. Phylogenetic analyses have indicated that D. oleifera is closely related to D. kaki, and could be used as a model plant for studies of D. kaki. Therefore, development of genomic resources of D. oleifera will facilitate auxiliary assembly of the hexaploid persimmon genome and provide insight into the molecular mechanisms of major economic traits. Findings: The D. oleifera genome was assembled into 443.6 Gb of raw reads using the Pacific Bioscience Sequel and Illumina HiSeq X Ten platforms. The final draft genome was approximately 812.3 Mb and had a high level of continuity with the 3.36 Mb N50. Using the Hi-C data and the draft genome assembly, 15 scaffolds corresponding to the 15 chromosomes were assembled to a final size of 721.5 Mb using 332 scaffolds, accounting for 88.81% of the genome. The identified repeat sequences accounted for 54.8% of the genome. By de novo sequencing and analysis of homology with other plant species, 30,530 protein-coding genes with an average transcript size of 7,105.40 bp were annotated; of these, 28,580 protein-coding genes (93.61%) had conserved functional motifs or terms. In addition, 171 candidate genes involved in the tannin synthesis and deastringency in persimmon were identified; of these chalcone synthase (CHS) genes expanded in the D. oleifera genome compared with D. lotus, C. sinensis, and V. vinifera. Moreover, 186 positively selected genes were identified, including chalcone isomerase (CHI) gene, which is a key enzyme in the flavonoid-anthocyanin pathway. Phylogenetic tree analysis indicated that the split of D. oleifera and D. lotus likely occurred 9.0 million years ago. In addition to the ancient γ event, a second whole-genome duplication event occurred in D. oleifera and D. lotus (4dtv = 0.36 ~ 0.27-0.42). Conclusions: We generated a high-quality chromosome-level draft genome for D. oleifera, which will facilitate assembly of the hexaploid persimmon genome and further studies of major economic traits in the genus Diospyros.
**Corresponding Author:**	Yujing Suo<br><br>CHINA
**Corresponding Author Secondary Information:**	
**Corresponding Author's Institution:**	
**Corresponding Author's Secondary Institution:**	
**First Author:**	Yujing Suo
**First Author Secondary Information:**	
**Order of Authors:**	Yujing Suo<br>Peng Sun

	Huihui Cheng
	Weijuan Han
	Songfeng Diao
	Huawei Li
	Yini Mai
	Xing Zhao
	Fangdong Li
	Jianmin Fu
**Order of Authors Secondary Information:**	
**Response to Reviewers:**	Dear Editor,<br><br>Thank you very much for offering us the opportunity to resubmit a revised version of our manuscript. Hereby, we submit the revised manuscript entitled “A high-quality chromosomal genome assembly of Diospyrosoleifera” (GIGA-D-19-00174) to GigaScience. We appreciate the valuable comments and suggestions from you and the reviewers, which help us to improve and clarify the manuscript. We have discussed the comments carefully and tried our best to improve the manuscript accordingly.<br><br>This study focuses on the genome sequencing, assembly and annotation of D. oleifera, moreover comparative genomic analyses with other species were also included. The purpose of this study is to provide accurate genomic information for the further studies on molecular mechanisms underlying the formation and regulation of important economic traits of Diospyros spp. Based on this present study, some biological issues such as sex differentiation of flowers and natural deastringence of fruits in persimmon are being studied, the results of which will be reported in the future. Additionally, we heard that a similar study has been conducted by another research group, and they have submitted their manuscript to another journal. Due to this information, we deeply appreciate that if the review progress of our manuscript could be accelerated.<br><br>Detailed responses to your and the reviewers’ comments are provided in the next sections. We hope these responses are satisfactory and that the revised version will be acceptable for publication.<br><br>Please do not hesitate to contact us with any questions and we are looking forward to your reply.<br><br>Thanks and Best wishes!<br><br>Yours sincerely,<br>YujingSuo and Jianmin Fu<br><br><br>Response to Reviewer:<br><br>Reviewer: The authors complemented their study with a number of comparative genome analyses which indeed provide some, but limited novel biological insights. Some points from the first review round should be addressed or at least there should be someexplanation why these issues are not relevant:<br><br>Q1. transcriptome data: I appreciate the addition of the paragraph on extraction, library construction and sequencing but I still wonder why these data are not used in the analysis, eg. to establish transcription levels for gene or gene families of interest. It seems that the data may be of good quality, multiple tissues etc but there is no statistics or data description anywhere. Will/is the transcriptome data deposited in some public archive?<br><br>A: This article focuses on the genome sequencing, assembly, annotation of D. oleifera, and comparative genomic analyses with other species. The purpose of this article is to provide accurate genomic information for the studies of molecular mechanisms

underlying the formation and regulation of important economic traits in Diospyros spp. Thus, the transcriptome data is mainly used for genomic annotation. Additionally, the transcriptome data is also used to analyze the expression patterns of CHS genes in different tissues of D. oleifera. (Revised manuscript，Page 12，Line288-291).
The transcriptome data is deposited in NCBI, you can find the data under this link: https://dataview.ncbi.nlm.nih.gov/object/PRJNA532832?reviewer=gbss3pp9p06h5hosks3vrepirg

Q2.Phylogeny: how about the bias possibly introduced by just picking the single copy orthologs for the construction of the phylogenetic tree? This set is just a very small subset of the full gene content. To me lines 260/261, and thus construction of the phylogeny, are largely unclear.
A: Gene families were generated by Orthofinder. After clustering, 19,722 gene families were detected across D.oleifera and 11 other species, of which 5,599 gene families and 221 single-copy orthologs were shared by 12 species. A phylogenetic tree of the 12 plant species was constructed using Orthofinder based on phylogenetic tree constructed by FastME. Gene trees were inferred for each orthogroup by aligning the sequences using mafft-linsi and inferring a maximum likelihood tree from this alignment using FastTree. DLCpar was used to reconcile these gene trees with the known species tree. In addition, 221 single-copy orthologs were used to estimate divergence time, rather than construct the phylogenetic tree.

Q3. Gene families: I'm not sure whether there is any biological conclusion on the genes and enrichments that were identified as D.oleifera specific? Can the terms be related to any biological features?
A: Using GO term enrichment analysis, we performed functional annotation on the D.oleifera specific genes. As a result, only 98 of the 312 genes had conserved functional terms which were significantly enriched for zinc ion bingding, proteolysis, and nutrient reservoir activity. Moreover, 4 and 1 of these genes were involved in the carbohydrate metabolic process and aldehyde metabolic process respectively, which may play roles in the carbohydrate accumulation and deastringency of fruit in D.oleifera.

Q4. Expansion/Contraction: what parameters where used for CAFÉ? CHS expansion results should be outlined in the text. What does "different degrees of expansion" mean? An obvious additional and worthwhile analysis would be check expanded/contracted gene families for their expression patterns. What is the conclusion of LAC gene family contraction?
A: (1) For CAFÉ parameter Settings: Gene families with size significantly changed for species/branch: viterbi $p<=0.05$, and the others are the default parameters.
(2) The description of these results has been revised as follow: compared with D. lotus, C. sinensis, and V. vinifera, chalcone synthase (CHS) genes expanded in the D. oleifera genome (11 genes in D.oleifera, 7 genes in D.lotus, 3 genes in C. sinensis, and 1 gene in V. vinifera; P_value = 0.0089). In addition, Using transcriptome data, CHS gene expression patterns in different tissues of persimmon were analyzed. (Revised manuscript，Page 12，Line 284-291)
(3) Laccase (LAC) genes were responsible for the polymerization of persimmon tannin monomers. The contraction of these genes may explain the difference of tannin types which were defined according to the polymerization level of tannin monomers between D. oleifera and V. vinifera.

Q5. Positively selected genes: I'm really not sure about the significance of this analysis. Are the terms identified somewhat related to any biological features?
A: Positively selection analysis was used to study the adaptive evolution of genes, which could help us better understand the evolution of D.oleifera. In this study, 186 genes were positively selected in D. oleifera compared with D. lotus, A. chinensis, P. veris, R. delavayi and S. lycopersicum. Among them, chalcone isomerase (CHI) gene, a key enzyme in the flavonoid-anthocyanin pathway, was found to be positively selected (ID:evm.model.original_scaffold_909.101). The positive selection of CHI gene may be one of the reasons why D. oleifera is different from other species in producing abundant tannin. (Revised manuscript，Page13，Line 305-307)

Powered by Editorial Manager® and ProduXion Manager® from Aries Systems Corporation

	Q6. Please check the formats and structure of your files provided. Testing the GFF files with Gff3Validator results in an error for example:gt gff3validator Dol.gff3gt gff3validator: error: child on line 44626 in file Dol.gff3" has different sequence id than its parent on line 44625 ('Chr4' vs. 'fragScaff_scaffold_95:::fragment_2:::debris')<br>A: Thank you for pointing this out, we have checked the formats and structure of our files, and corrected the error. The revised files have been re-uploaded to the system.<br><br>Q7.Especially the newly added text needs significant improvement in language and grammar.<br>A: The English in this revised manuscript has been checked by at least two professional editors, both native speakers of English. For a certificate, please see: http://www.textcheck.com/certificate/FrAnnY
**Additional Information:**	
**Question**	**Response**
Are you submitting this manuscript to a special series or article collection?	No
**Experimental design and statistics**<br><br>Full details of the experimental design and statistical methods used should be given in the Methods section, as detailed in our Minimum Standards Reporting Checklist. Information essential to interpreting the data presented should be made available in the figure legends.<br><br>Have you included all the information requested in your manuscript?	Yes
**Resources**<br><br>A description of all resources used, including antibodies, cell lines, animals and software tools, with enough information to allow them to be uniquely identified, should be included in the Methods section. Authors are strongly encouraged to cite Research Resource Identifiers (RRIDs) for antibodies, model organisms and tools, where possible.<br><br>Have you included the information requested as detailed in our Minimum Standards Reporting Checklist?	Yes
**Availability of data and materials**	Yes

Powered by Editorial Manager® and ProduXion Manager® from Aries Systems Corporation

All datasets and code on which the conclusions of the paper rely must be either included in your submission or deposited in publicly available repositories (where available and ethically appropriate), referencing such data using a unique identifier in the references and in the "Availability of Data and Materials" section of your manuscript.<br><br>Have you have met the above requirement as detailed in our Minimum Standards Reporting Checklist?	

Manuscript

Click here to view linked References

Click here to access/download;Manuscript;174-Revised Manuscript-HL.doc

A high-quality chromosomal genome assembly of *Diospyros oleifera*

Yujing Suo[1,2,3,†], Peng Sun[1,2,3,†], Huihui Cheng[4†], Weijuan Han[1,2,3], Songfeng Diao[1,2,3], Huawei Li[1,2,3], Yini Mai[1,2,3], Xing Zhao[4], Fangdong Li[1,2,3,∗] and Jianmin Fu[1,2,3,∗]

[1]Key Laboratory of Non-timber Forest Germplasm Enhancement & Utilization of State Administration of Forestry and Grassland, No.3 Weiwu Road, Jinshui District, Zhengzhou, 450003, China; [2]Non-timber Forest Research and Development Center, Chinese Academy of Forestry, No.3 Weiwu Road, Jinshui District, Zhengzhou, 450003, China; [3]National Innovation Alliance of Persimmon Industry, No.3 Weiwu Road, Jinshui District, Zhengzhou, 450003, China; [4]Novogene Bioinformatics Institute, Beijing, 100083, China

∗ Correspondence address. Fangdong Li, Non-timber Forest Research and Development Center, Chinese Academy of Forestry, No.3 Weiwu Road, Jinshui District, Zhengzhou, 450003, E-mail: lifangdong66@163.com; Jianmin Fu, Non-timber Forest Research and Development Center, Chinese Academy of Forestry, No.3 Weiwu Road, Jinshui District, Zhengzhou, 450003, E-mail: fjm371@163.com

[†]Contributed equally to this work.

ORCIDs:

Yujing Suo, 0000-0002-1080-5094
Peng Sun, 0000-0001-5746-9684
Huihui Cheng, 0000-0003-3461-9381
Weijuan Han, 0000-0002-2032-3398
Songfeng Diao, 0000-0002-9690-6290
Huawei Li, 0000-0003-3734-6569
Yini Mai, 0000-0003-0849-9451
Xing Zhao, 0000-0001-5474-6850
Fangdong Li, 0000-0003-3447-3714
Jianmin Fu, 0000-0002-7368-0751

Abstract

Background: *Diospyros oleifera*, a member of the genus *Diospyros* of the family Ebenaceae, is an economically important tree. Phylogenetic analyses have indicated that *D. oleifera* is closely related to *D. kaki*, and could be used as a model plant for studies of *D. kaki*. Therefore, development of genomic resources of *D. oleifera* will facilitate auxiliary assembly of the hexaploid persimmon genome and provide insight into the molecular mechanisms of major economic traits. **Findings:** The *D. oleifera* genome was assembled into 443.6 Gb of raw reads using the Pacific Bioscience Sequel and Illumina HiSeq X Ten platforms. The final draft genome was approximately 812.3 Mb and had a high level of continuity with the 3.36 Mb N50. Using the Hi-C data and the draft genome assembly, 15 scaffolds corresponding to the 15 chromosomes were assembled to a final

size of 721.5 Mb using 332 scaffolds, accounting for 88.81% of the genome. The identified repeat sequences accounted for 54.8% of the genome. By *de novo* sequencing and analysis of homology with other plant species, 30,530 protein-coding genes with an average transcript size of 7,105.40 bp were annotated; of these, 28,580 protein-coding genes (93.61%) had conserved functional motifs or terms. In addition, 171 candidate genes involved in the tannin synthesis and deastringency in persimmon were identified; of these chalcone synthase (*CHS*) genes expanded in the *D. oleifera* genome compared with *D. lotus*, *C. sinensis*, and *V. vinifera*. Moreover, 186 positively selected genes were identified, including chalcone isomerase (*CHI*) gene, which is a key enzyme in the flavonoid-anthocyanin pathway. Phylogenetic tree analysis indicated that the split of *D. oleifera* and *D. lotus* likely occurred 9.0 million years ago. In addition to the ancient γ event, a second whole-genome duplication event occurred in *D. oleifera* and *D. lotus* (4dtv = 0.36 ~ 0.27-0.42).

Conclusions: We generated a high-quality chromosome-level draft genome for *D. oleifera*, which will facilitate assembly of the hexaploid persimmon genome and further studies of major economic traits in the genus *Diospyros*.

Keywords: *Diospyros oleifera*; chromosome-level genome assembly; Hi-C assembly; Tannin synthesis; sex determination genes

Data Description

Background

Diospyros is the largest genus in the family Ebenaceae, comprising more than 500 species, of which the ebony and fruit have considerable economic value. The ebony of more than 20 species of *Diospyros* (including *D. reticulata* from Africa, *D. ebenum* and *D. ferrea* from Asia) is used commercially for arts, crafts, and decorative building materials. In addition, *D. kaki*, *D. oleifera*, and *D. lotus* are important species for fruit production; indeed, *D. kaki* is one of the most widely

distributed fruit trees worldwide. However, most *D. kaki* cultivars are hexaploid (2n = 6× = 90) or nonaploid (2n = 9× = 135) and their progenitor, origin, and polyploidization mechanisms are unclear, which has hampered molecular breeding. *D. oleifera* is diploid (2n = 2× = 30) and its fruit contains large quantities of tannins, important raw materials for the production of persimmon paint (Fig. 1). *D. oleifera* is also frequently selected as stock for grafting of persimmon (*D. kaki*). Phylogenetic analyses based on the chloroplast genome and protein-coding, intergenic, and intron sequences have indicated that *D. oleifera* is closely related to *D. kaki* and could be used as a model plant for studies of *D. kaki* [1]. Therefore, analysis of the genome of *D. oleifera* will contribute to auxiliary assembly of the hexaploid persimmon genome.

The cultivars of hexaploid *D. kaki* are classified into four groups based on the mode of astringency loss by the fruits: pollination-constant non-astringent (PCNA), pollination-variant non-astringent (PVNA), pollination-constant astringent (PCA), and pollination-variant astringent (PVA) [2]. PCNA is the most desirable type because the fruits are edible without any postharvest treatment. Due to the complexity of the hexaploid *D. kaki* genome and the lack of genomic sequence information, the natural deastringency mechanism of China-PCNA (CPCNA) *D. kaki* is still unclear. Therefore, this study, which will identify tannin synthesis related genes based on genomic sequence information in *D. oleifera*, will be helpful for subsequent studies on natural deastringency mechanisms of CPCNA *D. kaki*.

The sexuality of *Diospyros spp.* is diverse. For example, diploid *D. lotus* is dioecious, including gynoecious (bearing only female flowers) and androecious (bearing only male flowers) types, whereas both diploid *D. oleifera* and hexaploid *D. kaki* contain gynoecious, androecious, monoecious (bearing both female and male flowers), polygamomonoecious (bearing female, male and hermaphroditic flowers), and andromonoecious (bearing male and hermaphroditic flowers) types. The sexuality of *D. oleifera* and *D. kaki* is also labile. For example, gynoecious *D. oleifera* and *D. kaki* trees may occasionally bear male flowers [3]. The mechanism underlying such sexual

diversity and lability in diploid *D. oleifera*, in contrast to the dioecy of diploid *D. lotus* deserves further investigation; greater understanding of this mechanism will also help to uncover the complicated mechanism of sexual diversity and lability in *D. kaki*. The genomic sequence imformation of *D. oleifera* obtained in the present study will be valuable for studies on sexuality in *D. oleifera* and other *Diospyros spp*.

Here, we performed a high-quality chromosome-level reference genome assembly for *D. oleifera* (NCBI:txid227308) using the long reads generated by the Pacific Biosciences (PacBio) DNA sequencing platform and Hi-C data. The high quality (in terms of completeness and continuity) of the genome will facilitate both assembly of the hexaploid persimmon genome and further studies of major economic traits in the genus *Diospyros*.

Genomic DNA extraction, library construction, sequencing, and genome size estimation

Genomic DNA was extracted from fresh leaves of *D. oleifera* using a DNAsecure Plant Kit (Tiangen Biotech, Beijing, China). A short-read genomic library was prepared using the TruSeq DNA PCR-Free LT Library Kit (Illumina, San Diego, CA, USA). Five paired-end genomic sequence libraries with a gradient insert size of 250 to 450 bp were constructed and sequenced on the Illumina HiSeq X Ten platform. A total of 104.02 Gb of raw sequence data (119.78-fold coverage of the *D. oleifera* genome) were used for genome assembly (Supplementary Table S1).

At least 10 μg of sheared DNA was required to generate the 40 kb insert library. Preparation of the SMRT cell template involved DNA concentration, damage repair, end repair, ligation of hairpin adapters, and template purification. Subsequently, the genome was sequenced on the PacBio Sequel platform (Pacific Biosciences, Menlo Park, CA, USA). A total of 99.76 Gb of raw sequence data (114.88-fold coverage of the *D. oleifera* genome) were used for genome assembly (Supplementary Table S1).

To produce a 10X genome library, about 1 ng of input DNA (50 kb length) was used for the GEM reaction during PCR, and 16 bp barcodes were introduced into droplets. Next, the droplets

were fractured following purification of the intermediate DNA library. The library comprised 109.88 Gb (126.53-fold coverage of the *D. oleifera* genome) and was sequenced using 150 bp paired-end reads on the Illumina HiSeq X platform (Supplementary Table S1).

One Dovetail Hi-C library was prepared as described previously [4]. Briefly, for each library, chromatin was fixed in place in the nucleus using formaldehyde and then extracted. Fixed chromatin was digested with *Dpn*II, the 5′ overhangs were filled using biotinylated nucleotides, and free blunt ends were ligated. After ligation, crosslinks were reversed, and DNA was separated from protein. Purified DNA was treated to remove biotin outside of the ligated fragments, sheared to a mean fragment size of about 350 bp, and used to create sequence libraries with NEBNext Ultra enzymes (New England Biolabs, Ipswich, MA, USA) and Illumina-compatible adapters. Biotin-containing fragments were isolated using streptavidin beads before PCR enrichment of the libraries; the libraries were next sequenced on the Illumina HiSeq PE150 platform. A total of 98.24 Gb of reads was produced for the libraries. Together, these Dovetail Hi-C library reads provided 113.12-fold physical coverage of the genome (Supplementary Table S1).

High-quality paired-end reads from *D. oleifera* were used to generate 17-mer frequency information by k-mer analysis [5]. The 17-mer distribution was dependent on the characteristics of the genome and followed a Poisson distribution (Supplementary Fig. S1). We estimated the genome to be 868.41 Mb in size with a heterozygosity of 1.08% (Supplementary Table S2).

De novo* assembly of *D. oleifera

De novo assembly of the long reads generated by SMRT sequencing was performed using FALCON [6,7] (v.0.3; Falcon, RRID:SCR_016089). Briefly, we first selected the longest coverage of subreads as seeds for error correction. Next, the data were filtered and assembled (length_cutoff_pr = 4000, max_diff = 100, and max_cov = 100). A total of 2,986 contigs was assembled with a total length of 806.74 Mb (accounting for about 92.9% of the estimated genome), an N50 of 2.92 Mb, and a longest contig of 14.72 Mb (Table 1). The primary contigs

(p-contigs) were polished using Quiver [8] by aligning SMRT reads, which produced a genome of 812.37 Mb and an N50 of 2.94 Mb. Finally, Pilon [9] (v.1.22; Pilon, RRID:SCR_014731) was used to perform the second round of error correction with the short paired-end reads generated by the Illumina HiSeq platform, resulting in a genome of 811.09 Mb and a longest contig of 14.81 Mb (Table 1). For the scaffolding step, Long Ranger (v. 2.1.2,) [10] was applied to build scaffolds using the 10X data. FragScaff [11] (v. 1-1) was used to build superscaffolds from the barcoded sequencing reads. The final assembly contained 2,812 scaffolds and had a total length of 812.32 Mb, representing approximately 93.54% of the genome estimated by k-mer analysis. The sizes of the longest contig and scaffold were 14.82 and 17.43 Mb, respectively, and the N50s were 2.94 and 3.36 Mb, respectively (Table 1). Subsequently, the Hi-C sequencing data were aligned to the assembled scaffolds by BWA-mem [12] (v.0.7.8), and the scaffolds were clustered onto chromosomes with LACHESIS (LACHESIS, RRID:SCR_017644)[13]. Among the 2,812 scaffolds, 332 were grouped into the 15 chromosomes, with maximum and minimum lengths of 61.45 Mb and 40.21 Mb, respectively (Fig. 2). The final genome was 721.45 Mb and the N50 was 33.5 Mb, accounting for 88.81% of the total genome (Supplementary Table S3, Fig. 3). The continuity and integrity of the assembly for *D. oleifera* is significantly better than that of the published *D. lotus* genome, which final genome was 945.63 Mb with contigs N50 0.65Mb, and 746.09 Mb (78.9%) was assembled into the 15 pseudomolecules[14].

Assessment of the assembled genome

To estimate the quality of the assembled genome, the short reads were mapped back to the consensus genome using BWA (BWA, RRID:SCR_010910); the overall mapping rate was 98.19%, suggesting that the assembly contained comprehensive genomic information (Supplementary Table S4). The completeness of gene regions was assessed using Core Eukaryotic Gene Mapping Approach (CEGMA, RRID:SCR_015055) [15] and Benchmarking Universal Single-Copy Orthologs (BUSCO, RRID:SCR_015008) [16] to evaluate the completeness of the assembled genome. The CEGMA

assessment showed that 215 (93.55%) of 248 core eukaryotic genes were covered (Supplementary Table S5). In contrast, 89.4% of complete BUSCOs were detected and 6.6% were missing (Supplementary Table S6), indicating that the assembled genome had a high level of completeness.

Repetitive elements identification of *D. oleifera*

The *D. oleifera* genome was subjected to annotation of repetitive sequences—transposable elements (TEs) and tandem repeats. RepeatMasker [17,18] (v.4.0.5; RepeatMasker, RRID:SCR_012954) was used to detect TEs in a repeat library derived from a known repeat library (Repbase, v. 15.02) and a *de novo* repeat library generated using RepeatModeler [4] (v. 1.0.5; RepeatModeler, RRID:SCR_015027), RepeatScout [19] (v.1.0.5; RepeatScout, RRID:SCR_014653), Piler (v.1.0; PILER, RRID:SCR_017333), and LTR_FINDER [20] (v.1.0.7; LTR_Finder, RRID:SCR_015247). RepeatProteinMask [18] (v.4.0.5) was used to detect TEs in the *D. oleifera* genome by comparison with a TE database. Tandem repeats were identified using Tandem Repeats Finder [21] (v.4.0.7).

Repetitive sequences comprised 54.8% of the *D. oleifera* genome, among which TEs accounted for 53.03%. The most frequently detected TEs were long terminal repeat (LTR) retrotransposons (46.73%), followed by DNA TEs (4.17%). Of the LTRs, 26.63% and 14.40% were Ty3/Gypsy and Ty1/Copia, respectively (Table 2).

Genomic RNA extraction, library construction, sequencing

For RNA-sequencing, we collected different tissues of *D.oleifera* from the same plant used for genome sequencing, including material from leaf, root, seed, stem and fruit. Total RNAs were extracted using TRIzol® Reagent (Thermo Fisher Scientific, USA) according to the manufacturer's instructions. RNA-seq was performed using an Illumina platform.

Annotation of protein-coding genes

De novo, homolog-based, and RNA sequencing (RNA-seq)-based predictions were employed to annotate the protein-coding genes in the *D. oleifera* genome. The following *ab initio* gene prediction software packages were used to predict genes: Augustus [22, 23] (v. 3.0.2; Augustus,

RRID:SCR_008417), Genescan [24] (v. 1.0; GENSCAN, RRID:SCR_012902), Geneid [25] (v.1.4), GlimmerHMM [26] (v. 3.0.2; GlimmerHMM, RRID:SCR_002654), and SNAP [27] (SNAP, RRID:SCR_007936; 2013-11-29). The protein sequences of seven species (including *Arabidopsis thaliana* and *Daucus carota*) were downloaded from Ensembl or the National Center for Biotechnology Information (NCBI) databases. Homologous sequences were aligned against the repeat-masked *D. oleifera* genome using TBLASTN [28] (v.2.2.26, E-value ≤ 1E-05; TBLASTN, RRID:SCR_011822). Genewise [29] (v. 2.2.0) was employed to predict gene models based on the aligned sequences. The RNA-seq data were assembled into the unique sequences of transcripts by mapping the RNA-seq data to the *D. oleifera* genome using TopHat [30] (v. 2.0.8; TopHat, RRID:SCR_013035) and Cufflinks [31, 32] (v. 2.1.1; Cufflinks, RRID:SCR_014597) for transcript assembly. Alternatively, Trinity [33] (v.2.1.1; Trinity, RRID:SCR_013048) was used to assemble the RNA-seq data, and the gene structures were improved using PASA [34, 35] software (r20140417; PASA, RRID:SCR_014656). A weighted and non-redundant gene set was generated by merging all of the gene models predicted by the above three approaches with EVidenceModeler (EVM, RRID:SCR_014659) [36] (v.1.1.1). PASA was applied to adjust the gene models generated by EVM. The final reference gene set contained 30,530 protein-coding genes with an average transcript size of 7,105.4 bp, an average coding sequence size of 1,080.74 bp, and a mean number of exons per gene of 4.62 (Supplementary Table S7). The number of annotated genes in this genome is less than that in the *D. lotus* genome (40,532 genes).

Functional annotation

Functional annotation of protein-coding genes was performed according to the best BLAST hit by BLASTP (v.2.2.28, E-value ≤ 1E-05; BLASTP, RRID:SCR_001010) searching of the SwissProt, TrEMBL [37], and NCBI non-redundant (NR) protein databases. Motifs and domains were annotated by searching the Pfam, PRINTS, PROSITE, ProDom, and SMART InterPro (v. 29.0) databases using InterProScan [38] (v. 4.8; InterProScan, RRID:SCR_005829). The Gene Ontology term for

each gene was annotated by Blast2GO (Blast2GO, RRID:SCR_005828) [39]. Additionally, the gene sets were mapped to Kyoto Encyclopedia of Genes and Genomes (KEGG) [40] (v. 53) pathways to identify the best match classification for each gene (BLASTp E-value ≤ 1E-05). Finally, 28,580 protein-coding genes (93.61% of total 30,530 genes) had conserved functional motifs or functional terms—92.03% (28,098), 84.16% (25,695), and 71.21% (21,739) of the genes in NR, InterPro, and KEGG, respectively (Supplementary Table S8).

Annotation of non-coding RNAs

tRNA genes were predicted using tRNAscan-SE software [41] (v.1.4; tRNAscan-SE, RRID:SCR_010835) with the default parameters. rRNAs were annotated based on their level of homology with the rRNAs of several species of higher plants (not shown) using BLASTN with an E-value of 1e-5. The miRNA and snRNA fragments were identified by searching the Rfam database (v. 11.0) using INFERNAL [42,43] (v.1.1; Infernal, RRID:SCR_011809) software. Finally, 564 miRNAs, 507 tRNAs, 2,207 rRNAs, and 803 snRNAs were identified, which had average lengths of 114.69, 74.82, 161.40, and 111.54 bp, respectively (Supplementary Table S9).

Identification of tannin synthase genes in *D. oleifera*

Given the importance of tannin production in *D. oleifera,* we identified genes within the tannin biosynthesis pathway, which include the chorismic acid pathway, phenylpropane metabolic pathway, flavonoid-anthocyanin pathway, and proanthocyanidin specific pathway (Supplementary Fig. S1). All of the synthase genes involved in the four pathways, as well as several closely related transcription factors (TFs) including WD40 and WIP-ZF were identified by aligning to reference genes downloaded from the National Center for Biotechnology Information (NCBI) [44] or The Arabidopsis Information Resource (TAIR) [45] using Blastp (E-value<1e-5, identity≥50%, and coverage≥50%). A Pfam HMMER search was used to filter genes that did not contain the corresponding domain. TFs including MYB, MYC, and WRKY were identified and classified into

different families using the iTAK pipeline (v.1.7) [46]. As a result, 171 genes and 380 TFs were identified, of which 13, 59, and 21 genes were involved in the phenylpropane metabolic pathway, flavonoid-anthocyanin pathway, and proanthocyanidin specific pathway, respectively. We also detected 18 genes encoding transport proteins such as glutathione S-transferase (GST) and multi-drug and toxic compound extrusion transporter (MATE), which were closely related to transmembrane transport of tannin. We identified the key genes of acetaldehyde metabolism, such as *ADH* (10), *ALDH* (19), and *PDC* (5), which were related to deastringency in persimmon (Supplementary Table S10). The tannin synthase genes and TFs identified in this study will provide a basis for molecular breeding of persimmon tannins.

Gene family cluster, phylogenetic tree construction, and divergence time estimation

Ten other sequenced plant species were used to investigate the evolution of *D. oleifera*, including eight asterids (*D. lotus*, *Primula veris*, *Rhododendron delavayi*, *Camellia sinensis*, *Actinidia chinensis*, *Daucus carota*, *Coffea canephora*, and *Solanum lycopersicum*) and 3 rosids plants (*A. thaliana*, *Vitis vinifera*, and *Cucumis melo*). Gene families were generated by Orthofinder [47, 48] (v.2.3.1). First, nucleotide and protein data of 10 species were downloaded from Ensembl (Release 70) and NCBI. Before executing an “all against all” BLASTP (E-value ≤ 1E-07) program, the longest transcript was selected from alternatively spliced transcripts of one gene, and genes with ≤ 50 amino acids were removed. The alignments with high-scoring segment pairs were conjoined for each gene pair by SOLAR (Sorting Out Local Alignment Results; V0.0.19) [49]. After clustering, 19,722 gene families were detected in *D. oleifera* and 11 other species, of which 5,599 gene families and 221 single-copy orthologs were shared by 12 species. Among the 5 Ericales species (*D. oleifera*, *D. lotus*, *A. chinensis*, *R. delavayi*, and *C. sinensis*), 177 gene families consisting of 312 genes were unique to *D. oleifera* (Supplementary Fig. S3). Go enrichment analysis of these genes indicated that 98 genes had conserved functional terms which were significantly enriched in GO term of zinc ion

binding, proteolysis, and nutrient reservoir activity. In addition, 4 and 1 of these genes were involved in the carbohydrate metabolic process and aldehyde metabolic process respectively, which may play roles in the carbohydrate accumulation and deastringency of fruit in *D. oleifera*.

A phylogenetic tree of the 12 plant species was constructed using Orthofinder (OrthoFinder, RRID:SCR_017118) based on phylogenetic tree constructed by FastME [50] (v.2.1.5). Gene trees were inferred for each orthogroup by aligning the sequences using mafft-linsi and inferring a maximum likelihood tree from this alignment using FastTree (FastTree, RRID:SCR_015501). DLCpar was used to reconcile these gene trees with the known species tree. Then, the mcmctree program of PAML [51, 52] (v.4.5; PAML, RRID:SCR_014932) was applied to estimate divergence time among 12 species using the 221 shared single-copy orthologs with main parameters burn-in=100,000, sample-number=100,000, and sample-frequency=2. 4 calibration points were selected from the TimeTree website [53]as normal priors to restrain the age of the nodes. The phylogenetic tree confirmed the grouping of Angiospermae. The split of *D. oleifera* and *D. lotus* was estimated at 9.0 million years ago (Mya) (Fig.4).

Expansion and contraction of gene families

We determined the expansion and contraction of the gene families by comparing the cluster size differences between the ancestor and each species using the CAFÉ program [54]. For parameter Settings: Gene families with size significantly changed for species/branch: viterbi p<=0.05, and the others are the default parameters. A random birth and death model was used to study changes in gene families along each lineage of the phylogenetic tree. A probabilistic graphical model was introduced to calculate the probability of transitions in gene family size from parent to child nodes in the phylogeny. Using conditional likelihoods as the test statistics, we calculated the corresponding p-value for each lineage; a p-value of 0.05 was used to identify families that were significantly expanded and contracted. Compared with the common ancestor of *D. oleifera* and *D. lotus*, 175 gene

families (1,896 genes) have expanded in *D. oleifera* (Supplementary Fig. S4); these were enriched in several KEGG pathways including ubiquitin mediated proteolysis, ABC transporters, carbon fixation in photosynthetic organisms. By contrast, 333 gene families (1,021 genes) were contracted in *D. oleifera*; these were enriched in several KEGG pathways including plant−pathogen interaction, phenylpropanoid biosynthesis, cyanoamino acid metabolism.

In addition, the reference sequences of tannin synthase genes identified in *D. oleifera* were used for a blast query to identify homologous genes in *D. lotus*, *C. sinensis*, and *V. vinifera*. A sequence with similarity greater than the cutoff (50%) and coverage greater than the cutoff (50%) was selected as a preliminary candidate gene, followed by searching for the domain using hmmsearch. When the query was identical with that in the subject, the candidate gene was retained. Fisher's exact test (p-value $\leq$ 0.05) was performed on the number of individual genes related to tannin synthesis in the genomes of the four species to see whether the corresponding gene expanded or contracted. Compared with *D. lotus, C. sinensis, and V. vinifera,* chalcone synthase (*CHS*) genes expanded in the *D. oleifera* genome (11 genes in *D. oleifera*, 7 genes in *D. lotus*, 3 genes in *C. sinensis*, and one gene in *V. vinifera*; *P-value* = 0.0089) (Supplementary Table S11). CHS is the first key enzyme in the flavonoid-anthocyanin pathway; expansion of the *CHS* gene may be related to the abundant tannin production in *D. oleifera*. In addition, the expression of *CHS* gene in different tissues of *D. oleifera* was analyzed using transcriptome data. The result showed that the expression of *CHS* genes were spatiotemporal specific, with 3 genes highly expressed in leaves, 6 in roots and 1 in seeds (Supplementary Fig. S5). A contraction of laccase (*LAC*) genes that were responsible for the polymerization of persimmon tannin monomers [55, 56], was observed in *D. oleifera* compared with *V. vinifera* (21 *LAC* genes in *D. oleifera* and 53 in *V. vinifera*). This phenomenon may explain the difference of tannin types which were defined according to the polymerization level of tannin monomers between *D. oleifera* and *V. vinifera*.

Positively selected genes in *D. oleifera*

To understand the evolution of *D. oleifera*, positively selection analysis was performed to study the adaptive evolution of genes. The coding sequence (CDS) alignments of 789 single-copy gene families in *D. oleifera*, *D. lotus*, *A. chinensis*, *P. veris*, *R. delavayi* and *S. lycopersicum* were generated using MUSCLE (MUSCLE, RRID:SCR_011812). Gblocks (Gblocks, RRID:SCR_015945) [57] was applied to filter poorly aligned positions and divergent regions of the CDS alignments. With *D. oleifera* as the foreground branch, positive selection sites were detected based on branch-site models [58] of PAML [51] using the CDS alignments. P-values were computed using the χ^2 statistic and adjusted by FDR method. Finally, 186 genes were positively selected in *D. oleifera* (Supplementary Table S12). Among them, chalcone isomerase (*CHI*) gene, a key enzyme in the flavonoid-anthocyanin pathway, was found to be positively selected (ID:evm.model.original_scaffold_909.101). The positive selection of *CHI* gene may be one of the reasons why *D. oleifera* is different from other species in producing abundant tannin.

Whole-genome duplication and macrosynteny analysis

We used BLASTP (E-value < 1e-5) to perform homolog and paralog searches with *D. oleifera* and other genomes (*A. chinensis, C. canephora, C. sinensis*), and MCScanX (s = 5, e = 1e-5) [59] was used to detect syntenic blocks. Then, transversion substitutions at fourfold degenerate sites (4dtv) rates for all syntenic genes were calculated to identify putative whole genome duplication or species split events in *D. oleifera*. In addition to the ancient whole-genome duplication (WGD) event that occurred in all dicot species, γ event (all core eudicots share an ancient WGD, 4dtv = 0.66), a second WGD event occurred in *D. oleifera* and *D. lotus* (4dtv = 0.36 ~0.27-0.42) that might have contributed to the divergence of Ebenaceae with *A. chinensis* and *C. sinensis* (Fig. 5). We obtained 431 syntenic blocks between *D. olerfera* and *D. lotus*. On the whole, except for the translocation of some loci, the sequence of genes between *D. olerfera* and *D. lotus* was relatively conservative (Supplementary Fig. S6). However, compared with the *D. olerfera* genome, the *D. lotus* genome

lacked some regions on each chromosome, that may have been lost in the process of anchoring contigs to 15 pseudo-chromosomes using genetic maps. This result further demonstrated the integrity and accuracy of *D. olerfera* genome assembly.

Conclusions

We generated a high-quality chromosome-level draft genome of *D. oleifera* based on long reads generated by the third-generation PacBio Sequel sequencing platform. The final draft genome was approximately 812.3 Mb, slightly smaller than the 868.4 Mb estimated by k-mer analysis. The Hi-C data were combined with the assembled draft genome to generate chromosome-length scaffolds. As a result, 15 scaffolds corresponding to the 15 chromosomes were assembled; these comprised 721.5 Mb and 332 scaffolds, with an N50 of 33.5 Mb, and accounted for 88.81% of the genome. A total of 30,530 protein-coding genes were predicted, and 93.61% (28,580 genes) of all protein-coding genes were annotated. Also, repeat sequences accounted for 54.8% of the genome, and 564 miRNAs, 507 tRNAs, 2,207 rRNAs, and 803 snRNAs were identified. In addition, 171 candidate genes involved in tannin synthesis and deastringency were identified; of these, *CHS* genes expanded in the *D. oleifera* genome compared with *D. lotus*, *C. sinensis*, and *V. vinifera*. Moreover, 186 positively selected genes were identified, including *CHI* gene, a key enzyme in the flavonoid-anthocyanin pathway. The divergence time between *D. oleifera* and *D. lotus* was estimated at 9.0 Mya, and two WGD events occurred in the *D. oleifera* genome. The high-quality chromosomal genome assembly of *D. oleifera* will facilitate both research on the major economic traits in the genus *Diospyros* and assembly of the hexaploid persimmon genome.

Editors Note

Please also note another genome assembly of *Diospyros oleifera* has been published during the data curation and acceptance of this paper[60].

Availability of supporting data

Raw sequencing data is available in the NCBI Sequence Read Archive [Accessions: PRJNA532832], and assemblies, annotations, alignments, expression data and BUSCO/CEGMA results are available from the *GigaScience* database GigaDB [61].

Additional files

Fig. S1. k-mer distribution of the *D. olefera* genome.

Fig. S2. Tannin synthesis genes and the deastringency process in *Diospyros*.

Fig. S3. Venn diagram of gene family clusters of five Ericales species.

Fig. S4. Gene family expansion and contraction analysis of 12 species.

Fig. S5. Expression of *CHS* genes in different tissues of *D. oleifera*.

Fig. S6. Macrosynteny analysis between the *D. olefera* genome and the *D. lotus* genome. a: gene density; b: LINE transposon density; c: LTR transposon density; d: DNA transposon density; and e: GC density (density is calculated in units of 500 kb).

Table S1. Sequencing data size by various sequencing platforms.

Table S2. Estimation of *D. oleifera* genome size by k-mer analysis.

Table S3. Chromosome lengths using HiC reads.

Table S4. Mapping rate of reads to *D. oleifera* genome assembly.

Table S5. CEGMA assessment of the *D. oleifera* genome.

Table S6. BUSCO notation assessment of the *D. oleifera* genome.

Table S7. Gene annotation of the *D. oleifera* genome via three methods.

Table S8. *D.oleifera* genome gene annotation statistics using different databases.

Table S9. *D.oleifera* genome ncRNA annotation statistics using different databases.

Table S10. Genes involved in tannin synthesis genes and the deastringency process in *D. oleifera*.

Table S11. Expansion and contraction of tannin synthase genes in *D. oleifera*.

Table S12. Positively selected genes in *D. oleifera.*

Abbreviations

BWA: Burrows-Wheeler Aligner; BLAST: Basic Local Alignment Search Tool; BUSCO: Benchmarking Universal Single-Copy Orthologs; NCBI: National Center for Biotechnology Information; PacBio: Pacific Biosciences; RNA-seq: RNA-sequencing; SMRT sequencing: single molecule real time sequencing; TE: transposable element; WGD: whole-genome duplication.

Competing interests

The authors declare that they have no competing interests.

Funding

This work was supported by the National Key R &D Program of China (2018YFD1000606), and the Fundamental Research Funds for the Central Non-profit Research Institution of CAF (CAFYBB2017ZA005 and CAFYBB2017ZA004-3)

Author contributions

J.M.F. and F.D.L. conceived the project. W.J.H., H.W.L. and S.F.D. collected the samples, Y.J.S., P.S. and Y.N.M. conducted genome assembly and data analysis, X.Z. provided intellectual insights, Y.J.S., P.S. and H.H.C. wrote the paper. All authors read and wrote part of the manuscript.

The English in this document has been checked by at least two professional editors, both native speakers of English. For a certificate, please see: http://www.textcheck.com/certificate/FrAnnY

References

1. Fu JM, Liu HM, Hu JJ, et al. Five Complete Chloroplast Genome Sequences from *Diospyros*: Genome Organization and Comparative Analysis. PLOS ONE, 2016, 11(7):e0159566-.
2. Yonemori, K., Sugiura, A., Yamada, M. Persimmon genetics and breeding. In: Janick, J. (Ed.), Plant Breeding Reviews, 2000, vol. 19. John Wiley & Sons, Inc., pp. 191–225.
3. Yakushiji H, Ymada M, Yonenori K, et al. Staminate flower production on shoots of ‘Fuyu’ and ‘Jiro’ persimmon (*Diospyros kaki* Thunb.). J Jpn Soc Hortic Sci, 1995, 64(1):41-46.
4. Lieberman-Aiden E, et al. Comprehensive mapping of long-range interactions reveals folding principles of the human genome. Science, 2009, 326: 289–293.
5. Liu, B. et al. Estimation of genomic characteristics by analyzing k-mer frequency in de novo genome projects. arXiv: Genomics, 2013.
6. Chin CS, Peluso P, Sedlazeck FJ, et al. Phased diploid genome assembly with single-molecule real-time sequencing. Nature Methods, 2016.
7. Github. https://github.com/PacificBiosciences/FALCON/.
8. Chin CS, Alexander DH, Marks P, et al. Nonhybrid, finished microbial genome assemblies from long-read SMRT sequencing data. Nature Methods, 2013, 10(6):563.
9. Walker BJ, Abeel T, Shea T, et al. Pilon: An Integrated Tool for Comprehensive Microbial Variant Detection and Genome Assembly Improvement. PLOS ONE, 2014, 9.
10. Long Ranger. https://support.10xgenomics.com/genome-exome/software/pipelines/latest/installation.
11. Adey A, Kitzman JO, Burton JN, et al. In vitro, long-range sequence information for de novo genome assembly via transposase contiguity. Genome Research, 2014, 24(12):2041-2049.
12. Li H, and Durbin R. Fast and accurate short read alignment with Burrows-Wheeler transform. Bioinformatics, 2009.
13. LACHESIS zGithub. http://shendurelab.github.io/LACHESIS/.

14. Akagi T, Shirasawa K, Nagasaki H, et al. The persimmon genome reveals clues to the evolution of a lineage-specific sex determination system in plants. bioRxiv 628537; doi: https://doi.org/10.1101/628537
15. Parra G, Bradnam K, Korf I. CEGMA: a pipeline to accurately annotate core genes in eukaryotic genomes. Bioinformatics, 2007, 23(9):1061-1067.
16. Simao FA, Waterhouse RM, Ioannidis P, et al. BUSCO: assessing genome assembly and annotation completeness with single-copy orthologs. Bioinformatics, 2015:btv351.
17. Smit AF, Hubley R, Green P. RepeatMasker at <http://www.repeatmasker.org/RepeatModeler.html>.
18. Chen, N. Using RepeatMasker to identify repetitive elements in genomic sequences. *Curr Protoc* Bioinformatics, 2004, Chapter 4, Unit 4.
19. Price AL, Jones NC, Pevzner PA . De novo identification of repeat families in large genomes. Bioinformatics, 2005, 21(Suppl 1):i351-i358.
20. Xu Z, Wang H. LTR_FINDER: an efficient tool for the prediction of full-length LTR retrotransposons. Nucleic Acids Research, 2007, 35(Web Server):W265-W268.
21. Benson G. Tandem repeats finder: a program to analyze DNA sequences. Nucleic Acids Res. 1999, 27, 573-580.
22. Stanke M, Waack S. Gene prediction with a hidden Markov model and a new intron submodel. Bioinformatics, 2003, 19, ii215–25.
23. Stanke M, Sch?Ffmann O, Morgenstern B, et al. Gene prediction in eukaryotes with a generalized hidden Markov model that uses hints from external sources. Bmc Bioinformatics, 2006, 7(1):62-0.
24. Salamov AA. Ab initio gene finding in Drosophila genomic DNA. Genome Res, 2000, 10, 516–522.
25. Parra G, Blanco E, Guigó R. GeneID in Drosophila. Genome Research, 2000, 10(4):511.

26. Majoros WH, Pertea M, Salzberg SL. TigrScan and GlimmerHMM: two open source ab initio eukaryotic gene-finders. Bioinformatics, 2004, 20(16):2878-2879.
27. Korf I. Gene finding in novel genomes. BMC Bioinformatics, 2004, 5, 59.
28. Altschul SF, et al. Gapped BLAST and PSI-BLAST: a new generation of protein database search programs. Nucleic Acids Res, 1997, 25, 3389-402.
29. Birney E, Clamp M, Durbin R. Genewise and genomewise. Genome Res, 2004, 14, 988–995.
30. Trapnell C, Pachter L, Salzberg SL. TopHat: Discovering splice junctions with RNA-Seq. Bioinformatics, 2009, 25, 1105-11.
31. Trapnell C, Williams BA, Pertea G, et al. Transcript assembly and quantification by RNA-Seq reveals unannotated transcripts and isoform switching during cell differentiation. Nature Biotechnology, 2010, 28(5):511-515.
32. Cufflinks Github. http://cole-trapnell-lab.github.io/cufflinks/.
33. Grabherr MG, Haas BJ, Yassour M, et al. Full-length transcriptome assembly from RNA-Seq data without a reference genome. Nature Biotechnology, 2011, 29(7):644-652.
34. Haas BJ, Delcher AL, Mount SM, et al. Improving the Arabidopsis genome annotation using maximal transcript alignment assemblies. Nucleic Acids Research, 2003, 31(19):5654-5666.
35. Pasapipeline Github. http://pasapipeline.github.io/.
36. Haas BJ, Salzberg SL, Zhu W, et al. Automated eukaryotic gene structure annotation using EVidenceModeler and the Program to Assemble Spliced Alignments. Genome biology, 2008, 9(1).
37. Bairoch A, Apweiler R. The SWISS-PROT protein sequence database and its supplement TrEMBL in 2000. Nucleic Acids Res, 2000, 28, 45-48.
38. Mulder N, Apweiler R. InterPro and InterProScan: tools for protein sequence classification and comparison. Methods Mol Biol, 2007, 396, 59-70.
39. Ashburner M, Ball CA, Blake JA, et al. Gene ontology: tool for the unification of biology. The

Gene Ontology Consortium. Nat Genet, 2000, 25, 25-29.

40. Kanehisa M, Goto S. KEGG: kyoto encyclopedia of genes and genomes. Nucleic Acids Res, 2000, 28, 27-30.

41. Lowe TM, Eddy SR. tRNAscan-SE: A Program for Improved Detection of Transfer RNA Genes in Genomic Sequence. Nucleic Acids Res, 1997, 25, 955-964.

42. Nawrocki EP, Kolbe DL, Eddy SR. Infernal 1.0: inference of RNA alignments. Bioinformatics, 2009, 25(10):1335.

43. Nawrocki EP, Eddy SR. Infernal 1.1: 100-fold faster RNA homology searches. Bioinformatics, 2013, 29(22):2933-2935.

44. NCBI. https://www.ncbi.nlm.nih.gov.

45. TAIR. https://www.arabidopsis.org/index.jsp.

46. iTAK. http://itak.feilab.net/cgi-bin/itak/index.cgi.

47. Emms DM, Kelly S. OrthoFinder: solving fundamental biases in whole genome comparisons dramatically improves orthogroup inference accuracy. Genome Biology, 2015, 16(1):157.

48. OrthoFinder. http://www.stevekellylab.com/software/orthofinder.

49. Yu XJ, Zheng HK, Wang J, et al. Detecting lineage-specific adaptive evolution of brain-expressed genes in human using rhesus macaque as outgroup. Genomics, 2006, 88(6):0-751.

50. Lefort V, Desper R, Gascuel O. FastME 2.0: A Comprehensive, Accurate, and Fast Distance-Based Phylogeny Inference Program: Table 1. Molecular Biology and Evolution, 2015:msv150.

51. Yang Z. PAML 4: Phylogenetic Analysis by Maximum Likelihood. Mol. Biol. Evol. 2007, 24: 1586-1591.

52. PAML. http://abacus.gene.ucl.ac.uk/software/paml.html.

53. TimeTree. http://www.timetree.org.

54. De Bie T, Cristianini N, Demuth JP, & Hahn MW. CAFE: a computational tool for the study of gene family evolution. Bioinformatics,2006, 22(10), 1269-1271.

55. Zhao J，Pang Y，Dixon RA. The mysteries of proanthocyanidin transport and polymerization. Plant Physiology，2010,153 (2)：437–443.

56. Hu QN，Luo C，Zhang QL，Luo ZR. Isolation and characterization of a laccase gene potentially involved in proanthocyanidin polymerization in oriental persimmon（*Diospyros kaki* Thunb.）fruit. Molecular Biology Reports，2013, 40 (4)：2809–2820.

57. Castresana J. Selection of conserved blocks from multiple alignments for their use in phylogenetic analysis. Molecular Biology and Evolution,2000, 17: 540-552.

58. Zhang J, Nielsen R, Yang Z. Evaluation of an Improved Branch-Site Likelihood Method for Detecting Positive Selection at the Molecular Level. Mol Biol Evol.,2005, 22, 2472-2479 .

59. Wang Y, Tang H, Debarry JD, et al. MCScanX: a toolkit for detection and evolutionary analysis of gene synteny and collinearity. Nucleic Acids Research, 2012, 40(7):e49-e49.

60. Zhu, Q., Xu, Y., Yang, Y. et al. The persimmon (*Diospyros oleifera* Cheng) genome provides new insights into the inheritance of astringency and ancestral evolution. Hortic Res 6, 138 (2019) doi:10.1038/s41438-019-0227-2

61. Suo Y; Sun P; Cheng H; Han W; Diao S; Li H; Mai Y; Zhao X; Li F; Fu J (2019): Supporting data for "A high-quality chromosomal genome assembly of Diospyros oleifera" *GigaScience* Database. http://dx.doi.org/10.5524/100687.

 Click here to access/download;Table;Tables.xlsx

Table 1 Summary of the *D.oleifera* genome assembly using Pacbio long-read, Illumina reads and 10X genomics data

**Program**	**SampleID**	**Length**		**Number**	
		**Contig(bp)**	**Scaffold(bp)**	**Contig**	**Scaffold**
**Falcon**	Total	806,744,914	-	2,986	-
	N50	2,916,360	-	72	-
**Quiver**	Total	812369941	-	2,986	-
	N50	2,938,972	-	72	-
**Pilon**	Total	811,094,501	-	2986	-
	N50	2,937,127	-	72	-
**10X**	Total	811,094,501	812,323,628	2,986	2,812
	Max	14,814,786	17,432,797	-	-
	Number>=2000	-	-	2,803	2,629
	N50	2,937,127	3,359,874	71	62
	N60	2,314,962	2,662,781	103	89
	N70	1,622,862	1,911,995	144	125
	N80	790,034	1,007,083	214	182
	N90	196,816	257,477	421	333

Table 2 Classification of repetitive elements in *D.oleifera* genome

total repeative elements

**Program**	**Repeat Size(bp)**	**% of genome**
Trf	79,886,467	9.83
Repeatmasker	408,623,327	50.3
Proteinmask	22,154,795	2.73
Total	445,187,963	54.8

Transposons elements

**Type**		**Transposons Elementss Length(bp)**	**% in Genome**
DNA		33,844,732	4.17
LINE		13,187,364	1.62
SINE		74,819	0.01
LTR	total	379,582,766	46.73
	Gypsy	216,328,284	26.63
	Copia	116,970,626	14.40
	other	46,283,856	5.70
Unknown		10,513,280	1.29
Total		430,778,122	53.03

Click here to access/download;Figure;Fig. 1.png

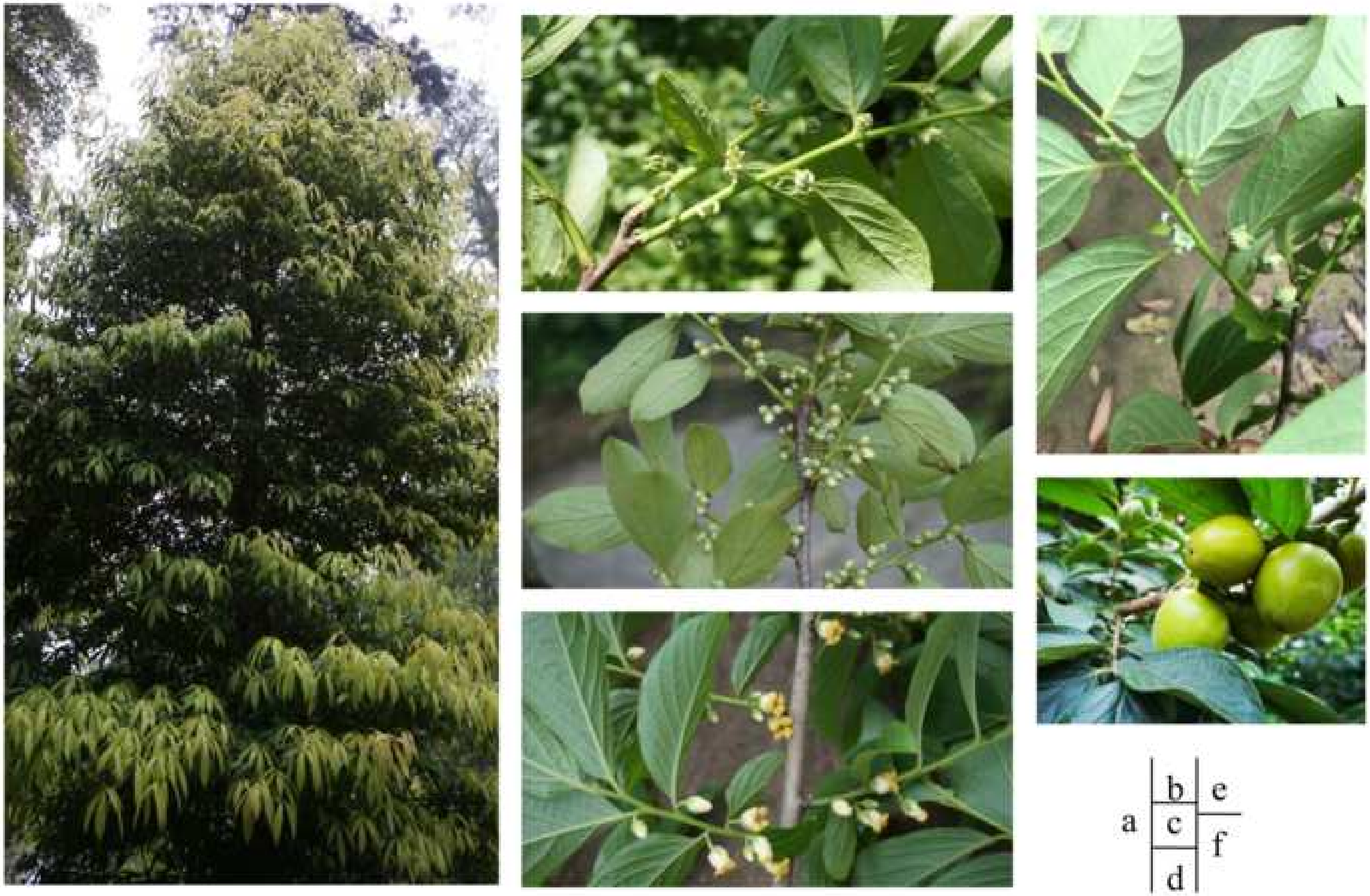

Fig. 2

Click here to access/download;Figure;Fig. 2.png

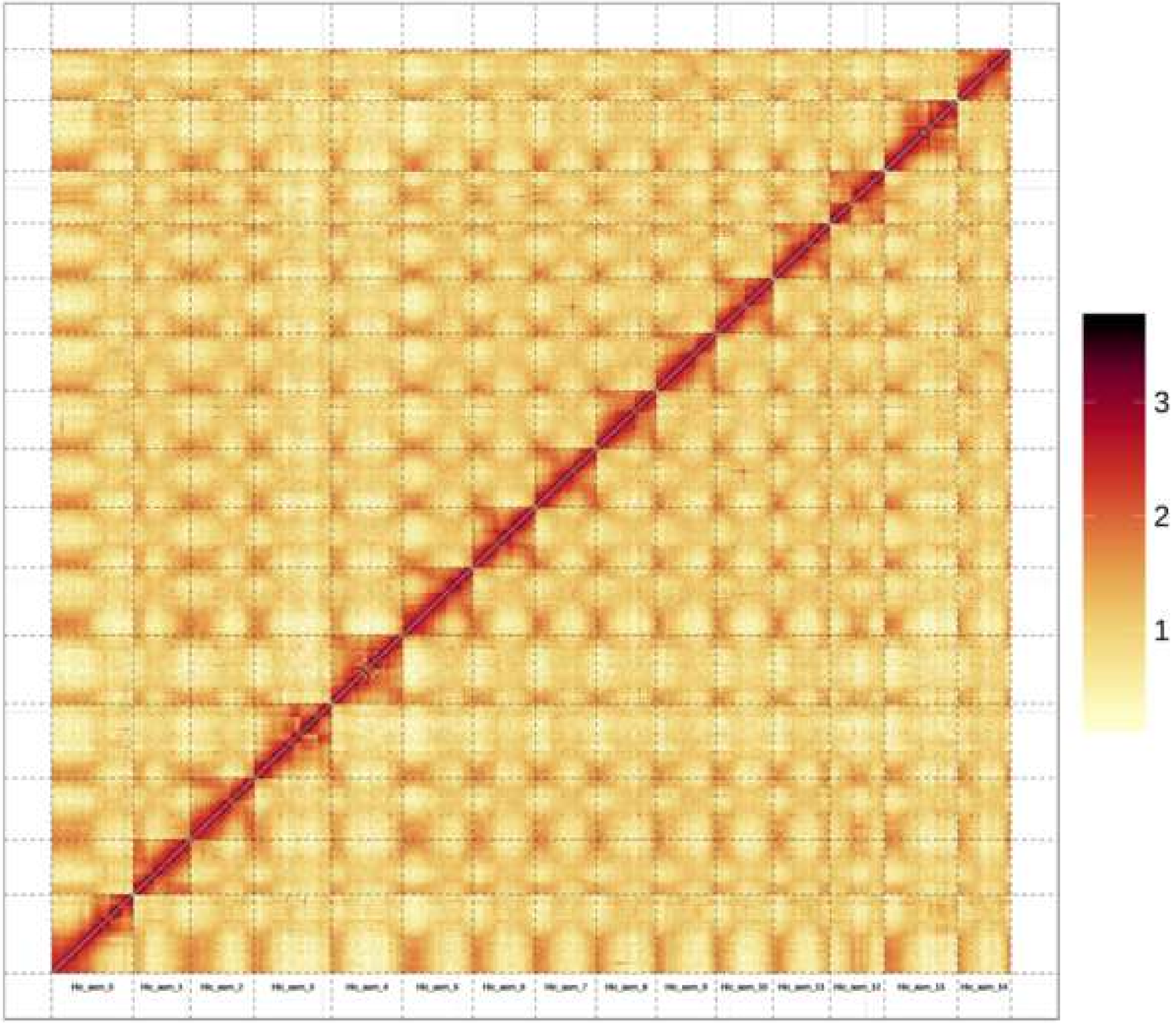

Fig. 3

Click here to access/download;Figure;Fig. 3.png

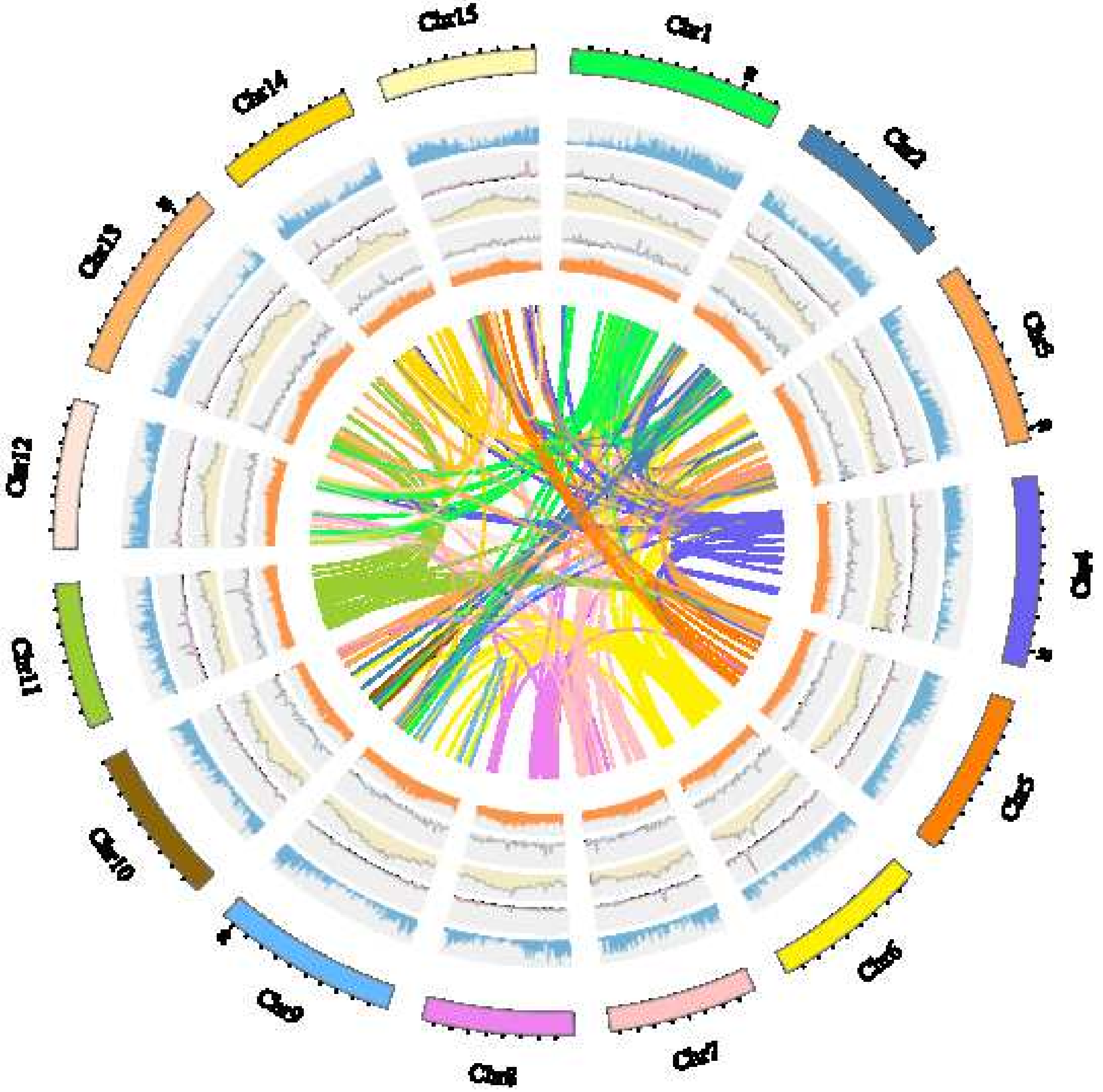

Fig. 4

Click here to access/download;Figure;Fig. 4.png

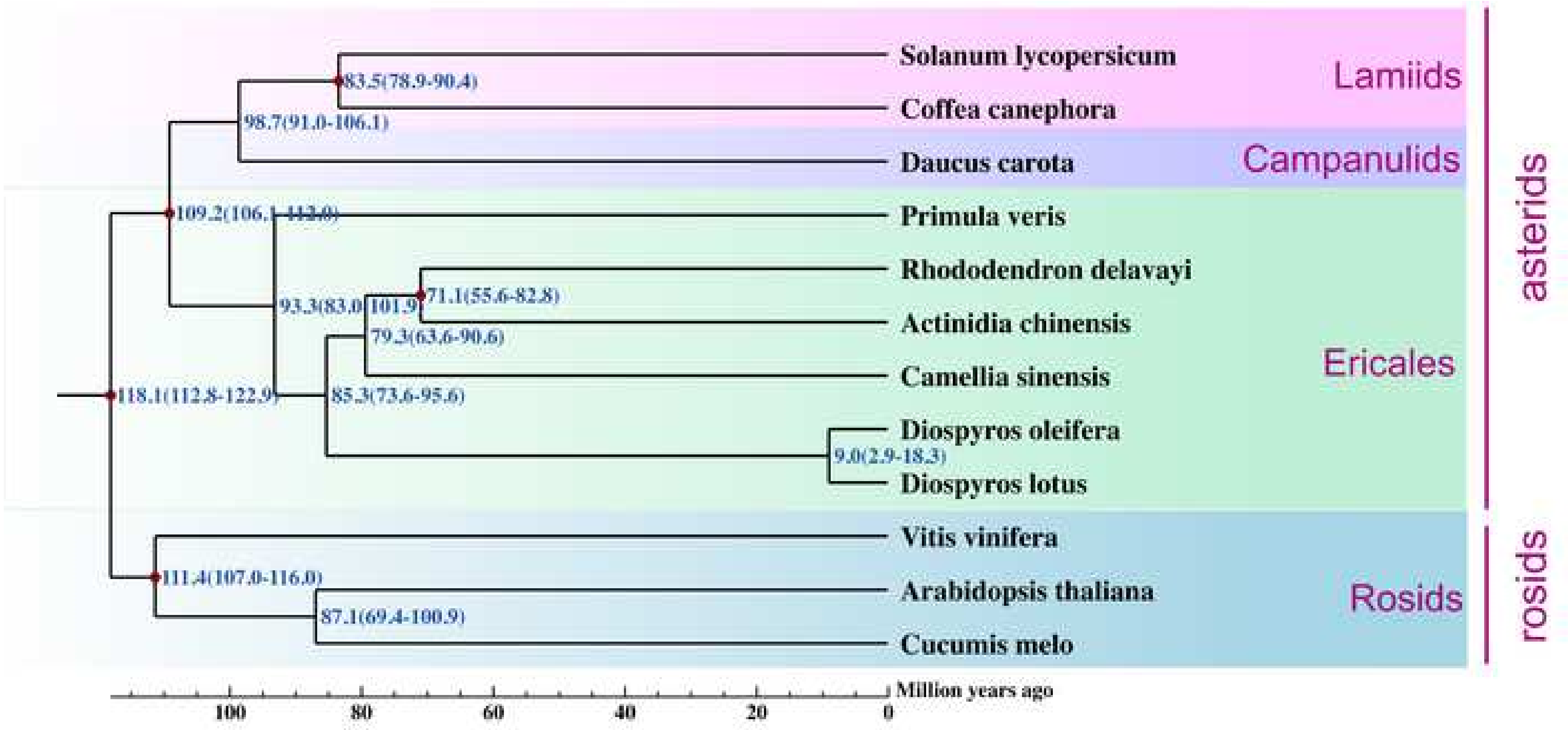

Fig. 5

Click here to access/download;Figure;Fig. 5.png

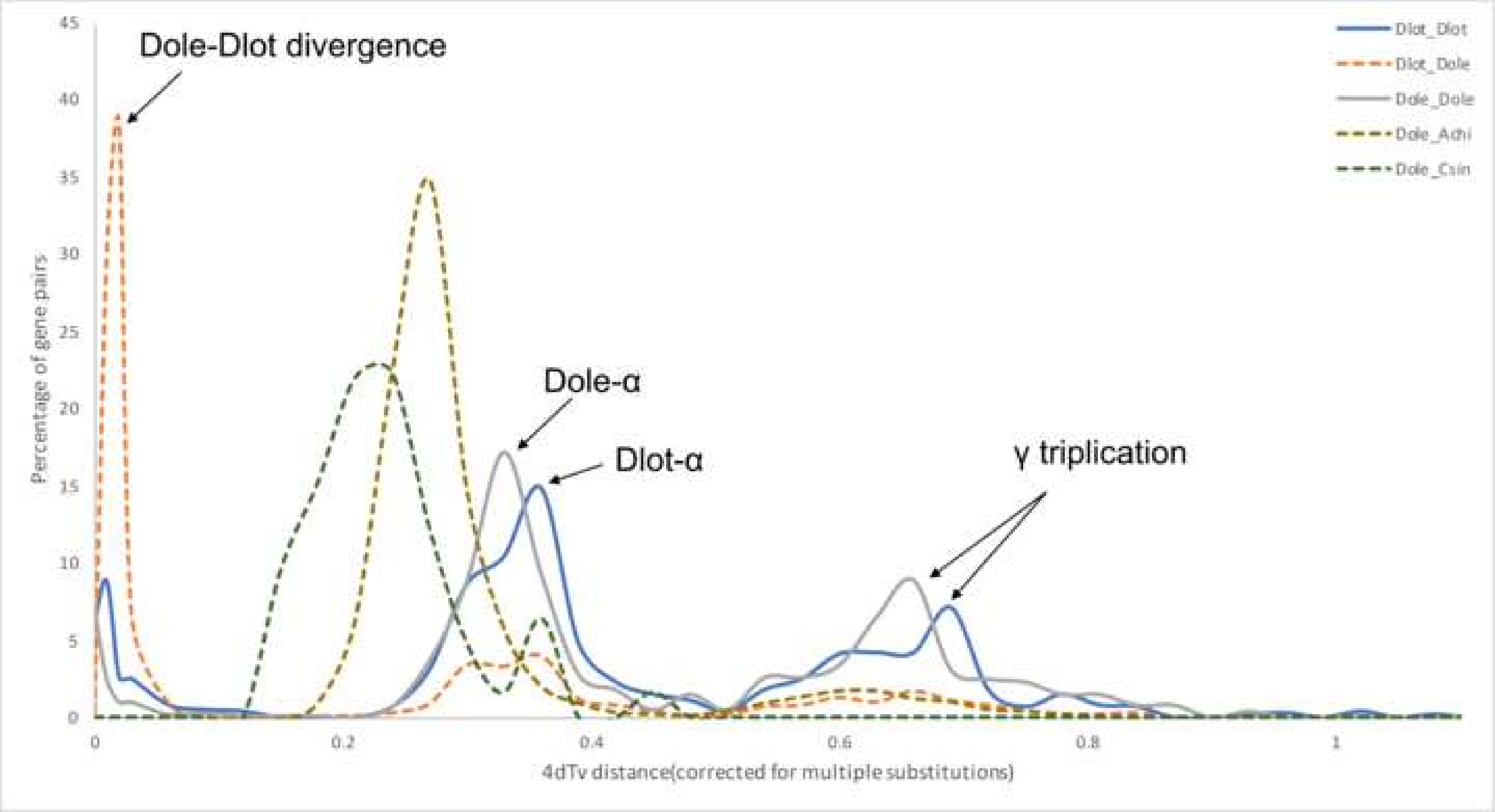

Fig. S1

Click here to access/download
Supplementary Material
Fig. S1.png

Fig. S2

Click here to access/download
Supplementary Material
Fig. S2.png

Fig. S3

Click here to access/download
Supplementary Material
Fig. S3.png

Fig. S4

Click here to access/download
Supplementary Material
Fig. S4.png

Fig. S5

Click here to access/download
Supplementary Material
Fig. S5.png

Fig. S6

Click here to access/download
Supplementary Material
Fig. S6.png

Click here to access/download
Supplementary Material
Supplementary Tables.xlsx

Personal Cover Click here to access/download;Personal Cover;Response to Reviewers.docx

Dear Editor,

Thank you very much for offering us the opportunity to resubmit a revised version of our manuscript. Hereby, we submit the revised manuscript entitled "**A high-quality chromosomal genome assembly of *Diospyrosoleifera***" **(GIGA-D-19-00174)** to **GigaScience**. We appreciate the valuable comments and suggestions from you and the reviewers, which help us to improve and clarify the manuscript. We have discussed the comments carefully and tried our best to improve the manuscript accordingly.

This study focuses on the genome sequencing, assembly and annotation of *D. oleifera*, moreover comparative genomic analyses with other species were also included. The purpose of this study is to provide accurate genomic information for the further studies on molecular mechanisms underlying the formation and regulation of important economic traits of *Diospyros spp*. Based on this present study, some biological issues such as sex differentiation of flowers and natural deastringence of fruits in persimmon are being studied, the results of which will be reported in the future. Additionally, we heard that a similar study has been conducted by another research group, and they have submitted their manuscript to another journal. Due to this information, we deeply appreciate that if the review progress of our manuscript could be accelerated.

Detailed responses to your and the reviewers' comments are provided in the next sections. We hope these responses are satisfactory and that the revised version will be acceptable for publication.

Please do not hesitate to contact us with any questions and we are looking forward to your reply.

Thanks and Best wishes!

Yours sincerely,
YujingSuo and Jianmin Fu

Response to Reviewer:

Reviewer: The authors complemented their study with a number of comparative genome analyses which indeed provide some, but limited novel biological insights. Some points from the first review round should be addressed or at least there should be someexplanation why these issues are not relevant:

Q1. transcriptome data: I appreciate the addition of the paragraph on extraction, library construction and sequencing but I still wonder why these data are not used in the analysis, eg. to establish transcription levels for gene or gene families of interest. It seems that the data may be of good quality, multiple tissues etc but there is no statistics or data description anywhere. Will/is the transcriptome data deposited in some public archive?

A: This article focuses on the genome sequencing, assembly, annotation of *D. oleifera*, and comparative genomic analyses with other species. The purpose of this article is to provide accurate genomic information for the studies of molecular mechanisms underlying the formation and regulation of important economic traits in *Diospyros spp*. Thus, the transcriptome data is mainly used for genomic annotation. Additionally, the transcriptome data is also used to analyze the expression patterns of *CHS* genes in different tissues of *D. oleifera*. (Revised manuscript，Page 12，Line288-291).

The transcriptome data is deposited in NCBI, you can find the data under this link: https://dataview.ncbi.nlm.nih.gov/object/PRJNA532832?reviewer=gbss3pp9p06h5ho sks3vrepirg

Q2.Phylogeny: how about the bias possibly introduced by just picking the single copy orthologs for the construction of the phylogenetic tree? This set is just a very small subset of the full gene content. To me lines 260/261, and thus construction of the phylogeny, are largely unclear.

A: Gene families were generated by Orthofinder. After clustering, 19,722 gene families were detected across *D.oleifera* and 11 other species, of which 5,599 gene families and 221 single-copy orthologs were shared by 12 species. A phylogenetic tree of the 12 plant species was constructed using Orthofinder based on phylogenetic tree constructed by FastME. Gene trees were inferred for each orthogroup by aligning

the sequences using mafft-linsi and inferring a maximum likelihood tree from this alignment using FastTree. DLCpar was used to reconcile these gene trees with the known species tree. In addition, 221 single-copy orthologs were used to estimate divergence time, rather than construct the phylogenetic tree.

Q3. Gene families: I'm not sure whether there is any biological conclusion on the genes and enrichments that were identified as D.oleifera specific? Can the terms be related to any biological features?

A: Using GO term enrichment analysis, we performed functional annotation on the *D.oleifera* specific genes. As a result, only 98 of the 312 genes had conserved functional terms which were significantly enriched for zinc ion bingding, proteolysis, and nutrient reservoir activity. Moreover, 4 and 1 of these genes were involved in the carbohydrate metabolic process and aldehyde metabolic process respectively, which may play roles in the carbohydrate accumulation and deastringency of fruit in *D.oleifera*.

Q4. Expansion/Contraction: what parameters where used for CAFÉ? CHS expansion results should be outlined in the text. What does "different degrees of expansion" mean? An obvious additional and worthwhile analysis would be check expanded/contracted gene families for their expression patterns. What is the conclusion of LAC gene family contraction?

A: (1) For CAFÉ parameter Settings: Gene families with size significantly changed for species/branch: viterbi p<=0.05, and the others are the default parameters.

(2) The description of these results has been revised as follow: compared with *D. lotus, C. sinensis, and V. vinifera*, chalcone synthase (*CHS*) genes expanded in the *D. oleifera* genome (11 genes in *D.oleifera*, 7 genes in *D.lotus*, 3 genes in *C. sinensis*, and 1 gene in *V. vinifera*; P_value = 0.0089). In addition, Using transcriptome data, *CHS* gene expression patterns in different tissues of persimmon were analyzed. (Revised manuscript，Page 12，Line 284-291)

(3) Laccase (LAC) genes were responsible for the polymerization of persimmon tannin monomers. The contraction of these genes may explain the difference of tannin types which were defined according to the polymerization level of tannin monomers between *D. oleifera* and *V. vinifera*.

Q5. Positively selected genes: I'm really not sure about the significance of this analysis. Are the terms identified somewhat related to any biological features?

A: Positively selection analysis was used to study the adaptive evolution of genes, which could help us better understand the evolution of *D.oleifera*. In this study, 186 genes were positively selected in *D. oleifera* compared with *D. lotus*, *A. chinensis*, *P. veris*, *R. delavayi* and *S. lycopersicum*. Among them, chalcone isomerase (*CHI*) gene, a key enzyme in the flavonoid-anthocyanin pathway, was found to be positively selected (ID:evm.model.original_scaffold_909.101). The positive selection of *CHI* gene may be one of the reasons why *D. oleifera* is different from other species in producing abundant tannin. (Revised manuscript，Page13，Line 305-307)

Q6. Please check the formats and structure of your files provided. Testing the GFF files with Gff3Validator results in an error for example: gt gff3validator Dol.gff3 gt gff3validator: error: child on line 44626 in file Dol.gff3" has different sequence id than its parent on line 44625 ('Chr4' vs. 'fragScaff_scaffold_95:::fragment_2:::debris')

A: Thank you for pointing this out, we have checked the formats and structure of our files, and corrected the error. The revised files have been re-uploaded to the system.

Q7.Especially the newly added text needs significant improvement in language and grammar.

A: The English in this revised manuscript has been checked by at least two professional editors, both native speakers of English. For a certificate, please see: http://www.textcheck.com/certificate/FrAnnY
