# Supplementary figures and images for: A high-quality chromosomal genome assembly of Diospyros oleifera Cheng
Source: Gigascience. 2020 Jan 16;9(1):giz164. doi: 10.1093/gigascience/giz164 (PMC6964648; doi:10.1093/gigascience/giz164)

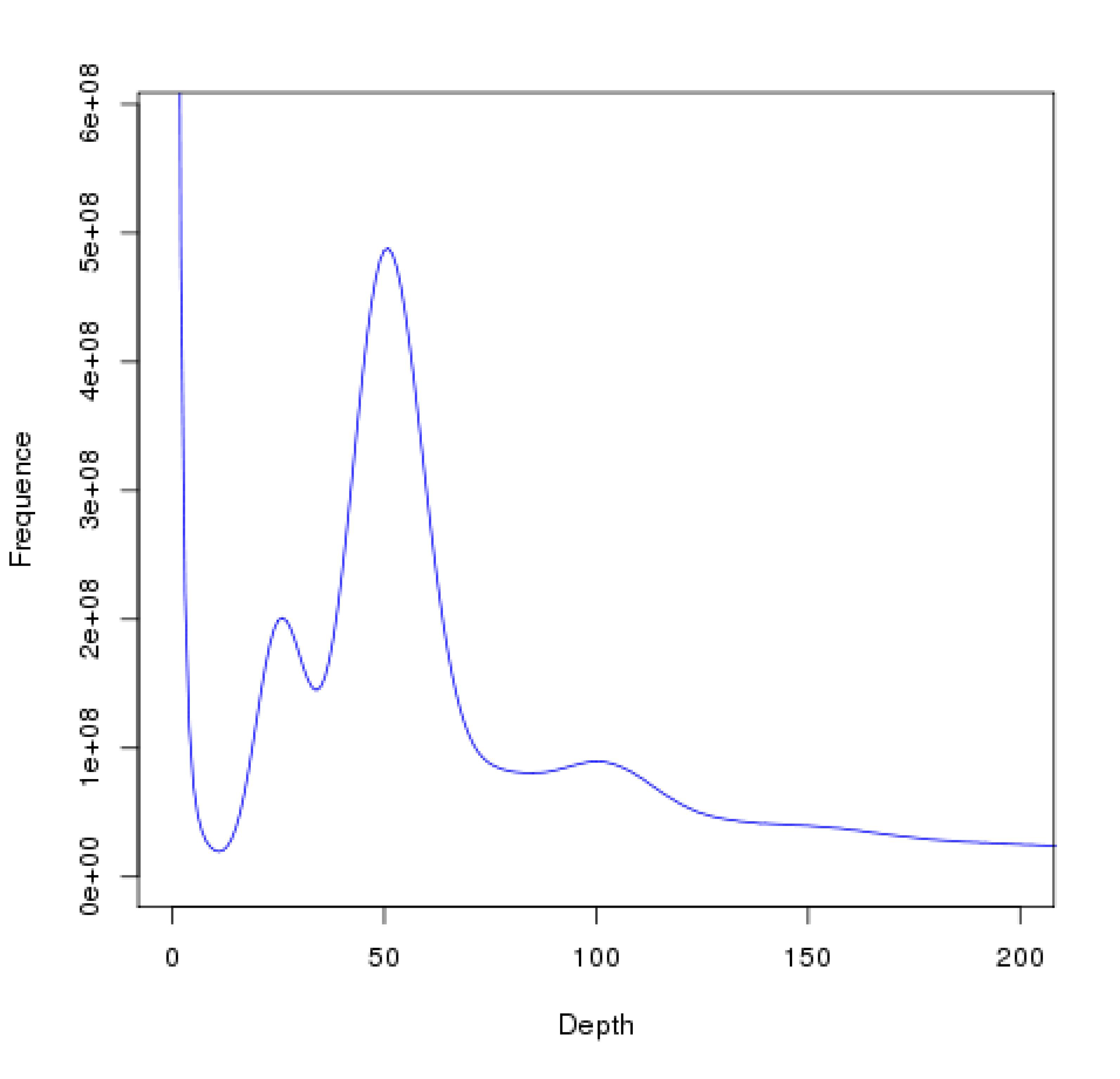

Supplement: giz164_Supplemental_Figures_and_Tables [file giz164_supplemental_figures_and_tables.zip › Fig. S1.png]

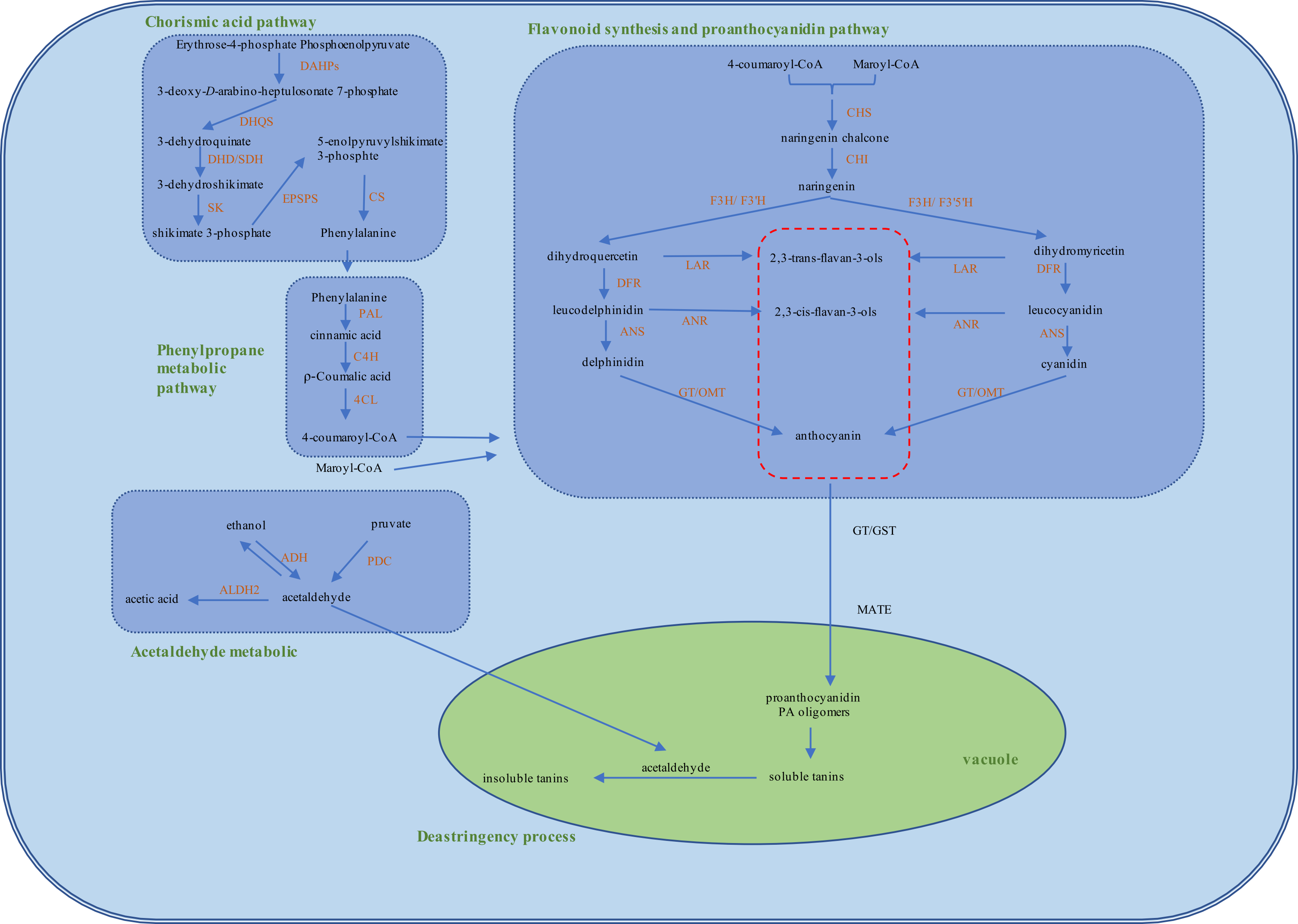

Supplement: giz164_Supplemental_Figures_and_Tables [file giz164_supplemental_figures_and_tables.zip › Fig. S2.png]

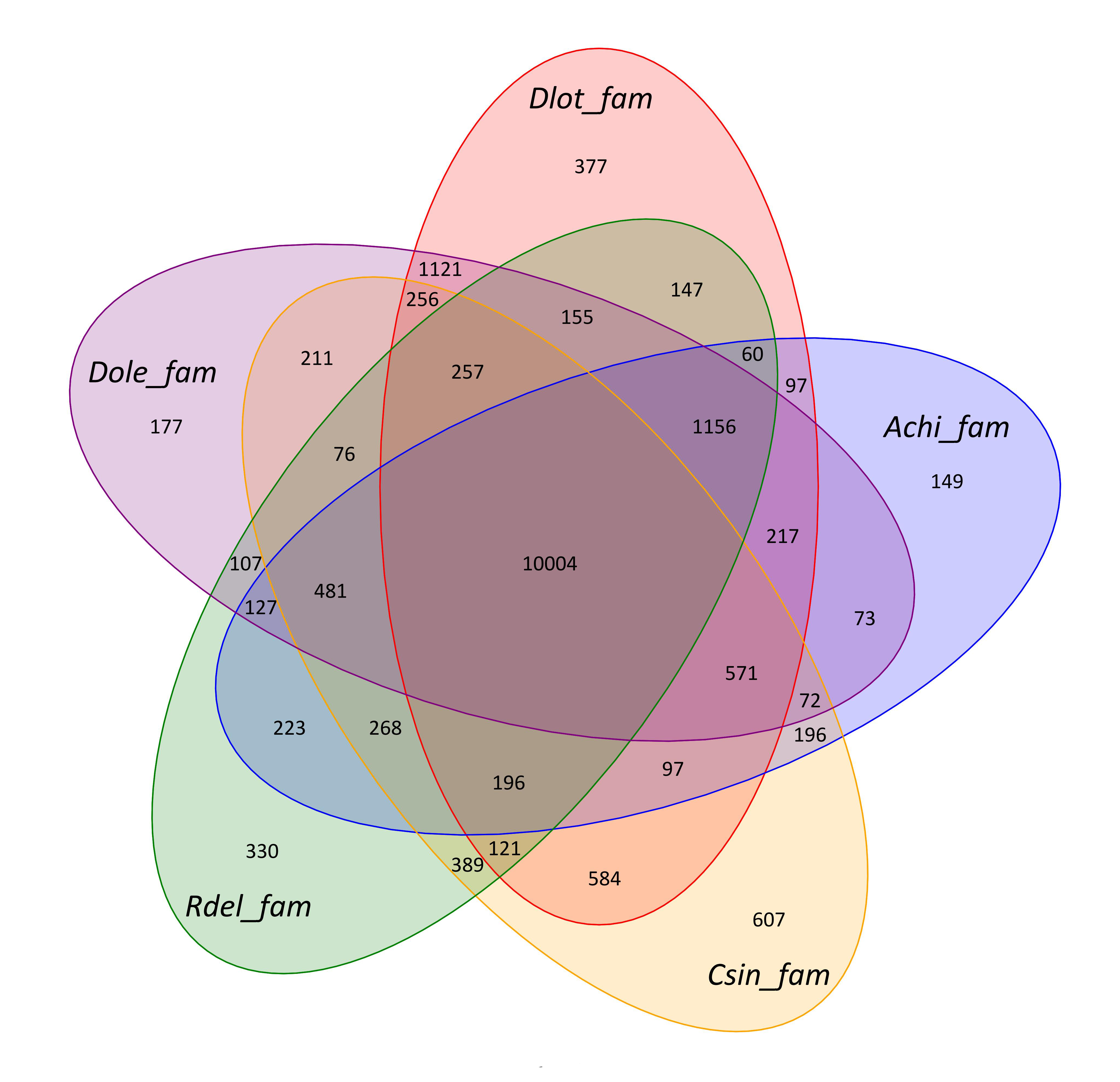

Supplement: giz164_Supplemental_Figures_and_Tables [file giz164_supplemental_figures_and_tables.zip › Fig. S3.png]

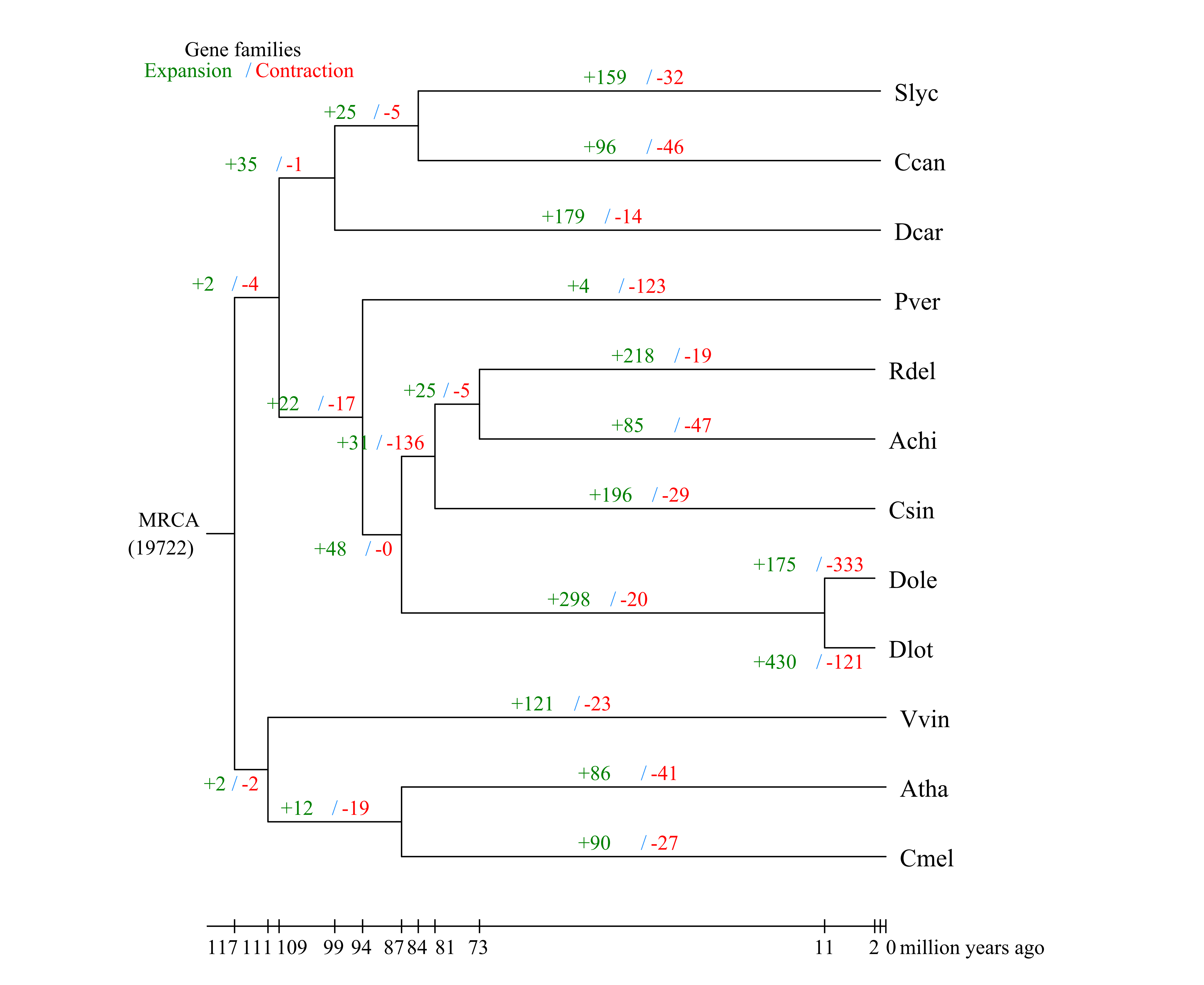

Supplement: giz164_Supplemental_Figures_and_Tables [file giz164_supplemental_figures_and_tables.zip › Fig. S4.png]

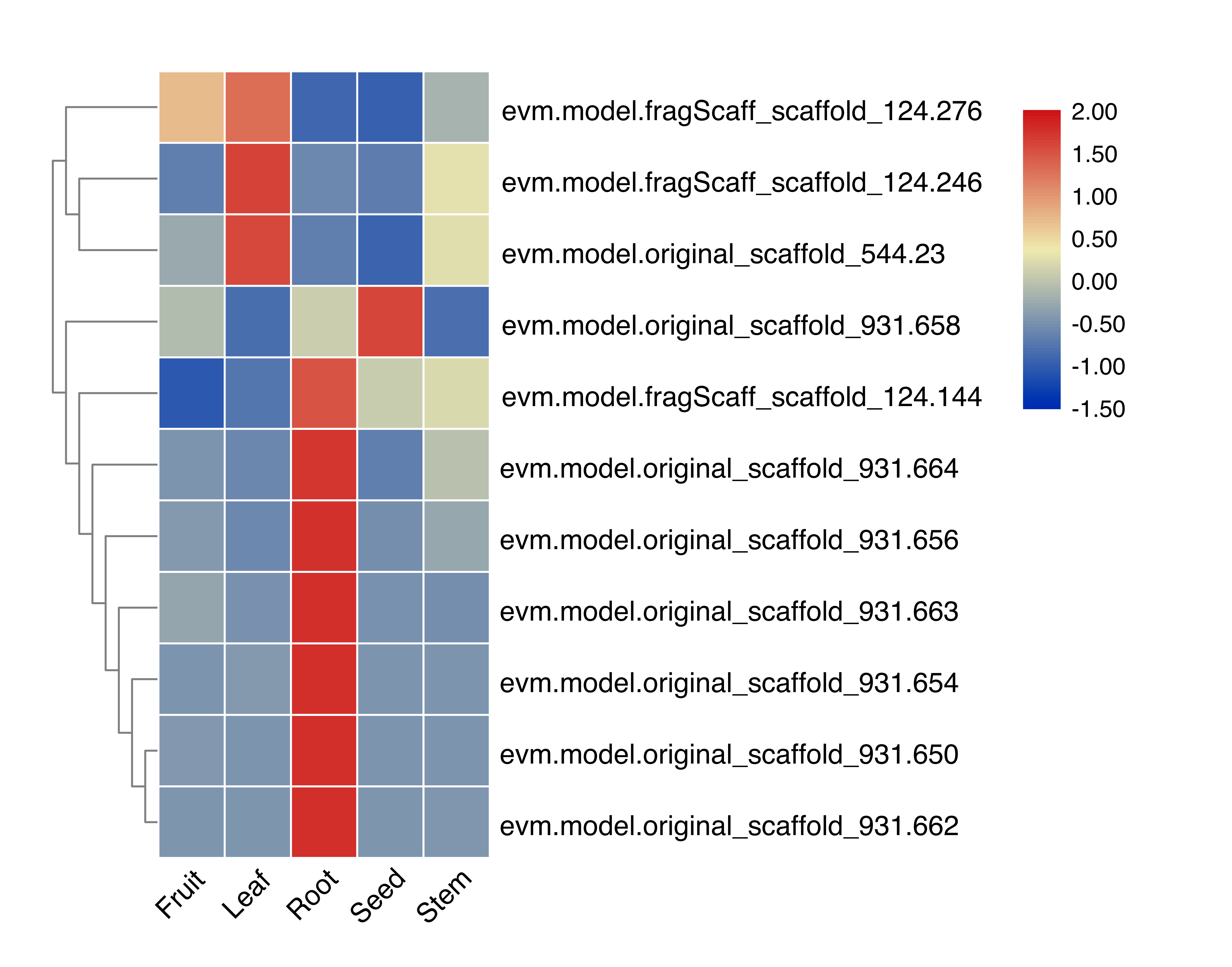

Supplement: giz164_Supplemental_Figures_and_Tables [file giz164_supplemental_figures_and_tables.zip › Fig. S5.png]
