# Supplementary material for: A high-quality chromosomal genome assembly of Diospyros oleifera Cheng
Source: Gigascience. 2020 Jan 16;9(1):giz164. doi: 10.1093/gigascience/giz164 (PMC6964648; doi:10.1093/gigascience/giz164)
Supplement: giz164_Response_to_Reviewer_Comments_Original_Submission [file giz164_response_to_reviewer_comments_original_submission.pdf]

Author's Response To Reviewer Comments

Close

Dear Editor,

Thank you very much for offering us the opportunity to resubmit a revised version of our manuscript. Hereby, we submit the revised manuscript entitled “A high-quality chromosomal genome assembly of Diospyros oleifera” (GIGA-D-19-00174) to GigaScience. We appreciate the valuable comments and suggestions from you and the reviewers, which help us to improve and clarify the manuscript. We have discussed the comments carefully and tried our best to improve the manuscript according to the reviewers’ good comments.

The main modifications are as follows:
1. We confirmed the NCBI data link, you and the reviewers can find the data under this link: https://dataview.ncbi.nlm.nih.gov/object/PRJNA532832?reviewer=gbss3pp9p06h5hosks3vrepirg

2. We supplemented the content of comparative genomic analysis, including gene family cluster, expansion and contraction of gene families, positively selection analysis, and macrosynteny analysis. Some interesting results were found: Firstly, compared with D.lotus, C.sinensis, and V.vinifera, CHS (Chalcone synthase) genes (the first key enzyme in the flavonoid-anthocyanin pathway) had different degrees of expansion in D.oleifera genome, which may be contribute to the abundant tannin production in the D.oleifera. Besides, CHI (Chalcone isomerase) gene (another key enzyme in the flavonoid-anthocyanin pathway) was found to be positive selected. These results will provide important data support for the molecular mechanism analysis of the major economic traits in Diospyros. Furthermore, the divergence time of between D. oleifera and D.lotus was estimated at 9.0 Mya, and two WGD events occurred in D.oleifera genome. These results will be helpful for the further analysis of the evolution of Diospyros species, and the origin of hexaploid persimmon.

Detailed responses to your and the reviewers’ comments are provided in the next sections. We hope you and the reviewers will be satisfied with our responses to the comments and the revisions for the original manuscript. Please feel free to contact us with any questions and we are looking forward to your response.

Thanks and Best wishes!

Yours sincerely,
Yujing Suo and Jianmin Fu

Response to Reviewer:

Reviewer #1: Suo et al report a chromosome scale assembly of D. oleifera, a diploid relative of hexaploid persimmon. They used a combination of Illumina, 10x, PacBio, and HiC to generate the chromosome scale assembly D. oleifera. The inclusion of high coverage Illumina data and scaffolding with 10x likely ensured that most of the residual indels from the PacBio only assembly were corrected. The HiC contact map in Figure 2 has no obvious inversions or misplacements, suggesting the genome is well assembled. This resource will be useful for the comparative genomics and persimmon research communities. I have a few minor concerns that should be addressed before this manuscript is published.

Q1. The estimated heterozygosity of D. oleifera is quite high (1.1%) and this would have likely resulted in assembly issues related to haplotype specific contigs. How many primary and alternate contigs were assembled by FALCON?
A: There were 2,986 contigs initially assembled by FALCON (Table 1).

Q2.Akagi et al. (https://www.biorxiv.org/content/early/2019/05/05/628537.full.pdf) report a chromosome scale assembly of diploid persimmon (D. lotus). The authors could cite this preprint in their manuscript and if the genome is publicly available, survey macrosynteny.
A: Thanks for your suggestion. We have added the macrosynteny analysis (Revised manuscript, Page12, Line 291-297). The chromosome-based macrosynteny analysis revealed a striking correspondence between D.oleifera and D.lotus, for that there were totally 432 syntenic blocks showed in supplementary Fig. S5.

Q3. The identification of homologs of sex determination genes from kiwi and D. lotus is not informative, as the kiwi sex determination system is likely completely different from D. oleifera and sex chromosomes may have an independent origin in D. lotus and D. oleifera. It is fine to leave this in the paper, but the statement that candidate sex determination genes were identified should be removed from the abstract
A: Thanks for your suggestion. We have removed the content about sex determination, and focused on the analysis of genes related to tannin synthesis.

Q 4. The identification of a WGD event in D. oleifera is interesting, and figure S2 could probably be moved to the main text. Based on this figure, it looks like there could have been two WGD events in D. oleifera.
A: Thanks for your suggestion. We have moved the Fig. S2 to the main text as the new Fig. 5. Besides the ancient γ event (all core eudicots share an ancient WGD, 4dtv = 0.66), a second WGD event occurred in D.oleifera and D.lotus (4dtv=0.36 ~0.27-0.42) which might contribute to the divergence of Ebenaceae with A. chinensis and C. sinensis. (Revised manuscript, Page12, Line 289-292)

Minor
Page 6, line 152. homologous should not be use here
Versions are provided or most but not all bioinformatics software. Where appropriate, versions should be added.
A: 'homologous' in Page 6, line 152 was deleted and versions of bioinformatics software had been provided in the article. (Revised manuscript, Page7, Line 165-166)

Reviewer #2: This manuscript describes the assembly of the first chromosome-level genome sequence of an Ebenaceae, Diospyros oleifera. The newly generated genome sequence was analysed for TE and gene content as well as for tannin synthase and sex determination genes. A phologenetic tree was constructed for divergence time estimation.

Data preparation as well as the construction of the pseudomolecules follows established and proven protocols and the results look good to me. Same is true for the gene model prediction and TE detection.

My main issue with this study is that it is almost exclusively a description of a newly established genomic resources, with very little to no new biological insights included in this manuscript. There is a little bit on tannin synthase and sex determination but this is all based on existing knowledge and little more than a homolog search. I appreciate the generation of these novel and helpful resources but these data could/should have been used to gain more biological insights.
A : For the question you mentioned, we supplemented the content of comparative genomic analysis in the revised manuscript, including gene family cluster, expansion and contraction of gene families, positively selection analysis, and macrosynteny analysis. Some interesting results were found: Firstly, compared with D.lotus, C.sinensis, and V.vinifera, CHS (Chalcone synthase) genes (the first key enzyme in the flavonoid-anthocyanin pathway) had different degrees of expansion in D.oleifera genome, which may be contribute to the abundant tannin production in the D.oleifera. Besides, CHI (Chalcone isomerase) gene (another key enzyme in the flavonoid-anthocyanin pathway) was found to be positive selected. These results will contribute to the molecular mechanism analysis of the major economic traits in Diospyros. Furthermore, the divergence time of between D. oleifera and D.lotus was estimated at 9.0 Mya, and two WGD events occurred in D.oleifera genome. These results will be helpful for the further analysis of the evolution of Diospyros species, and the origin of hexaploid persimmon.
In brief, this study provides a high-quality chromosomal level assembly of D.oleifera genome, which will provide important data support for the assembly of subsequent hexaploid persimmon genomes and the molecular mechanism analysis of the major economic traits in Diospyros.

More specific issues:
a.) Transcriptome data: I could not find a proper description of the transcriptome data that was obviously generated with this study and used for gene prediction. This could e.g. also be used to establish transcription levels for gene or gene families of interest.
A：The description of the transcriptome data was displayed in the 'Genomic RNA extraction, library construction and sequencing' part of the article. (Revised manuscript，Page7，Line 156-160)

b.) Functional annotation: I would recommend to use more specialized tools such as AHRD or BLAST2GO instead of simple best Blast hit for the human readable descriptions.
A：Thanks for your suggestion. We had reannotated the gene set by BLAST2GO, as a result，19,900 genes were annotated. After combining with the earlier annotation by blastp with InterPro database, there were totally 20,826 genes that had GO annotation, account for 68.20% of the gene models. The results were showed in the article. (Revised manuscript，Page8，Line 186-191)

c.) Phylogeny: I would recommend to use OrthoFinder instead of the older OrthoMCL version for determining the orthologous groups. Also, I'm not sure about the bias possibly introduced by just picking the single copy orthologs for the construction of the phylogenetic tree.
A：Thanks for your suggestion. We had reanalysis the gene families with OrthoFinder and got totally 19,722 clusters which were used for the phylogeny construction. The results were displayed in the article. (Revised manuscript，Page9，Line 224-230)

d.) I could not access any data under the NCBI accession number given.
A：We confirmed the NCBI data link, you and the reviewers can find the data under this link: https://dataview.ncbi.nlm.nih.gov/object/PRJNA532832?reviewer=gbss3pp9p06h5hosks3vrepirg

e.) Language and grammar needs improvement.
A：The English in this document has been checked by at least two professional editors, both native speakers of English. For a certificate, please see: http://www.textcheck.com/certificate/Q48QsC

Close
