# Supplementary material for: A high-quality chromosomal genome assembly of Diospyros oleifera Cheng
Source: Gigascience. 2020 Jan 16;9(1):giz164. doi: 10.1093/gigascience/giz164 (PMC6964648; doi:10.1093/gigascience/giz164)
Supplement: giz164_Response_to_Reviewer_Comments_Revision_1 [file giz164_response_to_reviewer_comments_revision_1.pdf]

Author's Response To Reviewer Comments

Close

Dear Editor,

Thank you very much for offering us the opportunity to resubmit a revised version of our manuscript. Hereby, we submit the revised manuscript entitled “A high-quality chromosomal genome assembly of Diospyrosoleifera” (GIGA-D-19-00174) to GigaScience. We appreciate the valuable comments and suggestions from you and the reviewers, which help us to improve and clarify the manuscript. We have discussed the comments carefully and tried our best to improve the manuscript accordingly.

This study focuses on the genome sequencing, assembly and annotation of D. oleifera, moreover comparative genomic analyses with other species were also included. The purpose of this study is to provide accurate genomic information for the further studies on molecular mechanisms underlying the formation and regulation of important economic traits of Diospyros spp. Based on this present study, some biological issues such as sex differentiation of flowers and natural deastringence of fruits in persimmon are being studied, the results of which will be reported in the future. Additionally, we heard that a similar study has been conducted by another research group, and they have submitted their manuscript to another journal. Due to this information, we deeply appreciate that if the review progress of our manuscript could be accelerated.

Detailed responses to your and the reviewers’ comments are provided in the next sections. We hope these responses are satisfactory and that the revised version will be acceptable for publication.

Please do not hesitate to contact us with any questions and we are looking forward to your reply.

Thanks and Best wishes!

Yours sincerely,
YujingSuo and Jianmin Fu

Response to Reviewer:

Reviewer: The authors complemented their study with a number of comparative genome analyses which indeed provide some, but limited novel biological insights. Some points from the first review round should be addressed or at least there should be someexplanation why these issues are not relevant:

Q1. transcriptome data: I appreciate the addition of the paragraph on extraction, library construction and sequencing but I still wonder why these data are not used in the analysis, eg. to establish transcription levels for gene or gene families of interest. It seems that the data may be of good quality, multiple tissues etc but there is no statistics or data description anywhere. Will/is the transcriptome data deposited in some public archive?

A: This article focuses on the genome sequencing, assembly, annotation of D. oleifera, and comparative genomic analyses with other species. The purpose of this article is to provide accurate genomic information for the studies of molecular mechanisms underlying the formation and regulation of important economic traits in Diospyros spp. Thus, the transcriptome data is mainly used for genomic annotation. Additionally, the transcriptome data is also used to analyze the expression patterns of CHS genes in different tissues of D. oleifera. (Revised manuscript, Page 12, Line288-291).
The transcriptome data is deposited in NCBI, you can find the data under this link:
https://dataview.ncbi.nlm.nih.gov/object/PRJNA532832?reviewer=gbss3pp9p06h5hosks3vrepirg

Q2.Phylogeny: how about the bias possibly introduced by just picking the single copy orthologs for the construction of the phylogenetic tree? This set is just a very small subset of the full gene content. To me

lines 260/261, and thus construction of the phylogeny, are largely unclear.
A: Gene families were generated by Orthofinder. After clustering, 19,722 gene families were detected across D.oleifera and 11 other species, of which 5,599 gene families and 221 single-copy orthologs were shared by 12 species. A phylogenetic tree of the 12 plant species was constructed using Orthofinder based on phylogenetic tree constructed by FastME. Gene trees were inferred for each orthogroup by aligning the sequences using mafft-linsi and inferring a maximum likelihood tree from this alignment using FastTree. DLCpar was used to reconcile these gene trees with the known species tree. In addition, 221 single-copy orthologs were used to estimate divergence time, rather than construct the phylogenetic tree.

Q3. Gene families: I'm not sure whether there is any biological conclusion on the genes and enrichments that were identified as D.oleifera specific? Can the terms be related to any biological features?
A: Using GO term enrichment analysis, we performed functional annotation on the D.oleifera specific genes. As a result, only 98 of the 312 genes had conserved functional terms which were significantly enriched for zinc ion bingding, proteolysis, and nutrient reservoir activity. Moreover, 4 and 1 of these genes were involved in the carbohydrate metabolic process and aldehyde metabolic process respectively, which may play roles in the carbohydrate accumulation and deastringency of fruit in D.oleifera.

Q4. Expansion/Contraction: what parameters where used for CAFÉ? CHS expansion results should be outlined in the text. What does "different degrees of expansion" mean? An obvious additional and worthwhile analysis would be check expanded/contracted gene families for their expression patterns. What is the conclusion of LAC gene family contraction?
A: (1) For CAFÉ parameter Settings: Gene families with size significantly changed for species/branch: viterbi p<=0.05, and the others are the default parameters.
(2) The description of these results has been revised as follow: compared with D. lotus, C. sinensis, and V. vinifera, chalcone synthase (CHS) genes expanded in the D. oleifera genome (11 genes in D.oleifera, 7 genes in D.lotus, 3 genes in C. sinensis, and 1 gene in V. vinifera; P_value = 0.0089). In addition, Using transcriptome data, CHS gene expression patterns in different tissues of persimmon were analyzed. (Revised manuscript, Page 12, Line 284-291)
(3) Laccase (LAC) genes were responsible for the polymerization of persimmon tannin monomers. The contraction of these genes may explain the difference of tannin types which were defined according to the polymerization level of tannin monomers between D. oleifera and V. vinifera.

Q5. Positively selected genes: I'm really not sure about the significance of this analysis. Are the terms identified somewhat related to any biological features?
A: Positively selection analysis was used to study the adaptive evolution of genes, which could help us better understand the evolution of D.oleifera. In this study, 186 genes were positively selected in D. oleifera compared with D. lotus, A. chinensis, P. veris, R. delavayi and S. lycopersicum. Among them, chalcone isomerase (CHI) gene, a key enzyme in the flavonoid-anthocyanin pathway, was found to be positively selected (ID:evm.model.original_scaffold_909.101). The positive selection of CHI gene may be one of the reasons why D. oleifera is different from other species in producing abundant tannin. (Revised manuscript, Page13, Line 305-307)

Q6. Please check the formats and structure of your files provided. Testing the GFF files with Gff3Validator results in an error for example: gt gff3validator Dol.gff3 gt gff3validator: error: child on line 44626 in file Dol.gff3" has different sequence id than its parent on line 44625 ('Chr4' vs. 'fragScaff_scaffold_95:::fragment_2:::debris')
A: Thank you for pointing this out, we have checked the formats and structure of our files, and corrected the error. The revised files have been re-uploaded to the system.

Q7.Especially the newly added text needs significant improvement in language and grammar.
A: The English in this revised manuscript has been checked by at least two professional editors, both native speakers of English. For a certificate, please see: http://www.textcheck.com/certificate/FrAnnY

Close
