# Supplementary material for: A high-quality chromosomal genome assembly of Diospyros oleifera Cheng
Source: Gigascience. 2020 Jan 16;9(1):giz164. doi: 10.1093/gigascience/giz164 (PMC6964648; doi:10.1093/gigascience/giz164)
Supplement: giz164_Reviewer_1_Report_Original_Submission — Robert VanBuren -- 6/13/2019 Reviewed [file giz164_reviewer_1_report_original_submission.pdf]

Reviewer Report

Title: A high-quality chromosomal genome assembly of Diospyros oleifera

Version: Original Submission **Date:** 6/13/2019

Reviewer name: Robert VanBuren

Reviewer Comments to Author:

Suo et al report a chromosome scale assembly of D. oleifera, a diploid relative of hexaploid persimmon. They used a combination of Illumina, 10x, PacBio, and HiC to generate the chromosome scale assembly D. oleifera. The inclusion of high coverage Illumina data and scaffolding with 10x likely ensured that most of the residual indels from the PacBio only assembly were corrected. The HiC contact map in Figure 2 has no obvious inversions or misplacements, suggesting the genome is well assembled. This resource will be useful for the comparative genomics and persimmon research communities. I have a few minor concerns that should be addressed before this manuscript is published.

1. The estimated heterozygosity of D. oleifera is quite high (1.1%) and this would have likely resulted in assembly issues related to haplotype specific contigs. How many primary and alternate contigs were assembled by FALCON?

2.Akagi et al. (https://www.biorxiv.org/content/early/2019/05/05/628537.full.pdf) report a chromosome scale assembly of diploid persimmon (D. lotus). The authors could cite this preprint in their manuscript and if the genome is publicly available, survey macrosynteny.

3. The identification of homologs of sex determination genes from kiwi and D. lotus is not informative, as the kiwi sex determination system is likely completely different from D. oleifera and sex chromosomes may have an independent origin in D. lotus and D. oleifera. It is fine to leave this in the paper, but the statement that candidate sex determination genes were identified should be removed from the abstract

4. The identification of a WGD event in D. oleifera is interesting, and figure S2 could probably be moved to the main text. Based on this figure, it looks like there could have been two WGD events in D. oleifera.

Minor

Page 6, line 152. homologous should not be use here

Versions are provided or most but not all bioinformatics software. Where appropriate, versions should be added.

Level of Interest

Please indicate how interesting you found the manuscript: Choose an item.

Quality of Written English

Please indicate the quality of language in the manuscript: Choose an item.

Declaration of Competing Interests

Please complete a declaration of competing interests, considering the following questions:

- Have you in the past five years received reimbursements, fees, funding, or salary from an organisation that may in any way gain or lose financially from the publication of this manuscript, either now or in the future?
- Do you hold any stocks or shares in an organisation that may in any way gain or lose financially from the publication of this manuscript, either now or in the future?
- Do you hold or are you currently applying for any patents relating to the content of the manuscript?
- Have you received reimbursements, fees, funding, or salary from an organization that holds or has applied for patents relating to the content of the manuscript?
- Do you have any other financial competing interests?
- Do you have any non-financial competing interests in relation to this paper?

If you can answer no to all of the above, write 'I declare that I have no competing interests' below. If your reply is yes to any, please give details below.

I declare that I have no competing interests

I agree to the open peer review policy of the journal. I understand that my name will be included on my report to the authors and, if the manuscript is accepted for publication, my named report including any attachments I upload will be posted on the website along with the authors' responses. I agree for my report to be made available under an Open Access Creative Commons CC-BY license (http://creativecommons.org/licenses/by/4.0/). I understand that any comments which I do not wish to be included in my named report can be included as confidential comments to the editors, which will not be published.

Choose an item.

To further support our reviewers, we have joined with Publons, where you can gain additional credit to further highlight your hard work (see: https://publons.com/journal/530/gigascience). On publication of this paper, your review will be automatically added to Publons, you can then choose whether or not to claim your Publons credit. I understand this statement.

Yes Choose an item.
