# Supplementary material for: A high-quality chromosomal genome assembly of Diospyros oleifera Cheng
Source: Gigascience. 2020 Jan 16;9(1):giz164. doi: 10.1093/gigascience/giz164 (PMC6964648; doi:10.1093/gigascience/giz164)
Supplement: giz164_Reviewer_2_Report_Revision_1 — Manuel Spannagl -- 9/26/2019 Reviewed [file giz164_reviewer_2_report_revision_1.pdf]

Reviewer Report

Title: A high-quality chromosomal genome assembly of Diospyros oleifera

Version: Revision 1 **Date:** 9/26/2019

Reviewer name: Manuel Spannagl

Reviewer Comments to Author:

The authors complemented their study with a number of comparative genome analyses which indeed provide some, but limited novel biological insights.
Some points from the first review round should be addressed or at least there should be some explanation why these issues are not relevant:
1.) transcriptome data: I appreciate the addition of the paragraph on extraction, library construction and sequencing but I still wonder why these data are not used in the analysis, eg. to establish transcription levels for gene or gene families of interest. It seems that the data may be of good quality, multiple tissues etc but there is no statistics or data description anywhere. Will/is the transcriptome data deposited in some public archive?
2.) Phylogeny: how about the bias possibly introduced by just picking the single copy orthologs for the construction of the phylogenetic tree? This set is just a very small subset of the full gene content. To me lines 260/261, and thus construction of the phylogeny, are largely unclear.
3.) Gene families: I'm not sure whether there is any biological conclusion on the genes and enrichments that were identified as D.oleifera specific? Can the terms be related to any biological features?
4.) Expansion/Contraction: what parameters where used for CAFÃ‰? CHS expansion results should be outlined in the text. What does "different degrees of expansion" mean? An obvious additional and worthwhile analysis would be check expanded/contracted gene families for their expression patterns. What is the conclusion of LAC gene family contraction?
5.) Positively selected genes: I'm really not sure about the significance of this analysis. Are the terms identified somewhat related to any biological features?
6.) Please check the formats and structure of your files provided. Testing the GFF files with Gff3Validator results in an error for example:â€¨â€¨gt gff3validator Dol.gff3â€¨gt gff3validator: error: child on line 44626 in file Dol.gff3" has â€¨different sequence id than its parent on line 44625 ('Chr4' vs. â€¨'fragScaff_scaffold_95:::fragment_2:::debris')
7.) Especially the newly added text needs significant improvement in language and grammar.

Level of Interest

Please indicate how interesting you found the manuscript: Choose an item.

Quality of Written English

Please indicate the quality of language in the manuscript: Choose an item.

Declaration of Competing Interests

Please complete a declaration of competing interests, considering the following questions:

- Have you in the past five years received reimbursements, fees, funding, or salary from an organisation that may in any way gain or lose financially from the publication of this manuscript, either now or in the future?
- Do you hold any stocks or shares in an organisation that may in any way gain or lose financially from the publication of this manuscript, either now or in the future?
- Do you hold or are you currently applying for any patents relating to the content of the manuscript?
- Have you received reimbursements, fees, funding, or salary from an organization that holds or has applied for patents relating to the content of the manuscript?
- Do you have any other financial competing interests?
- Do you have any non-financial competing interests in relation to this paper?

If you can answer no to all of the above, write 'I declare that I have no competing interests' below. If your reply is yes to any, please give details below.

I declare that I have no competing interests

I agree to the open peer review policy of the journal. I understand that my name will be included on my report to the authors and, if the manuscript is accepted for publication, my named report including any attachments I upload will be posted on the website along with the authors' responses. I agree for my report to be made available under an Open Access Creative Commons CC-BY license (http://creativecommons.org/licenses/by/4.0/). I understand that any comments which I do not wish to be included in my named report can be included as confidential comments to the editors, which will not be published.

Choose an item.

To further support our reviewers, we have joined with Publons, where you can gain additional credit to further highlight your hard work (see: https://publons.com/journal/530/gigascience). On publication of this paper, your review will be automatically added to Publons, you can then choose whether or not to claim your Publons credit. I understand this statement.

Yes Choose an item.
